# Machine learning-based integration of transcriptome and digital pathology for predicting chemoresistance in muscle-invasive bladder cancer

**DOI:** 10.1038/s12276-026-01718-y

**Published:** 2026-05-08

**Authors:** Jinahn Jeong, Gowun Jeong, YongHwan Kim, Hyein Ju, Hyun Jun Im, Hyun Ji Kim, Ja-Min Park, Se Un Jeong, Seungun Lee, Min Gi Jang, Yun Ji Nam, Hyungu Kwon, Seok Woo Ha, Siwon Lee, Dabin Lee, Eunyoung Park, Sung Jin Kim, Inkeun Park, Jae Lyun Lee, Bumsik Hong, Yong Mee Cho, Dong-Myung Shin

**Affiliations:** 1 Department of Pathology, Asan Medical Center, University of Ulsan College of Medicine, Seoul, Republic of Korea; 2 AI Model Development Team, Engineering Development Division, TES Research, CJ Logistics, Seoul, Republic of Korea; 3 Department of Cell and Genetic Engineering, Asan Medical Center, Brain Korea 21 project, University of Ulsan College of Medicine, Seoul, Republic of Korea; 4 Department of Pathology, Kyung Hee University Hospital, Kyung Hee University College of Medicine, Seoul, Republic of Korea; 5 AinB Inc, Asan Institute for Life Sciences, Seoul, Republic of Korea; 6 Department of Urology, Gangneung Asan Hospital, University of Ulsan College of Medicine, Gangwon-do, Republic of Korea; 7 Department of Oncology, Asan Medical Center, University of Ulsan College of Medicine, Seoul, Republic of Korea; 8 Department of Urology, Asan Medical Center, University of Ulsan College of Medicine, Seoul, Republic of Korea

**Keywords:** Bladder cancer, Tumour heterogeneity, Tumour biomarkers

## Abstract

Muscle-invasive bladder cancer (MIBC) presents with variable clinical and pathological features, leading to inconsistent responses to standard treatments such as neoadjuvant chemotherapy (NAC). Although transcriptome profiling has shown differences in NAC response, reliable predictors of treatment outcome remain elusive. Here this study aimed to improve NAC response prediction by integrating multicohort transcriptomic data and spatial protein expression profiles using machine learning, enabling precision diagnostics and therapeutic strategies. Transcriptome analysis from four independent cohorts (*n* = 399) using diverse gene classifiers revealed molecular features associated with NAC response, particularly genes involved in stress responses, immunity and cell adhesion. The clinical relevance of 74 markers was validated by digital pathology for analyzing spatial protein expression. The machine learning frameworks reduced complex transcriptome and digital pathology datasets to a clinically manageable number of biomarkers, yielding an optimal antibody panel for immunohistochemistry-based clinical diagnostics. Computational pathology-driven predictions of NAC response demonstrated a strong correlation with survival outcomes in patients with MIBC, highlighting their potential clinical utility. Mechanistically, targeting the KEAP1–NRF2 axis suppressed glutathione dynamics, proliferation, stemness features and invasiveness of cisplatin-resistant MIBC cells, thereby resensitizing them to cisplatin. Combination treatment with cisplatin and inhibitors targeting the KEAP1–NRF2 pathway markedly suppressed tumor growth in an orthotopic xenograft model. Therefore, this study integrates machine learning-based transcriptome profiling and digital pathology analysis to refine gene classifiers, provide a personalized and feasible framework for treatment decision-making, and overcome chemoresistance to improve therapeutic efficacy.

**Figure:**
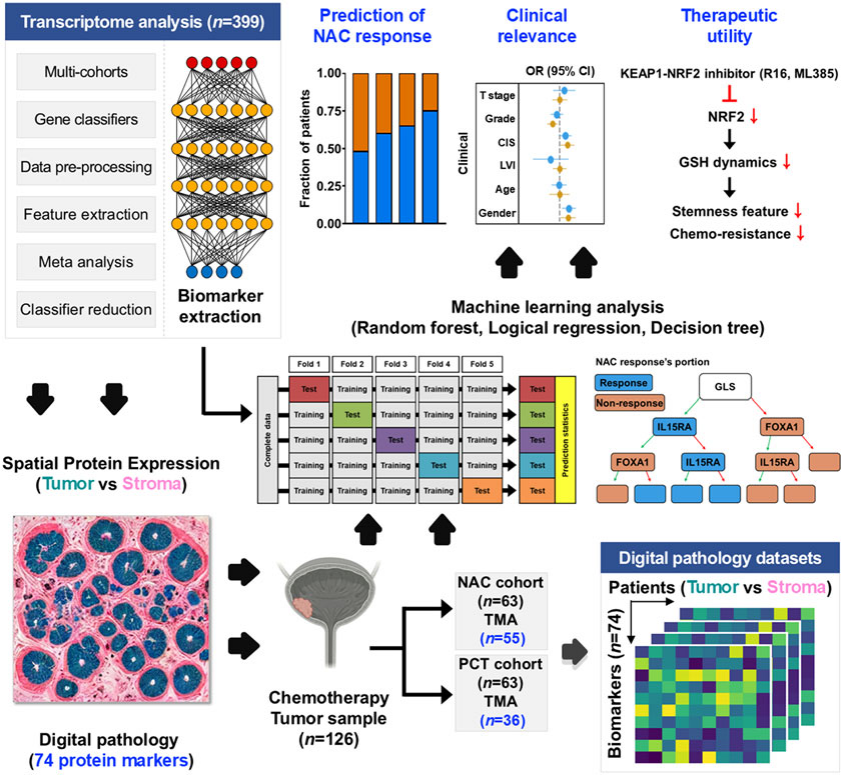
This study integrates machine learning with transcriptome and digital pathology data to identify and validate predictive biomarkers for neoadjuvant chemotherapy response in muscle-invasive bladder cancer. The optimized biomarkers, along with a proposed antibody combination, may improve precision medicine approaches. The KEAP1–NRF2 pathway was identified as a potential therapeutic target.

This study integrates machine learning with transcriptome and digital pathology data to identify and validate predictive biomarkers for neoadjuvant chemotherapy response in muscle-invasive bladder cancer. The optimized biomarkers, along with a proposed antibody combination, may improve precision medicine approaches. The KEAP1–NRF2 pathway was identified as a potential therapeutic target.

## Precision diagnostics transform neoadjuvant chemotherapy outcomes

Bladder cancer, particularly muscle-invasive bladder cancer (MIBC), presents a substantial clinical challenge due to its aggressive nature and heterogeneous response to neoadjuvant chemotherapy (NAC). This study addresses the need for reliable predictive markers to identify patients who will benefit from NAC. Researchers utilized machine learning to analyze transcriptome datasets and digital pathology, identifying key proteins predictive of NAC response. They validated these findings using computational pathology and explored the role of the KEAP1–NRF2 pathway in chemoresistance. The results showed that targeting this pathway could enhance cisplatin sensitivity in MIBC, offering a potential therapeutic strategy. The study advances our understanding of MIBC treatment and suggests future directions for personalized therapy, emphasizing the integration of machine learning and multimodal data for clinical applications. This summary was initially drafted using artificial intelligence, then revised and fact-checked by the author.

## Introduction

Bladder cancer (BC) is characterized by highly heterogeneous clinical and pathological features, which are responsible in part for frequent treatment failure, including high rates of recurrence and poor outcomes in patients with advanced disease^1,2^. Muscle-invasive BC (MIBC) is an aggressive form that can rapidly progress to metastatic disease and has a high mortality rate^3^. Following transurethral resection of the bladder tumor (TURBT) for diagnostic and therapeutic purposes, radical cystectomy with preoperative cisplatin-based neoadjuvant chemotherapy (NAC) is the current standard of care for MIBC. However, the response to NAC remains unsatisfactory and a pathological complete response, which is a surrogate marker for reduced risk of death and recurrence, is achieved in 30–40% of patients^4,5^. The clinicopathologic features of patients with MIBC and molecular stratification are not adequate for prospectively identifying patients likely to benefit from NAC^6^. Therefore, there is an urgent need to develop reliable predictive markers to identify patients who are most likely to respond favorably to NAC.

Advances in transcriptome profiling have facilitated the molecular stratification of MIBCs with unique biological characteristics and variance in clinical behaviors including the response to NAC. For instance, patients with the p53-like molecular subtype in TURBT specimens show a poor response to NAC^7^. Patients with basal-like tumors benefit markedly from NAC, showing higher overall survival (OS) rates than an unmatched patient cohort treated with surgery alone^8^. Despite these encouraging results, the development of trustworthy and affordable molecular subtyping algorithms has been hampered by the complexity of transcriptome profiling methodologies and the heterogeneity of tumor and stromal compartments; the clinical utility and robustness of transcriptome-based molecular classifications for predicting NAC response thus remains controversial^6,9,10^. These disparate results suggest the need for further refinement and validation of molecular stratification approaches.

Immunohistochemistry (IHC) has become a popular substitute for mRNA-based subtyping. IHC enables the visualization of protein expression in situ in tissue samples, thereby providing critical information on the molecular characteristics of the tumor. IHC is relatively less affected by sample-to-sample variation in tumor and stromal compartments and can define the major subtypes of MIBC. IHC has been used to identify luminal and basal mRNA subtypes, as exemplified by the Lund taxonomy classification of BC, which evolved from mRNA profiling to IHC-defined subtyping^11^. However, the clinical applicability of IHC demands a concise set of markers that robustly reflect the biological behavior and therapeutic response, facilitating practical implementation in routine diagnostic workflows. Furthermore, IHC analysis is laborious and subjective, and analyzing diverse antigens in the tumor and stromal compartments from multiple patients using high-throughput automated systems is difficult. These limitations can be overcome by digitizing glass slides and using computational pathology, which provides quantitative and reproducible analysis of histological data, thereby increasing the accuracy of the analysis and enabling molecular subtyping for tumor characterization and clinical association^12,13^.

In previous work, we used high-throughput digitalized IHC analysis with a machine learning-based tumor/stroma classifier, and demonstrated that glutathione (GSH) dynamic proteins, including glutaminase-1 (GLS), were associated with NAC resistance in MIBCs^14^. In line with this observation, one emerging mechanism of chemoresistance in aggressive tumors involves dysregulation of the Kelch-like ECH-associated protein-1 (KEAP1) and nuclear factor erythroid 2-related factor-2 (NRF2) mediated oxidative stress pathway^15^. Under physiological conditions, KEAP1 binds NRF2 and targets it for ubiquitin-mediated proteasomal degradation, thus suppressing the expression of cytoprotective gene. Loss-of-function alterations in KEAP1 (or activating mutations in NRF2) allow tumor cells to hijack this pathway, leading to constitutive NRF2 activation, elevated antioxidant defenses (for example, GSH biosynthesis and detoxification enzymes) and enhanced tolerance to chemotherapy-induced oxidative injury^15^. Notably, KEAP1-inactivated cancers often become addicted to such redox homeostasis adaptations. For example, KRAS-driven lung tumors with KEAP1 mutations develop an increased dependency on glutamine-fueled GSH synthesis, a vulnerability that can be therapeutically exploited with glutaminase inhibitors^16^. Conversely, restoring control of the KEAP1–NRF2 axis has shown promise in reversing chemoresistance^17–19^. Accordingly, pharmacological inhibition of the KEAP1–NRF2 axis has emerged as a promising therapeutic strategy.

In addition to identifying the potential therapeutic targets, integrating machine learning approaches with multimodal omics datasets has emerged as a promising strategy to enhance the predictive accuracy of the NAC response^12,20–22^. Machine learning algorithms are adept at handling complex, high-dimensional data and can uncover intricate patterns and interactions by integrating traditional analytical methods^20,23,24^. This enables the development of precise and reliable predictive models to guide the design of personalized treatment strategies, which may improve the clinical outcomes of patients with MIBC. In this study, we established a machine learning analysis workflow to identify candidate proteins predictive of NAC response using both in-house and external cohort transcriptome datasets and molecular classifiers and validated their clinical relevance using computational pathology. Digital pathology datasets comprising the validated 74 protein markers in 55 cases of localized MIBC treated with NAC (NAC cohort) and 36 cases of advanced MIBC treated with preoperative chemotherapy (PCT cohort) were analyzed through machine learning computation to determine the optimal combination for IHC analysis, paving the way for the development of straightforward, precise and clinically applicable NAC prediction algorithms. Furthermore, our machine learning platform provided a mechanistic insight for targeting KEAP1–NRF2 pathway as a therapeutic strategy for overcoming cisplatin resistance in MIBC.

## Materials and methods

### Patient sample collection and cohort organization

This study was approved by the Institutional Review Board of Asan Medical Center (AMC; 2020-0064). Patients gave informed consent for the use of samples and relevant clinical data for academic purposes. The study included 63 consecutive patients with MIBC (cT2-4aN0M0) who underwent TURBT followed by NAC and curative surgery with available formalin-fixed and paraffin-embedded tumor tissues between August 2011 and August 2017 at AMC, as previously described^14^. An independent AMC PCT cohort comprising 63 advanced patients with MIBC (>cT2-4aN0M0) enrolled during the same study period was also analyzed. Patient demographic data, including median age, gender distribution and NAC treatment regimens, are provided in Supplementary Table 1. Clinical information, such as NAC details, surgical TURBT records, tumor progression and survival time, was retrieved from electronic medical records. Progression free survival (PFS) and OS were estimated using the Kaplan–Meier method, and comparisons were made using the log-rank test. Additional information on the AMC cohort patients, including the criteria for pathological review and the determination of NAC responses, can be found in a previous study^14^ and in Supplementary Tables 2 and 3.

### Data preprocessing of the transcriptome datasets and optimization of the classifiers

Transcriptome analysis of the AMC discovery cohort was performed using a random forest modeling process by creating 10 random forests (each consisting of 100 trees) from 16 cases considering the small number of patients. The average ‘prediction score’ (NAC nonresponse (NR) = 0 and NAC response (R) = 1) and the average importance values of genes used in the model were calculated for each of the 10 models. A hierarchically clustered heat map analysis based on the top 5–30 combinations of genes was used to extract approximately 20 genes as a data-driven classifier that could distinguish between NR and R patients. The performance of the gene classifiers derived from the previous reports was compared by calculating the importance of genes using random forests, and the top 5–30 combinations obtained by heat map clustering with ordering by gene importance were used to identify key genes for each classifier. The selected combination of genes was used for logistic regression modeling to determine the prediction scores for each patient in the AMC discovery cohort.

For the classifier reduction process, data preprocessing was performed using the Power Transformer (Yeo-Johnson) scaler. The mutual information and variance inflation factor were used to eliminate variables with weak discriminatory power within the classifiers, followed by hierarchical clustering to refine the selection of genes, which notably differentiated between the R and NR groups. The selected genes within each classifier were then used for logistic regression modeling.

### Modeling of the transcriptome datasets by cohort cross-validation

For cross-validation between AMC and MDA MVAC cohorts^7^, data were preprocessed to follow a Gaussian distribution using power transformation to address scale differences between two datasets. The classifiers were then refined by selecting genes present in both cohorts and exhibiting similar distribution, as determined by a *t*-test. When more than ten variables remained, random forest was used to extract no more than ten key genes. The refined classifiers were used to apply three algorithms (logistic regression, decision tree and random forest) to the power-scaled datasets, alternating between training and validation cohorts to compare the performance of the models and classifiers for the stratification of NAC response in both cohorts.

For multicohort cross-validation, to correct for differences between the four cohort datasets, genes common to all datasets were extracted and nine scaling algorithms (log2, log2_minmax, standard, minmax, max_abs, robust, power, quantile and RankGauss) were tested. Standard, power and RankGauss scalers, which follow a Gaussian distribution, were used for further analysis. Random forest-based premodeling was performed on all combinations of datasets and classifiers using these three scaling methods to evaluate their performance in terms of accuracy, recall, precision, F1 and Matthews correlation coefficient. The power scaler was identified as the optimal method.

The key variables in the classifiers comprising <10 genes were optimized. In addition, union classifiers were constructed by combining the top 10 genes optimized for each classifier in cohort datasets used in the analysis. The top 10 or union classifiers (two types of classifiers) constructed for each of the 17 classifiers were used for logistic regression, decision tree and random forest modeling (three models) in 12 combinations of four cohort datasets (AMC and three external cohorts). Each combination involved one cohort for training, one for validation, and two for testing, resulting in a total of 1,224 (2 × 17 × 3 × 12) cross-validations.

### Machine learning-based analysis of digital pathology datasets

The modeling process for the digital pathology dataset followed a structured approach. Two datasets were generated from the computational pathology values for the machine learning analysis: raw data (raw) and standardized data values. To address the limitations posed by the small number of measured biomarkers (*n* = 74), limited patient samples (55 NAC and 36 PCT cases) and different clinical characteristics between the training and validation groups, a data-driven classifier process was used, namely, the recursive feature elimination with cross-validation (RFECV) analysis method. This method was used to rank features, starting with the full set of variables and iteratively removing one feature at a time. At each iteration, a model was trained and evaluated; if the removal of a specific feature improved model performance, that feature was assigned a higher rank. Conversely, features whose removal did not enhance performance were assigned lower ranks. This iterative process was performed within a cross-validation framework to ensure robust and reliable feature rankings. Decision tree and logistic regression were used as the modeling methods for this iterative process to evaluate feature importance and predictive performance.

To identify the optimal predictive model, a stepwise approach was used with gene pools derived from the RFECV based data-driven classifier process. This process led to the extraction of ten gene pools comprising five genes from tumor data and five genes from stromal data. For each gene pool, the number of effective markers was iteratively changed between two and five. Two base modeling approaches, decision tree and logistic regression, were applied to assess model performance at each iteration. The combined dataset, consisting of training data (NAC cohort) and validation data (PCT cohort), was subjected to threefold cross-validation. During this process, the optimal combination of markers corresponding to the specified number of effective markers was determined.

Feature selection was performed using both forward selection and backward elimination methods. In the forward selection process, markers were sequentially added from the pool of available markers. At each step, the marker that maximized performance when combined with previously selected markers was added until the desired number of effective markers was reached. The backward elimination method started with the full set of markers, sequentially removing one marker at a time. At each step, the marker whose removal optimized the model’s performance was excluded until the desired number of markers remained. If a new model meeting the predefined performance criteria was identified during this process, the current model was replaced with the newly identified optimal model. This iterative procedure ensured the systematic evaluation and selection of the best-performing model for each gene pool.

### IHC staining and digital pathology analysis

Tissue microarray (TMA) construction and IHC analysis were performed as previously described^14^. Detailed information on antibodies and staining conditions is provided in the key resources table. For computational pathology in the spatial protein expression analysis, IHC slides were scanned using a Pannoramic 250 Flash slide scanner (3D HISTECH) at ×20 magnification with a resolution of 0.22-μm per pixel and analyzed with QuPath, an open-source bioimage software. To evaluate expression intensities separately in tumor epithelial and stromal compartments, a machine learning classifier was developed to distinguish tumorous epithelial cells from stroma in 3,3′-diaminobenzidine (DAB) stained, hematoxylin counterstained images, as previously described^14^. The mean of three TMA cores per tumor was used to represent the expression level for tumor and stromal compartments.

### Analysis of clinical MIBC patient cohorts

Clinicopathological analysis of patients with MIBC in the AMC cohort was performed using SPSS version 21.0. All continuous data were compared using a Student’s *t*-test, and categorical data were compared with the Pearson’s Chi-square, Fisher’s exact and Kruskal–Wallis tests. Categorical IHC expression data were classified as high versus low expression of the indicated biomarkers based on the cutoff value, which was determined by the heuristic method. Univariate analyses of NAC response were performed to determine the clinical importance of each protein expression and clinicopathological parameter. Statistically significant variables from univariate analyses were analyzed by multivariate analyses, in which binary logistic regression was applied. Independent variables were chosen by backward stepwise selection, and *P* values <0.05 were considered statistically significant.

To classify patients based on the predictive models using the tumor and stromal biomarker expression, a model for tumor characteristics was constructed utilizing GLS, IL15RA and FOXA1, while stromal characteristics were modeled using TFEB, β-catenin, MTCH1 and CBS. Patients from the NAC and PCT cohorts were categorized into ‘Predicted as response group’ and ‘Predicted as nonresponse group’ based on the classification outcomes from each model for computation pathology. Kaplan–Meier survival analyses were conducted to compare OS and PFS between the predicted groups.

### Cell culture and in vitro functional assays

Human T24 (ref. ^25^) or J82 and KU19-19 MIBC cells (purchased from the University of Kent, The Resistant Cancer Cell Line collection) and their derivatives with differential cisplatin response (Cis_R) and nonresponse (Cis_NR) behaviors were maintained as previously reported^1,2,14^. Cells were treated with cisplatin (Sigma-Aldrich) or KEAP1–NRF2 inhibitors including ML385 (HY-100523, MedChemExpress) and R16 (HY-149508) for 24 h at the indicated concentrations. For overexpression of the human *KEAP1* open reading frame (ORF), pEZ-CMV lentiviral plasmids encoding human *KEAP1* (EX-M0487-Lv105) ORF constructs were purchased from GeneCopoeia and transduced into target cells, as previous described^14,26^. Detailed protocols for in vitro functional assays, including proliferation, apoptosis, tumor sphere formation, limiting dilution and invasion, are provided in the Supplementary Information.

### Orthotopic xenograft MIBC animal model

All animal experiments were approved by the Institutional Animal Care and Use Committee of the University of Ulsan College of Medicine (IACUC-2023-02-032) and conducted in accordance with institutional guidelines. Orthotopic bladder cancer xenograft models were established using cisplatin-resistant T24 MIBC cells in NOD/ShiLtJ-*Prkdc*^*em1AMC*^*Il2rg*^*em1AMC*^ (NSGA) mice. Mice were randomly assigned to treatment groups and received cisplatin, KEAP1–NRF2 pathway inhibitors or combination regimens. Tumor burden was monitored over 42 days and tissues were collected for histological and immunofluorescence analyses. All procedures including animal allocation, treatment and evaluation were performed in a randomized and blinded manner. Detailed experimental protocols are provided in the Supplementary Information.

### Statistical analysis

Quantitative data are expressed as the mean ± s.e.m. For comparisons involving more than three groups, statistical significance was assessed using one-way or two-way analysis of variance (ANOVA) followed by Bonferroni post hoc tests. For comparisons between two groups, unpaired Student’s *t*-tests were used. Statistical analyses were performed using GraphPad Prism 7.0 (GraphPad Software) or SPSS version 21.0. *P < 0.05, **P < 0.01 or ***P < 0.001 was considered statistically significant. All raw data quantification and statistical analyses, including the tests performed, sample sizes and exact *P* values, are available in the Source data, available as a separate Excel file.

Additional experimental procedures, including chromatin immunoprecipitation (ChIP), gene expression analysis, real-time live-cell imaging of glutathione recovery capacity (GRC) and immunofluorescence staining, are detailed in the Supplementary Information.

## Results

### Machine learning modeling for analyzing transcriptome datasets and gene classifiers

**Fig. 1:**
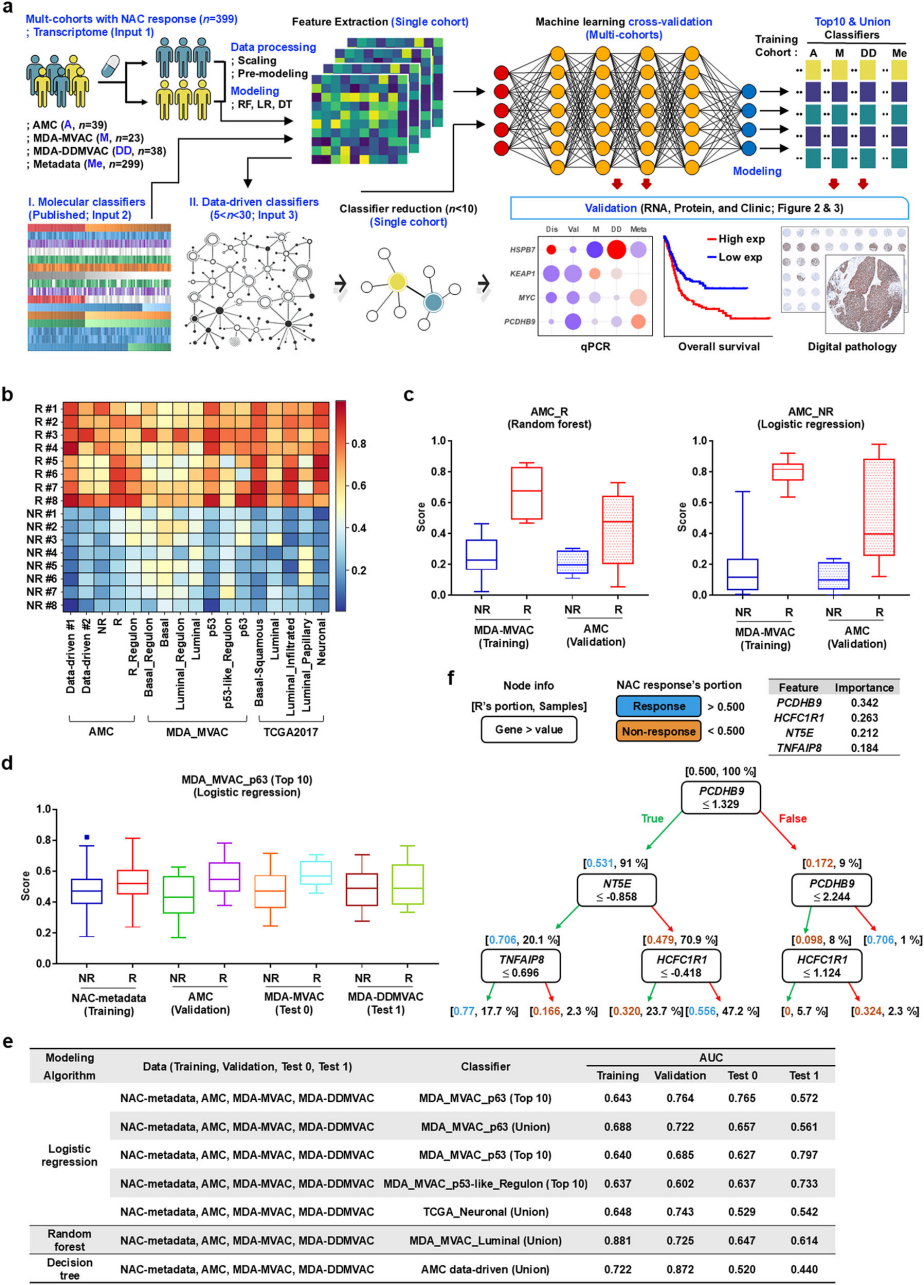
Machine learning models for analyzing transcriptome datasets and refining gene classifiers. **a** A schematic overview of the machine learning-based multicohort transcriptome analysis and gene classifier reduction for predicting the response to NAC in patients with MIBC. **b** A heat map summarizing the prediction scores (NR = 0 and R = 1) from the logistic regression model in the AMC discovery cohort (*n* = 8/group, NR and R) with the indicated gene classifiers. **c** Box plots of the prediction scores for the modeling algorithms, which showed robust performance in the cross-validation between the MDA MVAC (training) and AMC (validation) cohorts. **d**, **e** Multicohort cross-validation using 17 gene classifiers and the transcriptome datasets from AMC and three independent external cohorts, including publicly available gene expression profiling datasets from pre-NAC TURBT specimens and records of pathological response to NAC: box plots of the prediction scores for the logistic regression model with the top 10 MDA_MVAC_p63 gene classifier in the indicated cohort sets (**d**) and a comparison of the AUC for the modeling algorithms based on the indicated gene classifiers and combinations of four cohort datasets, including one cohort for training, one for validation and two for testing (**e**). **f** A decision tree depicting the stepwise classification process used for the gene classifier reduction process. Each internal node represents a decision rule based on the expression of the key feature genes, branches correspond to possible outcomes of the rule marked with different colors (true, green; false, red) and terminal nodes represent the predicted NAC response (R, blue; NR, orange) and the frequency of samples. The importance of key features is presented with the decision tree. Detailed information on the gene classifiers used for each modeling algorithm is presented in Supplementary Tables 4–7.

In previous work, we performed gene expression profiling of MIBC tumors from four independent cohorts to elucidate the biological basis of the molecular heterogeneity in the MIBC NAC response^14^. In this study, we used the transcriptome datasets and various molecular classifiers to establish a machine learning-based computation strategy with the capacity to (1) derive a data-driven classifier for NAC response prediction using single and multicohort cross-validation and (2) optimize the key variables in each gene classifier to select a small number, preferably less than 10, for further diagnostic and therapeutic applications (Fig. 1a). Initially, we used the discovery cohort of our institute (AMC), in which ‘incomplete TURBT’ was documented on the TURBT surgical record, indicating that residual tumor remained after TURBT, thereby excluding the possibility of complete tumor removal by TURBT before NAC and enabling precise assessment of the therapeutic response to NAC^14^. Transcriptome profiling was performed on 16 patients (NR, *n* = 8; R, *n* = 8) using laser capture microdissection (LCMD) of tumor cells to minimize contamination of stromal and inflammatory cells from formalin-fixed and paraffin-embedded TURBT specimens.

Given the small number of patients in the AMC discovery cohort, we used a random forest model and calculated the average ‘prediction score’ (NR = 0 and R = 1) for the ten random forest models generated (Supplementary Fig. 1a). The average importance values of the genes included in the model analysis (Supplementary Fig. 1b) was used to extract an AMC data-driven classifier comprising 25 genes (AMC data-driven classifier_#1) for stratifying two NAC response groups (Supplementary Table 4). Gene Ontology (GO) analysis of the genes in this classifier revealed key pathways related to cell cycle and proliferation (Supplementary Fig. 1c). The classifier was reduced to six genes (AMC data-driven classifier_#2) by (1) determining variable importance using the random forest model, (2) evaluating combinations of the top 5–30 variables (genes) according to importance ranking and (3) using cluster heat maps to identify the minimal set of genes that best distinguish between R and NR groups (Supplementary Table 4).

Next, different gene classifiers related to MIBC molecular stratification identified in previous studies were applied to the machine learning model to evaluate their performance in predicting the response to NAC, and highly important genes within each classifier were identified. We compared (1) five AMC classifiers, including two data-driven classifiers and three classifiers representing molecular pathways involved in NAC response from our previous report^14^ and (2) seven MDA_MVAC classifiers and five TCGA 2017 classifiers comprising signature biomarkers for MIBC molecular subtyping for predicting the response to NAC^7^ and immune checkpoint inhibition^27^, respectively (Fig. 1b). Logistic regression modeling was then used to evaluate the prediction scores of each classifier for stratifying the AMC discovery cohort patients. The results showed that the data-driven and in-house NAC response-related classifiers outperformed traditional molecular subtyping classifiers (Fig. 1b).

Next, we designed a classifier reduction strategy to determine the optimal combination of diagnostic IHC markers (Materials and methods), which resulted in the inclusion of fewer than six optimal genes in each classifier (Supplementary Table 5). The classifier reduction process did not substantially affect the performance of most of the classifiers for the prediction of NAC response (difference in area under the receiver operating characteristic (ROC) curve (ΔAUC) <0.2), except TCGA 2017_Luminal and Neuronal ones (Supplementary Fig. 1d,e). Overall, these findings indicate that the machine learning analysis method can be applied to the prediction of NAC response, providing optimal candidate genes for IHC-based diagnostics using the MIBC cohort transcriptome dataset and various gene classifiers.

### Modeling for multicohort cross-validation of the transcriptome datasets

To validate the utility of the established machine learning model in external cohorts, we first used the MDA MVAC cohort (*n* = 23)^7^, which has a similar sample size to the AMC discovery cohort. Scale differences between the two datasets were normalized and the gene classifiers were refined by data preprocessing (Materials and methods). For cross-validation, three algorithms (logistic regression, decision tree and random forest) were applied to the power-scaled datasets, alternating between training and validation cohorts. The results indicated that both AMC NR and R classifiers effectively predicted the difference between NR and R groups in both cohorts when modeled using random forest or logistic regression (Fig. 1c and Supplementary Fig. 2a, b).

The scalability of the constructed model was evaluated by multicohort cross-validation. For this purpose, we extended the analysis to two independent cohorts: (1) an ‘MDA DDMVAC cohort’ comprising 38 patients from a phase II trial of DDMVAC and bevacizumab^28^ and (2) an ‘NAC metadata cohort’ consisting of 299 patients from seven institutions treated with various NAC regimens^8^. In addition to the differences in NAC regimens, the AMC cohort differed in that it included incomplete TURBT cases and used LCMD. Of the three cohorts, the NAC metadata cohort was similar to the AMC cohort because it used 1-mm punched tumor tissue, which was less likely to contain stromal and inflammatory cells. For the multicohort cross-validation, we analyzed transcriptome data for 39 patients (NR, *n* = 18; R, *n* = 21) for whom transcriptomic data were available from the validation cohort of 63 patients with MIBC (cT2-4aN0M0) used in the previous study^14^.

Following the preprocessing the four cohort datasets, we selected power scaler as the optimal scaling method by performing random forest-based premodeling (Materials and methods). To optimize the key variables in the 17 classifiers used in the previous analyses, we applied a classifier reduction process by (1) retaining the number of genes in the classifiers when the count was ≤10 and (2) selecting the top 10 key variables via random forest modeling for each dataset when the classifier gene count was >10. We also developed union classifiers by combining the top 10 genes from each classifier, which were obtained by using each cohort as a training set (Supplementary Table 6).

The results of 1,224 cross-validations (Materials and methods) indicated that logistic regression modeling using the NAC metadata cohort as the training set and the AMC cohort as the validation set achieved the best performance when applying both the top 10 and union MDA_MVAC_p63 classifiers. These models consistently yielded high AUC scores for stratifying NAC responders and nonresponders across all cohorts (Fig. 1d, e). In addition, models employing the top 10 MDA_MVAC_p53 or p53-like regulon classifiers also demonstrated solid discriminatory power across all cohorts. The union TCGA_Neuronal classifier showed strong performance in the AMC cohort but showed reduced discriminative performance in the MDA_MVAC and MDA_DDMVAC cohorts. When the random forest model was applied, the union MDA_MVAC_Luminal classifier effectively distinguished between NR and R patients in the AMC cohort and maintained predictive accuracy across the three external validation cohorts (Fig. 1e and Supplementary Fig. 2c). Notably, decision tree modeling using the NAC metadata cohort as the training set and the AMC cohort as the validation set showed strong performance when applying the union AMC data-driven classifier, particularly in both the NAC metadata and AMC cohorts (Fig. 1e and Supplementary Fig. 2d).

### Identification of biomarker candidates for predicting NAC response

To further identify IHC candidate proteins for predicting the response to NAC, the union AMC data-driven classifier was reduced to define a small number of key variables by applying NAC metadata and AMC cohort cross-validation modeling, which showed the strong performance for the training and validation sets. The logistic regression model was applied to reduce the classifier to 15 key variables (Supplementary Fig. 2e). The key classifiers included core genes such as *ITGAX/CD11C*, *AFAP1*, *KCTD14*, *RFX7*, *DPH2*, *SLC15A3*, *PPIL2*, *CARD16*, *FYB*, *GADD45B* and *DNMT3L* (Supplementary Table 7). We applied decision tree modeling to reduce the number of key variables in the classifier to 28 and further down to 6 while maintaining the predictive performance for NAC response (Supplementary Fig. 2e,f). The decision tree modeling identified *PCDHB9*, *HCFC1R1*, *NT5E*, *TNFAIP8*, *AFAP1* and *POU2F2* as key predictors of NAC response with the highest predictive importance (Fig. 1f and Supplementary Table 7). To further evaluate whether these predictive markers specifically reflect NAC resistance rather than overall tumor burden, we examined their associations with various clinicopathological parameters in the AMC validation cohort. Notably, lower expression levels of *AFAP1* and *POU2F2* were significantly correlated with high muscularis propria invasion (*P* = 0.017) (Supplementary Table 8). Furthermore, low *POU2F2* expression emerged as a robust prognostic indicator, significantly associated with shortened OS (*P* = 0.022), thereby underscoring its dual role in predicting both chemoresistance and poor clinical outcomes in MIBC. Taken together, these results confirm that the machine learning method developed for predicting the response to NAC is scalable and can be cross-validated using various multicohort transcriptome datasets. In addition, the classifier reduction process effectively identifies optimal key genes for further studies, such as IHC analysis or functional evaluation.

### Role of the stress response and cell adhesion genes for stratifying NAC responses

**Fig. 2:**
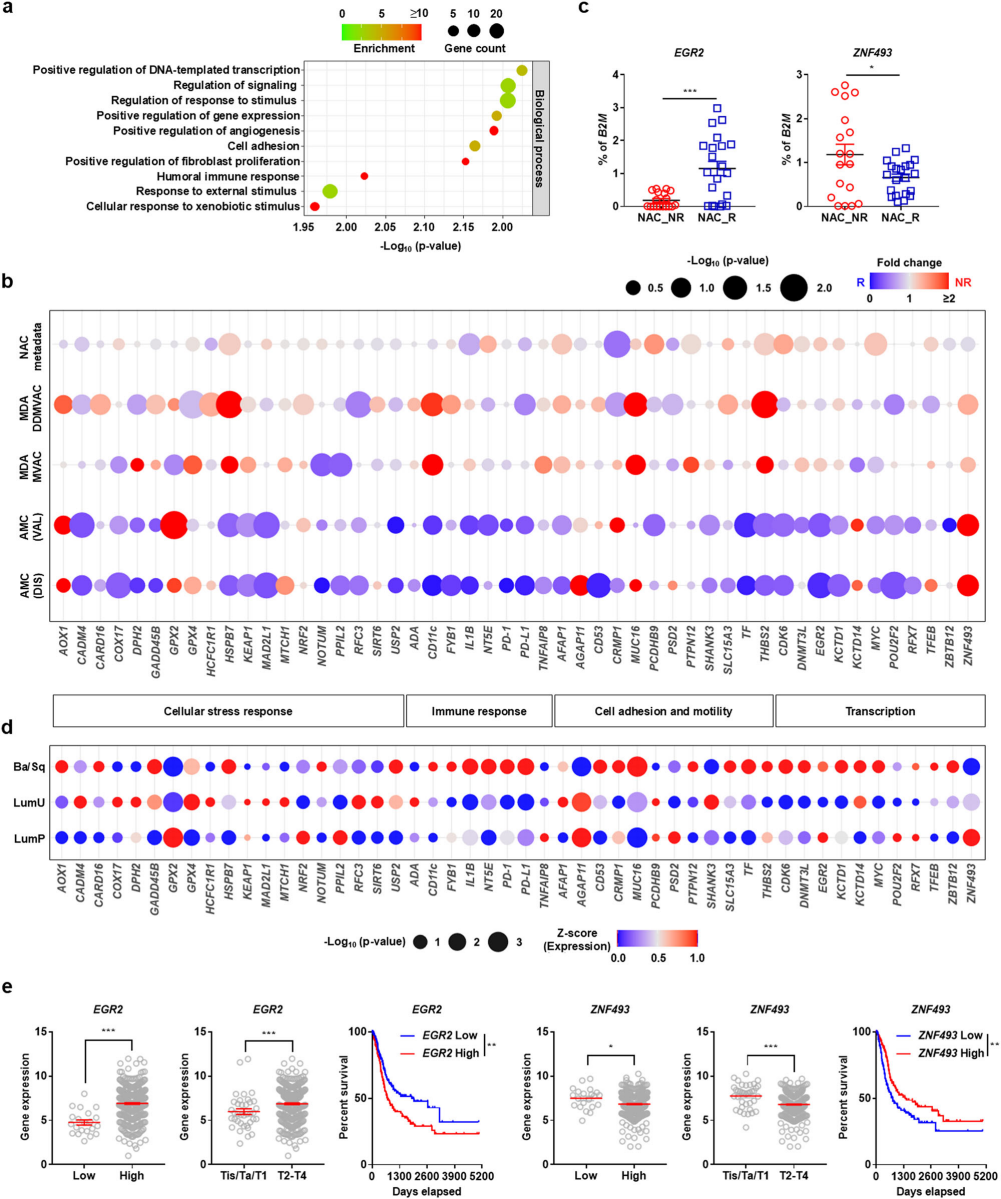
Validation of predictive biomarkers of NAC response in multiple cohorts. **a** GO analysis of the biological processes enriched in 50 putative biomarker genes assigned the highest weight in the classifiers from various modeling algorithms in the multicohort transcriptome analysis. **b** A bubble plot of RNA-sequencing results for the putative biomarker genes in the four independent cohorts used in the machine learning multicohort cross-validation analysis. In the bubble plot, the selected biomarkers are categorized by their biological functions including cellular stress response, immune response, cell adhesion and motility and transcriptional regulation. Within each functional category, genes are presented in alphabetical order. **c** qPCR analysis of *EGR2* and *ZNF493* expression in AMC cohort patients (NR, *n* = 18; R, *n* = 21). Gene expression is presented as percentage relative to human β2-microglobulin (*B2M*) expression. **d** A bubble plot displaying *z* scores of the expression of 50 putative biomarker genes in the AMC validation cohort following stratifying three identified consensus gene expression subtypes (Ba/Sq, *n* = 11; LumU, *n* = 10; LumP, *n* = 18). **e** *EGR2* and *ZNF493* expression levels in subgroups of BC patients according to tumor grade (left), pT category (middle) and OS (right). Gene expression raw data were obtained from UCSC’s Xena project (http://xena.ucsc.edu/). Quantitative results are shown as the mean ± s.e.m.; ^∗^*P* < 0.05, ^∗∗^*P* < 0.01 and ^∗∗∗^*P* < 0.001. Source data.

The biological characteristics of biomarkers associated with NAC response prediction were investigated by performing GO analysis of the 50 genes assigned the highest weight in the classifiers, which effectively stratified the NAC response in the multicohort validation model. These genes were enriched in categories related to the cellular response to external and xenobiotic stimuli, cell adhesion, angiogenesis, fibroblast proliferation and immune responses (Fig. 2a and Source data). RNA-sequencing of the AMC discovery and validation cohorts showed that genes involved in the cellular stress response (for example, *CADM4*, *COX17*, *GPX2*, *KEAP1*, *MAD2L1* and *POU2F2*), cell adhesion and motility (for example, *CD53*, *PCDHB9*, *TF* and *THBS2*) and transcription (*CDK6*, *EGR2*, *KCTD1* and *ZNF493*) were differentially expressed between NR and R group tumors (Fig. 2b and Source data). The expression of these putative biomarkers was confirmed by qPCR analysis using cDNA libraries derived from LCMD tumor specimens (Fig. 2c). qPCR expression analysis showed that the key predictors from logistic regression and decision tree modeling were differentially expressed according to NAC response (Supplementary Fig. 3). However, the expression patterns of these genes in the AMC cohorts differed from that in the three external cohort datasets except for *CADM4*, *COX17*, *POU2F2* and *ZNF493* (Fig. 2b).

To enhance the broader applicability of these findings, we employed the consensus MIBC classification method^6^, which stratified the AMC cohort into three distinct molecular subtypes: LumP (46.15%), LumU (25.64%) and Ba/Sq (28.21%). The absence of LumNS, stroma-rich and neuroendocrine-like (NE-like) subtypes in this cohort is probably attributable to the selective enrichment of BC cells via LCMD and the inherently low prevalence of the NE-like subtype^14^. Alignment of transcriptome data from the top 50 classifier genes with the consensus MIBC classification scheme revealed subtype-specific transcriptional patterns (Fig. 2d, Supplementary Fig. 4 and Supplementary Table 9). Ba/Sq tumors exhibited markedly elevated expression of immune-related markers (*IL1B* and *CD274/PD-L1*), while LumU tumors displayed high expression of genes implicated in cellular signaling pathways (*AGAP11* and *SHANK3*) (Supplementary Fig. 4). By contrast, LumP tumors were characterized by suppressed expression of the cell adhesion molecules (*TF* and *MUC16*). Among stress response-associated genes, *GPX4* was selectively upregulated in LumU tumors, whereas *GPX2* was predominantly elevated in LumP tumors.

To examine the clinical utility of the validated markers, we analyzed TCGA datasets of BC patients^29,30^ using the GEPIA^31^ and UCSC Xena project (http://xena.ucsc.edu/) web servers. A subset of the genes related to gene transcription (*CDK6*, *EGR2*, *MYC* and *ZNF493*), cell adhesion (*AFAP1*, *CRMP1*, *PSD2* and *THBS2*) and stress responses (*AOX1*, *GPX2*, *HSPB7* and *SIRT6*) was significantly related to the OS of BC patients from the TCGA datasets, and the altered expression of these biomarkers was dependent on tumor grade or pT category (Fig. 2e and Supplementary Fig. 5).

### Computational pathology for spatial protein expression analysis

We previously reported the use of computational pathology to analyze digitalized IHC slides for spatial protein expression by implementing a deep learning-based tumor and stroma classifier^14^. This classifier separates IHC slide images into cancer cell and stromal compartments, thereby enabling the quantification of the mean intensity of the chromogen DAB for each compartment. To evaluate the clinical importance of 33 genes identified in the transcriptome study for which antibodies were commercially available, we applied the digital pathology assay based on the tumor/stroma classifier to TMAs generated from pre-NAC TURBT specimens from the AMC NAC cohort (*n* = 55). In addition to the cohort of NAC-treated patients (cT2-4aN0M0) from our previous report^14^, we established an independent cohort of 63 patients with advanced MIBC (>cT2-4aN0M0) from our institute, which was designated as the PCT cohort. The TMAs analyzed by computational pathology included 36 patients from the PCT cohort.

**Fig. 3:**
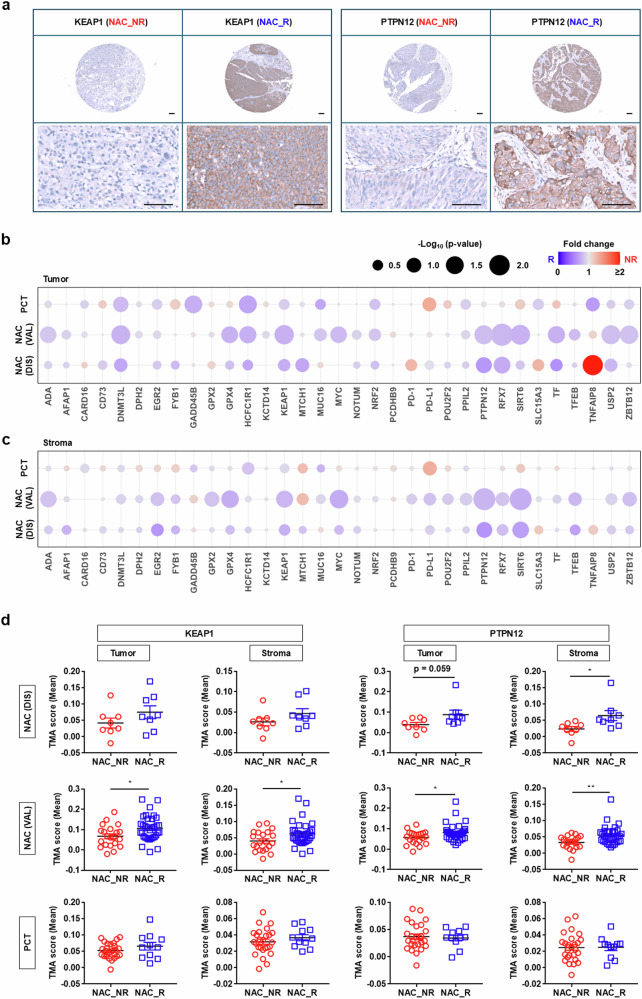
Computational pathology for spatial protein expression analysis. **a** Representative digitalized IHC images of the expression of KEAP1 and PTPN12 proteins in NAC NR and R group tumors (magnification: ×40 (top), ×200 (bottom); scale bar, 100 μm). **b**, **c** Bubble plots representing the digital pathology analyses of 33 proteins in tumorous epithelial cells (**b**) and the stroma compartment (**c**) from the AMC NAC discovery (DIS) and validation (VAL) cohorts (NR, *n* = 21; R, *n* = 34) or PCT cohort (NR, *n* = 25; R, *n* = 11). Gene symbols are presented in alphabetical order. **d** Expression levels of KEAP1 and PTPN12 proteins in the tumor (left) or stroma (right) compartment in the indicated AMC cohorts. Quantitative results are presented as dot plots of mean ± s.e.m. (^*^P < 0.05 and ^∗∗^*P* < 0.01, unpaired Student’s *t*-tests). Source data.

The results of computational pathology indicated that the expression levels of DNMT3L, KEAP1, PTPN12, RFX7, SIRT6, TNFAIP8, USP2 and ZBTB12 determined by IHC differed significantly between NAC R and NR group tumors of the AMC NAC cohort (Fig. 3a–d and Supplementary Fig. 6a). Consistent with transcript expression findings, the majority of proteins were enriched in R group tumors (Fig. 3b, d). For the AMC discovery cohort (*n* = 16), despite the small sample size, RFX7 and TNFAIP8 remained significantly upregulated in the R group and NR group tumors, respectively (Supplementary Fig. 6a). In addition, the expression of GPX4, HCFC1R1 and MYC was higher in R group tumors, although the statistical significance was marginal (Supplementary Fig. 6b). In the PCT cohort (*n* = 36), there was a statistically significant increase in HCFC1R1 and GADD45 protein expression in R group tumor cells, whereas DNMT3L was upregulated with marginal significance (Supplementary Fig. 6a, b).

Among the genes with notable protein expression differences between tumor compartments, KEAP1, MYC, PTPN12 and SIRT6 exhibited similar expression patterns in the stromal compartment of R tumors of the AMC NAC cohort (Fig. 3c, d and Source data). PTPN12 and SIRT6 proteins maintained significant expression differences in the AMC discovery cohort. In addition, ADA, GPX2 and GPX4 were upregulated in the stromal compartment of R tumors, although the significance of the differences was marginal.

Analysis of the spatial distribution of these proteins across distinct molecular subtypes, as defined by the consensus classification model, revealed subtype-specific expression patterns. In Ba/Sq subtype tumors, MUC16, TF and TFEB proteins were significantly upregulated, whereas NRF2 expression was notably downregulated in both tumor and stromal compartments (Supplementary Fig. 7a). In the LumU subtype, KCTD14 exhibited significant upregulation, while the expression of CARD16 and MYC proteins was also elevated, though with marginal statistical significance (Supplementary Fig. 7b). Tumors of the LumP subtype demonstrated a significant upregulation of HCFC1R1 expression (Supplementary Fig. 7b). In addition, within the stromal compartment of Ba/Sq tumors, several proteins including FYB1, NOTUM, SLC15A3 and USP2 were significantly upregulated, whereas DNMT3L and EGR2 showed an upward trend with marginal statistical significance (Supplementary Fig. 7c).

### Correlation of clinicopathologic features and biomarker expression profiles with NAC response and clinical outcomes

We added 33 proteins to the 41 proteins (Supplementary Fig. 6c, d) from our previous study^14^ and constructed a digital pathology dataset for analyzing tumor and stromal expression of 74 proteins using the AMC NAC (*n* = 55) and PCT (*n* = 36) cohorts (Supplementary Tables 2 and 3). Univariate analyses identified several clinicopathologic features and biomarker expression levels significantly associated with the response to NAC (Supplementary Table 10). Consistent with previous findings^14^, incomplete resection of the tumor during TURBT (*P* = 0.022), elevated GLS protein levels (*P* = 0.029) and low CD11c expression (*P* = 0.013) in tumor cells were associated with NAC resistance (Table 1). In addition, low expression levels of DNMT3L (*P* = 0.024), RFX7 (*P* = 0.032), SIRT6 (*P* = 0.024), USP2 (*P* = 0.024) and ZBTB12 (*P* = 0.022) within tumor cells were significantly correlated with NAC resistance. In the stromal compartment, the expression levels of ten biomarkers, including KEAP1, were similarly associated with the response to NAC (Table 1).

**Table 1:** Correlation of clinicopathologic features with biomarker expression profiles.

Variable		Univariate analysis					Multivariate analysis		
		Response group	Mean ± s.e.m.	OR	95% Confidence interval	*P* value	OR	95% Confidence interval	*P* value
**Age (years)**		**NR**	**64.50** ± **11.21**			**0.831**			
		**R**	**65.11** ± **11.03**						
**Gender (male)**				**1.789**	**0.416–7.684**	**0.43**			
**Pre-NAC tumor**	**Location (bladder)**			**1.440**	**0.086–24.120**	**0.799**			
	**Size (≥3** **cm)**			**2.015**	**0.647–6.278**	**0.223**			
	**Multiplicity (multiple)**			**1.026**	**0.372–2.833**	**0.96**			
	**Resection (incomplete)**			**3.500**	**1.172–10.449**	**0.022**			
	**Stage**					**0.062**			
	**Grade (high)**					–			
	**Carcinoma in situ**			**0.577**	**0.607–4.953**	**0.303**			
	**Lymphovascular invasion**			**0.440**	**0.143–1.354**	**0.148**			
**Tumor**	**CD11c**			**4.182**	**1.314–13.304**	**0.013**	**7.771**	**1.475–40.938**	**0.016**
	**DNMT3L**			**3.667**	**1.163–11.557**	**0.024**	**8.386**	**1.443–48.736**	**0.018**
	**GLS**			**0.280**	**0.087–0.898**	**0.029**	**0.064**	**0.009–0.472**	**0.007**
	**RFX7**			**3.398**	**1.090–10.588**	**0.032**			
	**SIRT6**			**3.667**	**1.163–11.557**	**0.024**			
	**USP2**			**3.667**	**1.163–11.557**	**0.024**			
	**ZBTB12**			**4.737**	**1.171–19.155**	**0.022**			
**Stroma**	**ADA**			**4.737**	**1.171–19.155**	**0.022**			
	**CD11c**			**3.571**	**1.111–11.477**	**0.029**	**12.927**	**1.167–21.355**	**0.028**
	**GPX2**			**3.667**	**1.163–11.557**	**0.024**			
	**GPX4**			**3.667**	**1.163–11.557**	**0.024**			
	**KEAP1**			**5.273**	**1.468–18.932**	**0.008**	**41.627**	**2.972–582.998**	**0.006**
	**MYC**			**3.167**	**0.989–10.141**	**0.048**	**34.719**	**1.517–794.381**	**0.026**
	**PD-1**			**3.600**	**1.074–12.062**	**0.033**			
	**PTPN12**			**3.883**	**1.210–12.467**	**0.020**			
	**SIRT6**			**6.000**	**1.807–19.925**	**0.002**			
	**SOX2**			**0.236**	**0.069–0.806**	**0.017**	**0.022**	**0.001–0.458**	**0.014**

Univariate and multivariate analyses of clinicopathological factors and expression levels of 33 candidate proteins for predicting NAC response identified by machine learning analysis. Details of the univariate analysis of protein expression in tumor and stromal compartments are provided in Supplementary Table 10.

Multivariate logistic regression analysis identified high GLS expression (*P* = 0.007) and low levels of CD11c (*P* = 0.016) or DNMT3L (*P* = 0.018) in tumor cells as independent predictors of NAC resistance (Table 1). The increase in GLS expression was associated with a decreased pathologic response, whereas upregulation of CD11c and DNMT3L corresponded to 7.771-fold and 8.386-fold increases in the pathologic response, respectively. Regarding stromal biomarkers, high SOX2 expression (*P* = 0.014) and low levels of CD11c (*P* = 0.016), MYC (*P* = 0.026) or KEAP1 (*P* = 0.006) were identified as independent prognostic factors; SOX2 was associated with a decrease in the pathologic response, whereas CD11c, MYC and KEAP1 were associated with 12.927-fold, 34.719-fold and 41.627-fold increases in the pathologic response, respectively (Table 1).

### Machine learning-based analysis of the digital pathology datasets for the predictive IHC panel

Next, to identify the optimal number of protein combinations for predicting NAC responsiveness using clinically applicable IHC staining, the digital pathology datasets from the AMC NAC and PCT cohorts (Supplementary Tables 2 and 3) were analyzed using deep learning techniques similar to those used in transcriptome cross-validation modeling. Owing to the lack of external cohorts with IHC digital pathology results, we used the AMC NAC and PCT cohorts for cross-validation of the digital pathology datasets.

**Fig. 4:**
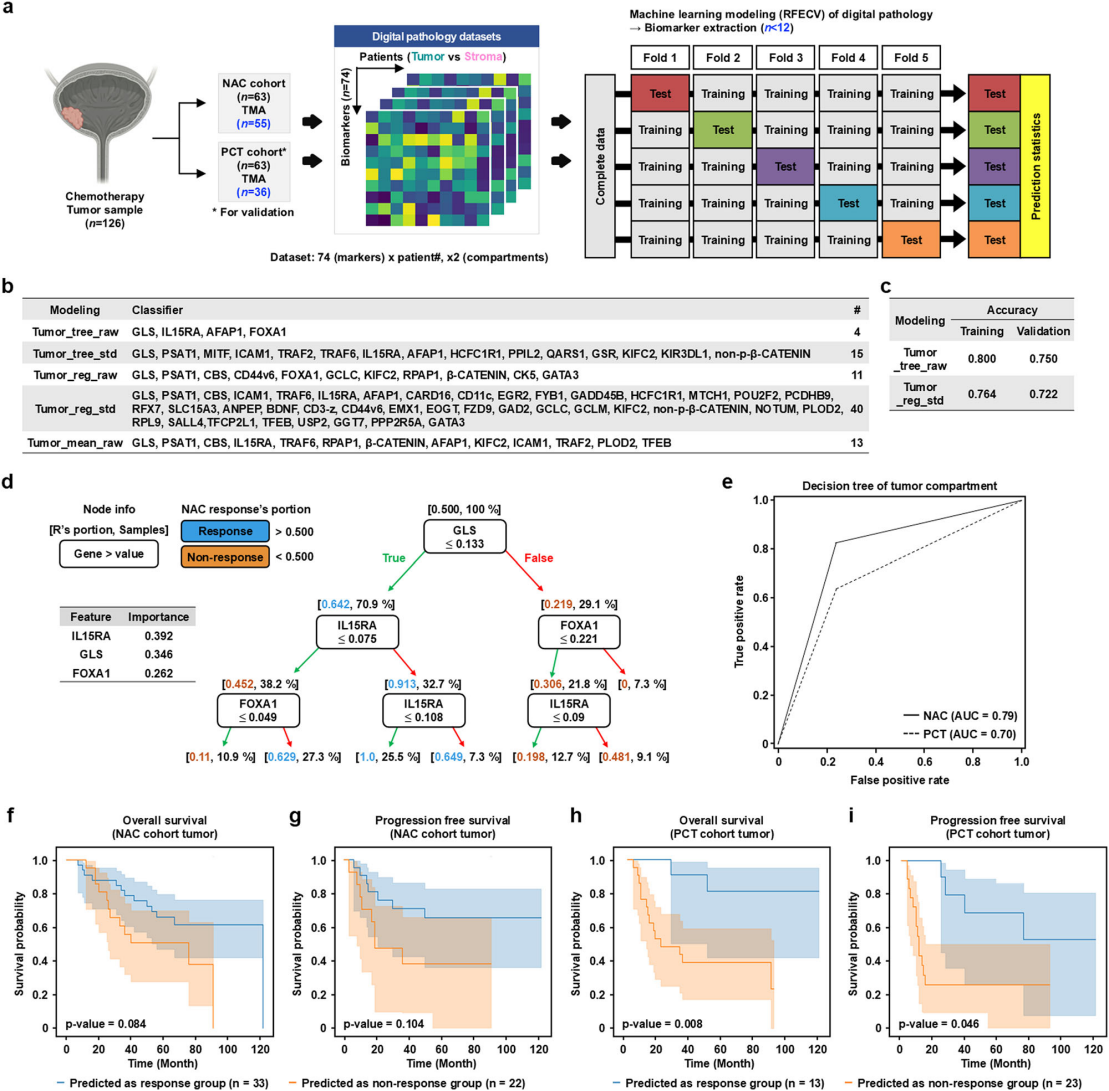
Digital pathology modeling with machine learning for developing an IHC panel. **a** A schematic overview of the variable selection and RFECV modeling of the digital pathology datasets from AMC NAC and PCT cohorts for developing an optimized IHC panel to predict the response to NAC. The expression levels of 74 proteins in AMC NAC (*n* = 55) and PCT (*n* = 36) cohort patients in the tumor and stroma and clinical annotations are presented in Supplementary Tables 2 and 3. **b** A summary of the modeling methods, data types and list or number of proteins corresponding to the biomarkers with the highest weight in each modeling algorithm that effectively distinguished between NAC NR and R groups based on the expression in the tumor compartment. **c** The accuracy of the model performance for NAC response prediction in the cross-validation between the AMC NAC (training) and PCT (validation) cohorts. **d** A decision tree model of the tumor compartment. The key feature proteins for a decision are presented in outcome branches with different colors (true, green; false, red). Terminal nodes indicate the predicted class of NAC response (R, blue; NR, orange) and the frequency of samples. The importance of key features is presented with the decision tree. **e** ROC curve of the predictive performance of the tumor compartment decision tree model in the AMC NAC and PCT cohorts. **f**–**i** Survival outcomes based on the computational pathology prediction model: Kaplan–Meier curves of OS (**f** and **h**) and PFS (**g** and **i**) stratified by predicted NAC response or no-response in the AMC NAC (**f** and **g**) and PCT (**h** and **i**) cohorts. Predictions were generated using the decision tree models of the computational pathology datasets of tumor compartments.

To address the limitations posed by the small number of measured biomarkers (*n* = 74), limited patient samples (55 NAC and 36 PCT cases) and different clinical characteristics between the training and validation groups, we used a data-driven classifier from the RFECV method (Materials and methods). The recursive feature elimination part of this method measures the performance of drop-out cases relative to that of the included cases for every variable to focus on the more impactive features of small size. To overcome the limitations of insufficient dataset size for training and validating, the method recruited the *k*-fold cross-validation, which divides a single dataset into *k* sets and trains *k* −1 sets, validating the remaining single set *k* times sequentially. The relative performance of a single variable was evaluated an average of *k* times in different sets (Fig. 4a). The variables selected by the RFECV were used to develop lighter models of two to five variables of the forward–backward-stepwise logistic regression and the decision tree of three-max-depth on the original raw data and on the standardized data.

For each tumor and stroma compartment, we identified protein groups capable of distinguishing between NAC NR and R groups for each data type and modeling method (Fig. 4b and Supplementary Fig. 8a). Among the finalized models, the decision tree model with raw data of tumor compartments (Tumor_tree_raw) was the most effective in predicting the response to NAC by combining four proteins (GLS, IL15RA, AFAP1 and FOXA1) (Fig. 4c). Detailed results and the key genes involved in the best decision tree models for tumor and stromal compartments are shown in Fig. 4d (tumor expression) and Supplementary Fig. 8b (stroma expression).

To validate the clinical utility of the deep learning-based modeling method for computational pathology, we compared the performance of NAC and PCT cohorts. The decision tree model of the tumor compartment (Tumor_tree_raw) was a strong predictor of NAC response (Fig. 4e) and the distribution of patients with different NAC responses was distinctly separated in the AMC NAC cohort. This finding was validated in the PCT cohort (Supplementary Fig. 8c). However, for stromal expression-based modeling, the distinction of NAC responses was not as clear, particularly in the PCT cohort (Supplementary Fig. 8d–f). This outcome may be attributed to the variable selection and the low frequency of highly informative variables in the stromal compartment expression datasets from both AMC cohorts. The present study provides a deep learning-based modeling approach that integrates high-throughput automated digital pathology analysis of large-scale MIBC patient samples, leading to the optimal small number of protein combinations for IHC staining of tumor and stromal compartments to predict the response to NAC.

### Survival outcomes of computational pathology-based prediction modeling

To assess the prognostic relevance of NAC response predictions derived from the computational pathology model, we analyzed survival outcomes in both the NAC and PCT cohorts (Materials and methods). In the NAC cohort, patients predicted to be NAC responders based on tumor biomarker expression exhibited prolonged OS (*P* = 0.084) and PFS (*P* = 0.104), though with marginal statistical significance (Fig. 4f, g). Notably, in the PCT cohort, NAC responders demonstrated significantly improved OS (*P* = 0.008) and PFS (*P* = 0.046) compared with nonresponders, as determined by the tumor-based model (Fig. 4h, i).

When predictions were based on stromal biomarker expression, NAC responders in the NAC cohort exhibited a trend toward prolonged OS (*P* = 0.063) and significantly longer PFS (*P* = 0.015) compared with nonresponders (Supplementary Fig. 9). However, no significant survival differences were observed in the PCT cohort using the stromal-based model. Collectively, these findings highlight a strong association between computational pathology-based NAC response predictions and survival outcomes in patients with MIBC, underscoring their potential clinical utility for treatment stratification.

### Mechanistic validation of KEAP1–NRF2 pathway for stratifying NAC response in MIBCs

**Fig. 5:**
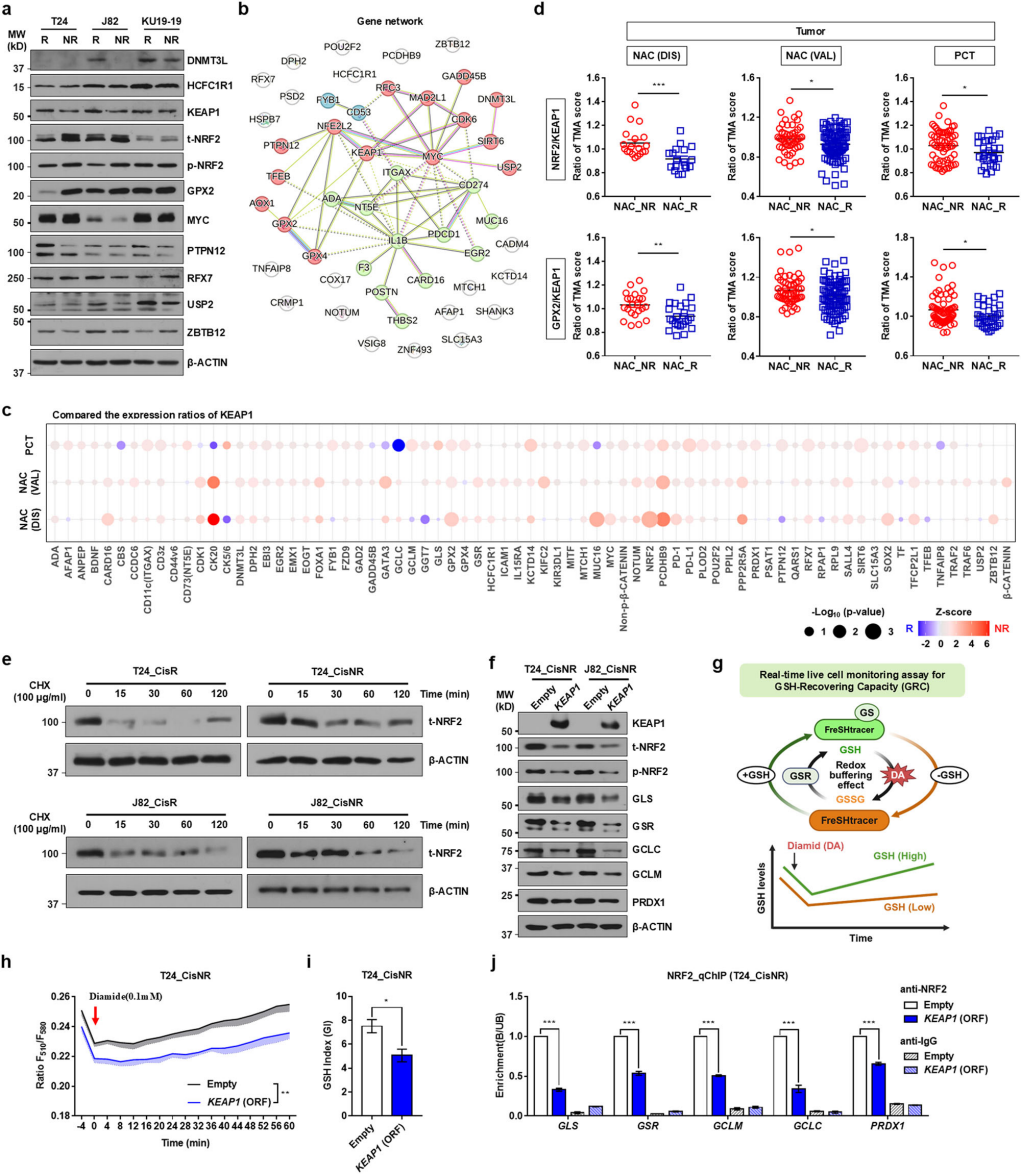
KEAP1–NRF2 axis regulates NRF2 stability, GSH metabolism and cisplatin resistance in human MIBC cells. **a** Western blot analysis of clinically relevant biomarker proteins in cisplatin responsive (Cis_R) and nonresponsive (Cis_NR) MIBC cell lines. β-Actin was used as a loading control. Molecular weight (MW) marker sizes (kD) are shown on the left. **b** A gene interaction network of biomarkers characterizing the response to NAC validated using machine learning analysis. The external stress and immune response gene clusters are indicated in red and green, respectively. **c**, **d** Protein expression ratios relative to KEAP1 determined by computational pathology of the tumor compartment: a bubble plot displaying *z* scores and the statistical significance of protein expression ratios relative to KEAP1 in NR and R groups from the AMC NAC and PCT cohorts (**c**) and dot plots showing NRF2–KEAP1 (top) and GPX2–KEAP1 (bottom) protein expression ratios in the AMC cohorts (**d**). The ratios were calculated using values from three independent TMA cores per patient. **e** NRF2 protein stability was evaluated by CHX chase assay in Cis_R and Cis_NR T24 (left) and J82 (right) cells. **f** *KEAP1* was ectopically expressed in Cis_NR T24 and J82 cells, followed by immunoblot analysis of GSH metabolism-associated genes. **g** A schematic of real-time live-cell GSH GRC assay using FreSHtracer after oxidative challenge with 0.1 mM diamide (red arrow). The glutathione index (GI) was derived from both baseline *F*_510_/*F*_580_ fluorescence ratio and GSH recovery slope. **h**, **i** Fluorescence ratio-based fluorescence plots (**h**) and GI quantification (**i**) in *KEAP1*-overexpressing Cis_NR T24 cells (*n* = 4). **j** ChIP–qPCR analysis of NRF2 binding to the promoter regions of GSH-related target genes in *KEAP1*-overexpressing Cis_NR T24 cells (*n* = 4). Fold enrichment is shown relative to empty vector controls. Quantitative data are expressed as the mean ± s.e.m.; ^∗^*P* < 0.05, ^∗∗^*P* < 0.01, ^∗∗∗^*P* < 0.001, unpaired Student’s *t*-test (**d** and **i**) and two-way ANOVA (**h** and **j**) with Bonferroni post hoc test. Source data.

To explore potential therapeutic strategies based on biomarkers identified through machine learning analysis, we first assessed the differential expression of clinically relevant biomarker proteins in various human MIBC cell lines with distinct responses to cisplatin (Cis_R) and nonresponse (Cis_NR) behaviors. Consistent with clinical sample data, the expression of DNMT3L, GPX2, HCFC1R1, KEAP1, MYC, PTPN12, RFX7, USP2 and ZBTB12 was higher in Cis_R J82 MIBC cells than in Cis_NR cell lines (Fig. 5a and Supplementary Fig. 10a–c). The reduced expression of KEAP1 in Cis_NR T24 and J82 cells was confirmed by immunofluorescence staining (Supplementary Fig. 10d). In line with the repression of KEAP1, total and phosphorylated NRF2 proteins were upregulated in Cis_NR J82 and T24 cells. Notably, their nuclear localization, an indicative of NRF2 pathway activation, was markedly increased in these chemoresistant MIBC cells (Supplementary Fig. 10e, f). The ADA and GPX4 proteins were upregulated in Cis_R T24 cells, whereas GADD45B and TNFAIP8 showed increased expression in Cis_R KU19-19 MIBC cells (Supplementary Fig. 10g).

Next, we examined the association between these biomarkers and identified two key gene networks: (1) an external stress response network involving KEAP1 and MYC and (2) an immune response gene cluster including IL1B and CD274/PD-L1 (Fig. 5b). To validate the clinical relevance of these findings, we compared the expression ratios of KEAP1 or MYC proteins and their target proteins between NR and R group patients. The ratios of several proteins including NRF2, GPX2, CK20, MUC16, PCDHB9 and PPP2R5A to the KEAP1 protein were significantly elevated in the NR tumor compartment of the AMC NAC cohort (Fig. 5c, d and Source data). In particular, analysis of the ratios to the MYC protein identified a substantial number of proteins with increased ratio values in the NR tumor compartment of the AMC NAC validation cohort (Supplementary Fig. 10h, i and Source data), suggesting that KEAP1 and MYC could play a role in regulating the biomarkers predictive of NAC response.

Based on recent evidence suggesting that NRF2 activation promotes an immune-excluded microenvironment^32^, we sought to determine whether NRF2 pathway can be associated with suppressed immune infiltration in our LCMD-derived AMC cohort. Correlation analysis using the RNA-sequencing dataset revealed a significant inverse relationship between *NRF2*/*NFE2L2* expression and key immune-response-related genes, including *CXCL9*, *CXCL10*, *CD11c*, *CD53* and *CD274*/*PD-L1* (Supplementary Fig. 11a, b). Intriguingly, this negative correlation was more pronounced in responders (R group) compared with nonresponders (NR group), particularly for *CD11c*, *CXCL9* and *CD274*, suggesting that NRF2-mediated immune evasion may be a critical determinant of therapeutic sensitivity. Furthermore, consistent with its canonical role in redox homeostasis, *NRF2* expression showed strong positive correlations with GSH dynamics-related genes, such as *GLS*, *GSR*, *GCLM*, *GCLC* and *PRDX1* (Supplementary Fig. 11a, c). Collectively, these data indicate that heightened NRF2 activity in MIBC not only orchestrates antioxidant defense via GSH metabolic reprogramming but also potentially contributes to a ‘cold’ tumor microenvironment by suppressing intrinsic pro-inflammatory signaling.

### Functional role of KEAP1 in regulating GSH dynamics in chemoresistant MIBC cells

We previously reported that increased GSH dynamics, driven by coordinated upregulation of GLS, GSR, and GCLM, promotes cell proliferation, stemness, and invasiveness in MIBC cells^14^. In this context, the upregulation of KEAP1 in cisplatin-sensitive tumors, which enhances NRF2 ubiquitination and proteasomal degradation, suggests that modulation of the KEAP1–NRF2 cascade may improve therapeutic responsiveness. Indeed, cycloheximide (CHX) chase assays revealed reduced NRF2 protein stability in both Cis_R J82 and T24 cells (Fig. 5e), consistent with enhanced KEAP1-mediated degradation. Next, to mechanistically validate this hypothesis, we ectopically expressed *KEAP1* in the Cis_NR T24 and J82 MIBC cell lines and examined the expression changes in genes associated with GSH biosynthesis (*GCLM* and *GCLC*), precursor metabolism (*GLS*), and utilization (*GSR* and *PRDX1*). *KEAP1* overexpression led to transcriptional and protein-level downregulation of most GSH-associated genes in both cell lines (Fig. 5f and Supplementary Fig. 12a).

To assess the functional impact of KEAP1 on antioxidant capacity in MIBC cells, we employed FreSHtracer, a reversible fluorescent probe enabling real-time, nondestructive imaging of intracellular GSH levels^33,34^. Using this approach, we real-time monitored and quantified GSH redox dynamics by measuring GRC following oxidative challenge with 100 μM diamide over a 1-h time course (Fig. 5g). Consistent with the gene expression results, *KEAP1*-overexpressing Cis_NR cells exhibited a significant reduction in GSH dynamics, as indicated by elevated baseline fluorescence ratio (*F*_510_/*F*_580_; total GSH) and increased slopes indicative of accelerated GSH turnover during redox recovery (Fig. 5h, i and Supplementary Fig. 12b–d).

Importantly, *KEAP1* overexpression decreased NRF2 protein stability (Supplementary Fig. 12e), leading to reduced levels of total and phosphorylated NRF2, along with a marked decrease in its nuclear localization in Cis_NR cells (Fig. 5f and Supplementary Fig. 12f,g), confirming effective suppression of NRF2 signaling. ChIP assays further demonstrated decreased NRF2 occupancy at promoter regions of the GSH-regulating genes in *KEAP1*-overexpressing cells (Fig. 5j and Supplementary Fig. 12h). Together, these findings highlight KEAP1 as a key upstream modulator of GSH dynamics through NRF2-dependent transcriptional regulation.

### Role of the KEAP1–NRF2 axis in regulating proliferation and stemness in MIBC cells

**Fig. 6:**
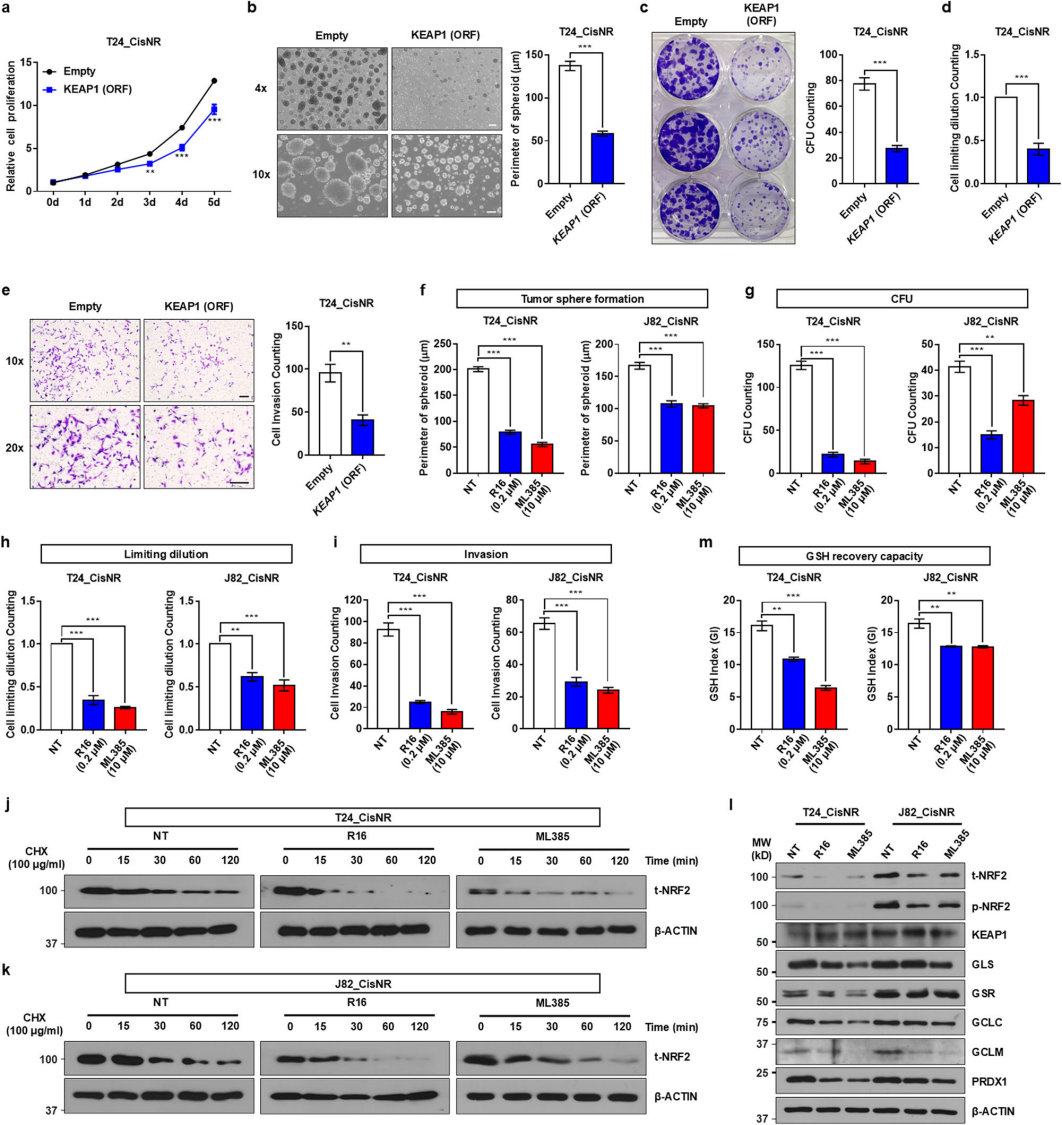
The KEAP1–NRF2 axis regulates stemness properties in cisplatin-resistant MIBC cells. **a**–**e** Functional assays assessing proliferation (**a**; *n* = 4) and stemness features (**b**–**e**) based on tumor sphere formation (**b**; *n* = 45), colony forming unit (CFU) activity (**c**; *n* = 3), clonogenicity by limiting dilution (**d**; *n* = 3) and Matrigel invasion (**e**; *n* = 4) in *KEAP1*-overexpressing Cis_NR T24 cells. Representative tumor spheres are shown at ×40 (upper) and ×100 (lower) magnification (scale bars, 200 μm); invasion assays are shown at ×100 (upper) and ×200 (lower) magnification (scale bars, 100 μm). **f**–**i** Tumor sphere (**f**; *n* = 45), CFU capacity (**g**; *n* = 3), clonogenic limiting dilution (**h**; *n* = 3) and invasion (**i**; *n* = 4) assays following treatment of Cis_NR T24 (left) and J82 (right) cells with KEAP1–NRF2 inhibitors ML385 or R16. Representative images are provided in Supplementary Fig. 14a–c. **j**, **k** NRF2 protein stability after KEAP1–NRF2 pathway inhibition was evaluated by CHX chase assay in Cis_NR T24 (**j**) and J82 (**k**) cells. **l** Immunoblot analysis of total and phosphorylated NRF2 proteins and GSH-related markers following treatment with each KEAP1–NRF2 inhibitor. **m** Fluorescent ratio-based quantification of the GSH index in KEAP1–NRF2 inhibitor-treated Cis_NR T24 (left) and J82 (right) cells (*n* = 3). Data are presented as mean ± s.e.m. *P* < 0.05, **P < 0.01 and ***P < 0.001. Statistical analyses were performed using unpaired two-tailed Student’s *t*-tests (**b**–**e**) and one-way ANOVA (**f**–**m**) with Bonferroni post hoc test. Cells in the NT (non-treated) group were used as a control.

Previous studies have shown that enhanced GSH dynamics promote proliferative capacity and stem-like properties, which are critical for clinical behaviors of BCs^1,2,14^. Consistent with this, ectopic expression of *KEAP1* significantly suppressed the proliferation of the Cis_NR T24 and J82 cells (Fig. 6a and Supplementary Fig. 13a). When cultured under anchorage-independent conditions, *KEAP1*-overexpressing Cis_NR cells exhibited markedly reduced tumor sphere-forming ability over a 5-day period compared with vector control cells (Fig. 6b and Supplementary Fig. 13b). Moreover, both the clonogenic potential and invasive capacity of *KEAP1*-expressing cells were diminished, as confirmed by limiting dilution and transwell invasion assays, respectively (Fig. 6c–e and Supplementary Fig. 13c–e).

To further validate the functional role of the KEAP1–NRF2 pathway, the Cis_NR T24 and J82 cells were treated with two distinct NRF2 pathway inhibitors, ML385 and R16 (refs. ^18,19^). Pharmacological inhibition of the KEAP1–NRF2 axis markedly attenuated tumor sphere formation (Fig. 6f and Supplementary Fig. 14a), clonogenic growth (Fig. 6g, h and Supplementary Fig. 14b), and invasive capacities (Fig. 6i and Supplementary Fig. 14c), supporting its critical role in maintaining the stemness phenotype of chemoresistant MIBC cells. Mechanistically, treatment with ML385 or R16 markedly decreased the expression and protein stability of NRF2 (Fig. 6j, k), accompanied by the downregulation of the NRF2-regulated genes involved in GSH metabolism (Fig. 6l and Supplementary Fig. 14d). Accordingly, ML385- or R16-treated Cis_NR T24 and J82 cells showed a substantial reduction in GSH index profiles, as determined by real-time GRC live cell imaging assay (Fig. 6m and Supplementary Fig. 14e). These findings collectively indicate that KEAP1–NRF2 signaling governs GSH dynamics and regulates cellular proliferation, stem-like properties and invasiveness in human MIBC cells, and provide mechanistic support for targeting the KEAP1–NRF2 pathway as a strategy to overcome chemoresistance in MIBC.

### Therapeutic targeting of the KEAP1–NRF2 axis resensitizes chemoresistant MIBC cells

**Fig. 7:**
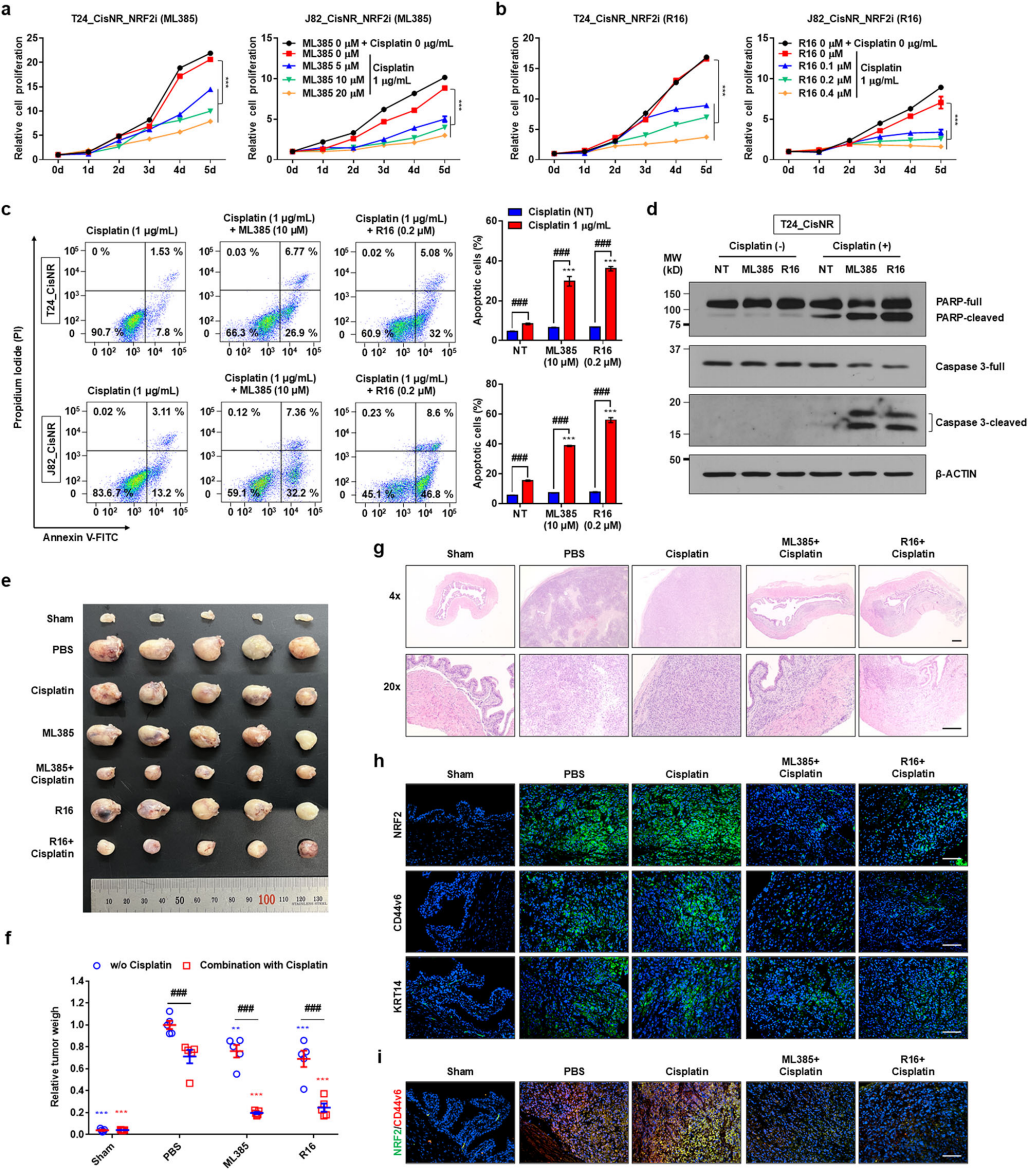
Pharmacological inhibition of the KEAP1–NRF2 pathway resensitizes cisplatin-resistant MIBC cells to chemotherapy. **a**, **b** Growth curves of T24 and J82 Cis_NR MIBC cell lines treated with the indicated doses of the NRF2–KEAP1 inhibitors ML385 (**a**) and R16 (**b**) alone or in combination with 1 μg/ml cisplatin (*n* = 6). **c**, **d** Analysis of apoptosis by flow cytometry with Annexin-V/PI staining (**c**; *n* = 4) and immunoblotting of cleaved caspase-3 and PARP (**d**) in T24 and J82 Cis_NR MIBC cells treated with the indicated NRF2–KEAP1 pathway inhibitors alone or in combination with 1 μg/ml cisplatin. **e**–**h** Evaluation of therapeutic efficacy in an orthotopic bladder cancer xenograft model treated with cisplatin (1 mg/kg), ML385 (30 mg/kg), R16 (55 mg/kg) or their combination. Bladder tumors were excised and visualized (**e**) and tumor weights quantified (**f**) from two independent experiments (five mice per group). **g** Representative hematoxylin and eosin (H&E) staining of bladder tissues from each treatment group at ×40 (top; scale bars, 200 μm) and ×200 (bottom; scale bars, 100 μm) magnification. **h**, **i** Immunofluorescence analysis of xenograft tumors for KEAP1, NRF2 and the cancer stem cell marker CD44v6 or KRT14 (**h**) and co-localization of NRF2 (green) and CD44v6 (red) proteins (**i**) in residual tumor tissues. Nuclei were counterstained with DAPI (blue). Images were acquired at ×200 magnification (scale bars, 100 μm). Data are presented as mean ± s.e.m. Statistical significance was determined by two-way ANOVA with Bonferroni post hoc test. *P* < 0.05, *P* < 0.01, *P < 0.001 versus vehicle control; ^#^*P* < 0.05, ^##^*P* < 0.01, ^###^*P* < 0.001 for combination versus monotherapy. Source data.

To explore the therapeutic potential of the NRF2–KEAP1 pathway for modulating the response to cisplatin-based neoadjuvant chemotherapy in human MIBCs, cells were treated with varying doses of cisplatin in the presence or absence of two types of NRF2–KEAP1 inhibitors (ML385 and R16). In J82 and T24 Cis_R MIBC cell lines, the combination of cisplatin with each NRF2–KEAP1 inhibitor markedly suppressed cell growth at a suboptimal cisplatin concentration (1 μg/ml) (Fig. 7a, b), whereas the NRF2–KEAP1 inhibitors alone had a minimal impact on cell growth (Supplementary Fig. 15a, b). The combination therapy synergistically inhibited the growth of Cis_NR MIBC cells in a dose-dependent manner (Supplementary Fig. 15c, d). Notably, enforced expression of *KEAP1* in Cis_NR T24 and J82 cells sensitized them to cisplatin in a dose-dependent manner, further confirming the critical role of KEAP1 in mediating chemosensitivity (Supplementary Fig. 15e, f). Consistent with these results, disruption of the NRF2–KEAP1 pathway induced apoptotic cell death in response to cisplatin treatment (Fig. 7c), activating effector caspase-3 and promoting cleavage of poly-(ADP-ribose) polymerase (PARP) in Cis_NR MIBC cells (Fig. 7d and Supplementary Fig. 15g). Suboptimal cisplatin or NRF2–KEAP1 modulators alone had no effect on caspase-3 or PARP cleavage or apoptosis.

To confirm the in vivo relevance of these findings, we examined the effect of NRF2-specific inhibitor (ML385 or R16) alone or in combination with cisplatin in an orthotopic xenograft BC model by implanting of 1 × 10^6^ Cis_NR T24 cells into the outer layer of the bladder of NSG immunodeficient mice^1,2,14^. After 2 weeks, animals were randomized to receive PBS vehicle, cisplatin (1 mg/kg), ML385 (30 mg/kg), R16 (55 mg/kg) or combination of cisplatin and each NRF2 inhibitor over ten intraperitoneal injections at 3-day intervals (Supplementary Fig. 16a). Monotherapy with cisplatin, ML385 or R16 induced only modest tumor inhibition (28.81% ± 12.31%, 23.93% ± 11.34% and 30.9% ± 14.88%, respectively), whereas combination therapy with cisplatin and either ML385 or R16 led to a striking reduction in tumor burden (80.29% ± 1.88% and 75.44% ± 7.4%, respectively) at 6 weeks after tumor engraftment (Fig. 7e, f). Histological analyses confirmed robust tumor inhibition in the combination treatment groups, which was not evident in monotherapy or control-treated mice (Fig. 7g and Supplementary Fig. 16b).

We further examined the expression of KEAP1–NRF2-mediated GSH metabolism-related proteins and stemness markers in xenograft tissues. Immunofluorescence revealed that cisplatin monotherapy led to upregulation of NRF2 and its downstream targets GLS, GSR and GCLM, whereas co-treatment with NRF2-specific inhibitors (ML385 or R16) effectively suppressed their expression (Fig. 7h and Supplementary Fig. 16c). Similarly, expression of bladder cancer stem cell markers CD44v6 and KRT14 was markedly suppressed in tumors from animals receiving the combination regimens (Fig. 7h). Consistent with our clinical findings, immunofluorescence analysis revealed that cisplatin monotherapy markedly induced the co-expression of NRF2 and GLS, the primary driver of GSH dynamics, in residual tumor tissues. However, this induction was effectively abrogated by co-treatment with NRF2-specific inhibitors, ML385 or R16 (Supplementary Fig. 16d). In addition, co-expression of CD44v6 and NRF2 was frequently observed in vehicle or cisplatin-treated tumors; however, it was substantially diminished following combined treatment with cisplatin and either ML365 or R16 (Fig. 7i). Together, these findings demonstrate that therapeutic targeting of the KEAP1–NRF2 pathway enhances cisplatin sensitivity in MIBCs, underscoring the translational potential of the machine learning model integrating transcriptome and digital pathology datasets as a framework for guiding rational combination therapies to overcome chemoresistance in MIBCs.

## Discussion

This study highlights the potential of machine learning-based models and digital pathology for predicting the response to NAC in MIBC. The integration of multimodal omics datasets with advanced computational methods provided important information for biomarker discovery, for the development of predictive algorithms for clinical application. Systematic analysis of transcriptome datasets integrated with digital pathology via machine learning algorithms led to the identification of candidate genes and proteins with potential predictive value, providing a scalable and robust approach to stratifying patients according to the response to NAC. Furthermore, mechanistic validation of the KEAP1–NRF2 pathway demonstrated its functional role in redox-driven chemoresistance and revealed a therapeutic opportunity to re-sensitize cisplatin-resistant MIBC.

One of the most critical challenges in the treatment of MIBC is its molecular and clinical heterogeneity, which contributes to variable patient outcomes following standard treatments, such as cisplatin-based NAC. Certain molecular subtypes, such as p53-like and basal tumors, exhibit differential responses to NAC^7,8^. However, the classification into subtypes based on the transcriptome alone is not sufficient for reliably predicting patient outcomes^6^ and conflicting outcomes have been reported^9,10^. These limitations are overcome through the application of machine learning techniques, which were used to refine gene classifiers and to perform computational pathology analysis, thereby enhancing the clinical relevance and utility for predicting NAC responses. Combined with the classifiers comprising signature biomarkers for MIBC molecular subtyping and therapeutic response, the data-driven classifiers representing the molecular features of datasets provide a more nuanced method for capturing the complex biology of the NAC response.

The findings of this study highlight the importance of genes related to cell proliferation, stress response and cell adhesion, as these pathways were notably enriched in NAC-responsive tumors. The identification of key predictors, such as *KEAP1*, *PCDHB9* and *POU2F2*, underscores the role of cellular stress and adhesion processes in chemotherapy resistance. This is consistent with previous reports suggesting that tumors that are resistant to chemotherapy often activate stress response pathways to evade cell death^14,28,35,36^. The link between cell proliferation and NAC response in MIBC has garnered increasing attention. Tumors with higher proliferative activity, as indicated by elevated levels of markers such as Ki-67 (ref. ^37^), tend to be more sensitive to cisplatin-based NAC^38^, probably due to the greater susceptibility of rapidly dividing cells to DNA-damaging agents. Conversely, lower proliferation rates are associated with poorer responses, potentially contributing to chemotherapy resistance and disease progression. The MYC protein, which regulates both cell proliferation and chemoresistance in various cancers^39,40^, was expressed at high levels in the tumor and stromal compartments of the chemo-sensitive patients in the AMC NAC cohort. Thus, further investigation into the precise role of MYC in modulating chemotherapy responses could provide critical insight for patient stratification and the design of personalized treatment strategies.

The machine learning approach developed in this study enabled the generation of data-driven classifiers from expression-based datasets, such as transcriptome or proteome profiles. It also facilitated the identification of the optimal number of biomarkers for stratifying the response to NAC through comparative evaluation with existing classifiers and a classifier reduction process. Validation of the biological and clinical relevance of the identified biomarkers led to the selection of candidate proteins for IHC analysis for computational pathology, which offers a powerful tool for quantifying protein expression and spatial heterogeneity within tumor samples, thereby addressing some of the limitations associated with traditional IHC analysis^41^.

In this study, we used computational pathology methods to evaluate the expression of proteins in tumor and stromal compartments. We identified biomarkers correlated with the response to NAC, such as KEAP1, PTPN12, RFX7, SIRT6 and USP2. In addition, the deep learning models developed, particularly those involving decision tree classifiers, identified optimal protein combinations for predicting NAC response, such as GLS, IL15RA, AFAP1 and FOXA1. These models provide a foundation for developing targeted IHC panels that could be implemented in the clinical setting. The robustness of these models was validated in advanced MIBC cases treated with preoperative chemotherapy followed by consolidative surgery, suggesting that they are generalizable and scalable to larger, more diverse patient populations.

Cisplatin-based NAC is usually administered over a period of 2–3 months, during which some patients experience disease progression due to a poor response, which decreases the likelihood of successful surgical intervention^42^. Consequently, the ability to predict the response to chemotherapy is paramount for identifying patients who are most likely to benefit from NAC versus those who may require immediate surgical management^43^. This predictive capacity would enable the implementation of precision medicine, allowing treatment strategies to be tailored in advance based on anticipated response, with treatment plans developed collaboratively with the patient and their caregiver. Furthermore, predictive biomarkers hold substantial potential as stratification factors in clinical trial design, guiding patient selection for novel investigations of both preoperative and postoperative systemic therapies, including intensification of neoadjuvant therapy or additional postoperative adjuvant therapy.

In addition to identifying predictive biomarkers, this study identified potential therapeutic targets. KEAP1, which promotes the ubiquitination and subsequent degradation of NRF2, a key regulator of the antioxidant response, was upregulated in chemotherapy-sensitive tumors, suggesting that targeting the NRF2–KEAP1 pathway could enhance the sensitivity of MIBC cells to cisplatin. Our mechanistic validation experiments provided functional evidence that targeting the KEAP1–NRF2 axis can overcome chemoresistance by impairing tumor GSH dynamics and cellular plasticity. Enforcing *KEAP1* expression in cisplatin-resistant MIBC cells (or pharmacologically inhibiting NRF2) led to a marked suppression of the NRF2 pathway, which in turn dampened GSH metabolic activity and the associated antioxidant capacity of these cells. Specifically, KEAP1 restoration reduced NRF2 occupancy at the promoters of GSH biosynthetic genes and lowered the expression of key GSH-related enzymes, effectively collapsing the heightened GSH dynamics in chemoresistant MIBC cells. This disruption of the NRF2-driven GSH program is crucial as high intracellular GSH dynamics are known to be required for maintaining cancer stem cell functions^14^. Consistent with this, we observed that KEAP1–NRF2 pathway inhibition curtailed the stemness features of chemoresistant MIBC cells, based on the findings that *KEAP1* upregulation or NRF2 blockade significantly reduced tumor sphere formation, clonogenic growth and invasiveness, hallmarks of the stem cell-like, therapy-tolerant state.

Notably, the loss of these survival advantages translated into enhanced therapeutic response. Suppression of NRF2-mediated cytoprotective signaling enhanced cisplatin-induced DNA damage and apoptosis, as demonstrated by increased levels of cleaved caspase-3 and PARP in vitro, along with a pronounced restoration of drug sensitivity in vivo. In an orthotopic xenograft model of chemoresistant MIBC, the combination of cisplatin with KEAP1–NRF2 inhibitors yielded dramatic tumor regression and extinguished expression of GSH-related enzymes and cancer stem cell markers in the residual tumors, which were not achieved by cisplatin or NRF2 inhibition alone. These findings strongly support that KEAP1–NRF2 signaling sustains a redox-rich, stem cell-like tumor state that underlies cisplatin resistance. Taken together, our results provide a mechanistic rationale for KEAP1–NRF2-targeted interventions in MIBC and highlight how abrogating tumor antioxidant defenses and cell plasticity can synergize with conventional chemotherapy to yield superior anticancer effects. These findings provide a rationale for further exploration of NRF2–KEAP1 inhibitors as a novel adjunct therapy in cisplatin-resistant MIBC, potentially improving the outcomes of patients who do not respond to standard NAC regimens.

Although this study provides valuable information for NAC response prediction and biomarker discovery, several limitations warrant consideration. First, despite the successful cross-validation of the machine learning models in multiple cohorts, the small sample size of certain datasets, such as the AMC discovery cohort, may limit the generalizability of some findings. Larger, prospective clinical trials are needed to validate the efficacy of the models. Second, the integration of multimodal omics data requires further refinement to ensure that these predictive models can be seamlessly implemented in clinical practice. The development of user-friendly computational tools for clinicians will be critical for translating these findings into personalized treatment strategies. Despite the identification of several promising therapeutic targets, the efficacy and safety of NRF2–KEAP1 inhibitors and other targeted therapies should be further explored in preclinical and clinical settings to determine their potential to enhance NAC efficacy in patients with MIBC.

In summary, this study provides a comprehensive framework for predicting the response to NAC in MIBC by integrating machine learning, digital pathology and multimodal omics analysis. The identification of key biomarkers and refining of molecular classifiers could lead to the development of personalized and effective treatment strategies for patients with MIBC. Further research is needed to validate these findings in larger cohorts and explore the therapeutic potential of targeting the identified pathways, particularly in chemotherapy-resistant tumors. In addition, the treatment landscape for MIBC is rapidly evolving, and promising new approaches using immunotherapy and antibody–drug conjugates alone or in combination with NAC are emerging^44–47^. These advances offer the potential to substantially improve patient outcomes. Therefore, it will be interesting to see how the workflow of this study will be applied in this dynamic therapeutic landscape to advance treatment.

## Supplementary information

Supplementary Information

Supplementary Tables

## Source data

Source data

## Data Availability

All raw and processed transcriptome data from the AMC cohort, along with clinical annotations, are available in the NCBI Gene Expression Omnibus (GSE212810). Transcriptome datasets and pathological responses for three external cohorts were retrieved from GEO under accession numbers GSE48277 (MDA MVAC), GSE69795 (MDA DDMVAC) and GSE87304 (NAC metadata). The records of the pathological responses of the ‘NAC metadata’ cohort were kindly provided by Dr. Peter C. Black (University of British Columbia, Canada). Details of the GO categories and genes used in functional analyses are listed in Source data provided as a separate Excel file. For computational pathology analysis of the AMC cohorts, the expression data for each protein in the tumor and stromal compartments applied with the tumor/stroma classifier^14^, together with relevant clinical annotations, are found in Supplementary Tables 2 and 3. Source codes used for machine learning modeling of transcriptome and computational pathology datasets are available via Zenodo at https://zenodo.org/records/14603074 (ref. ^48^) and https://zenodo.org/records/14603169 (ref. ^49^), respectively. Any additional information required to reanalyze the data reported in this paper is available from the lead contact upon request.
