# Supplementary material for: Machine learning-based integration of transcriptome and digital pathology for predicting chemoresistance in muscle-invasive bladder cancer
Source: Exp Mol Med. 2026 May 8;58(5):1589–607. doi: 10.1038/s12276-026-01718-y (PMC13234436; doi:10.1038/s12276-026-01718-y)
Supplement: Supplementary file 1 — Supplementary Information [file 12276_2026_1718_MOESM1_ESM.pdf]

Supplementary Information

Machine learning-based integration of transcriptome and digital pathology for predicting chemoresistance in muscle-invasive bladder cancer

Corresponding authors. d0shin03@amc.seoul.kr, yongcho@amc.seoul.kr, and bshong@amc.seoul.kr

This PDF file includes:

Supplementary Fig. 1 to 16 and figure legends

Supplementary Table 1 to 10 and table legends

Supplementary Materials and Methods

Supplementary References

Key resources table

Uncropped WB

Other Supplementary Information includes a separate file including

Supplementary Table 1–10

Source dataset. Source data for quantification analyses

SUPPLEMENTARY FIGURE LEGENDS

a

AMC discovery	Prediction score
NAC_R #1	0.926
NAC_R #2	0.992
NAC_R #3	0.940
NAC_R #4	0.972
NAC_R #5	0.962
NAC_R #6	0.965
NAC_R #7	0.942
NAC_R #8	0.971
NAC_NR #1	0.063
NAC_NR #2	0.005
NAC_NR #3	0.049
NAC_NR #4	0.036
NAC_NR #5	0.026
NAC_NR #6	0.108
NAC_NR #7	0.026
NAC_NR #8	0.019

b

Feature	Importance value
LOC440311	0.014
MAD2L1	0.012
ZNF793	0.008
GZMH	0.008
UGCG	0.008
PCBD1	0.008
MRM1	0.008
ZNF764	0.008
DLG1	0.007
QARS	0.007
TMEM199	0.006
LINC00504	0.006
HIPK2	0.006
DHRS4-AS1	0.006
UCKL1	0.006

c

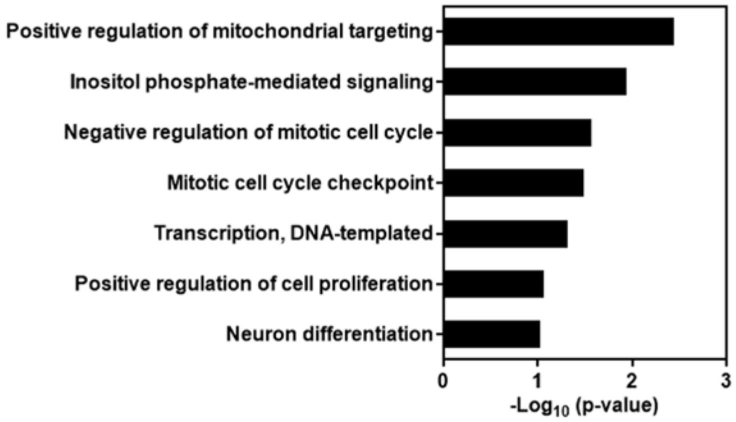

d

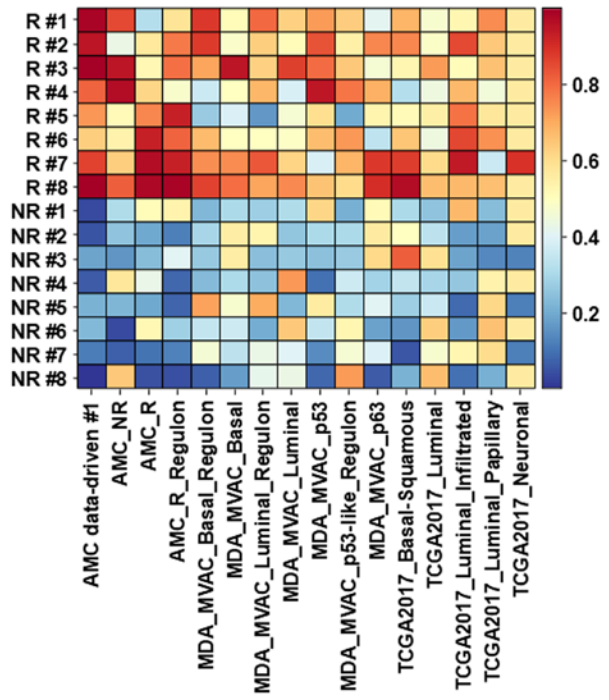

e

Classifier	AUC		Performance difference (Δ AUC)
	Classifier (5 < n < 30)	Reduction (n < 10)	
AMC data-driven #1	1.000	1.000	0.00
AMC data-driven #2	1.000	-	-
AMC_NR	1.000	0.891	0.109
AMC_R	1.000	0.953	0.047
AMC_R_Regulon	0.969	0.984	-0.015
MDA_MVAC_Basal_Regulon	0.938	0.875	0.063
MDA_MVAC_Basal	0.734	0.828	-0.094
MDA_MVAC_Luminal_Regulon	0.906	0.797	0.109
MDA_MVAC_Luminal	0.969	0.781	0.188
MDA_MVAC_p53	1.000	0.953	0.047
MDA_MVAC_p53-like_Regulon	0.953	0.797	0.156
MDA_MVAC_p63	0.969	0.797	0.172
TCGA2017_Basal_Squamous	1.000	0.875	0.125
TCGA2017_Luminal	1.000	0.688	0.312
TCGA2017_Luminal_Infiltrated	1.000	0.930	0.070
TCGA2017_Luminal_Papillary	0.906	0.805	0.101
TCGA2017_Neuronal	1.000	0.727	0.273

Supplementary Figure 1. Developing and optimizing gene classifiers for the AMC discovery cohort

(a–c) Random forest modeling of transcriptome datasets from the AMC discovery cohort (*n* = 8/group; No-response: NR, and Response: R). **(a)** Average prediction score (NR = 0 and R = 1) for the 10 random forest models generated. **(b and c)** Importance values **(b)** and gene ontology (GO) analysis **(c)** of 25 genes extracted as AMC data-driven classifier_#1. **(d)** Heatmap of the

prediction scores in the logistic regression model of the AMC discovery cohort with the indicated gene classifiers after reduction to fewer than 10 genes. **(e)** Comparison of the AUC (area under the ROC curve) for the performance of each classifier before and after the reduction process. The heatmap analysis results before classifier reduction are presented in **Fig. 1b**. Detailed information on the gene classifiers used in each modeling algorithm is presented in **Supplementary Tables 4 and 5**. *Related to Fig. 1.*

a

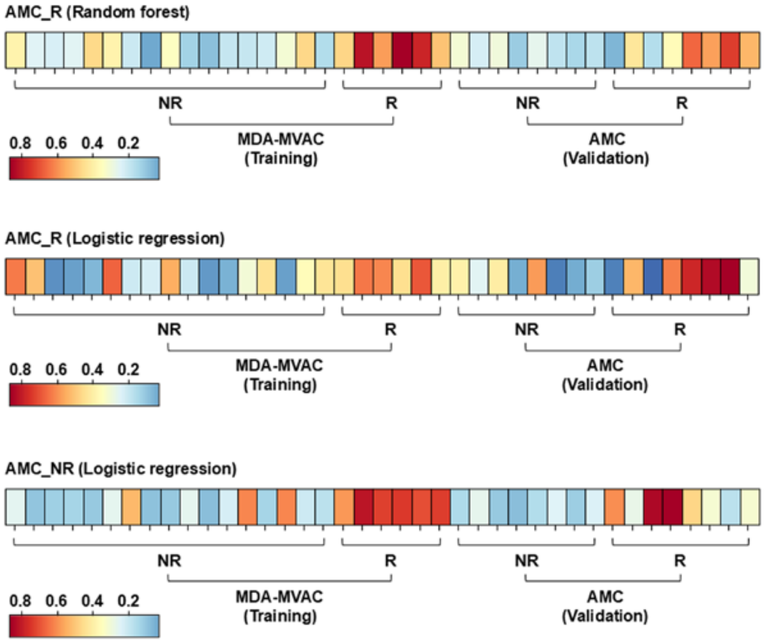

b

Classifier	Algorithm	Data (Training, Validation)	AUC	
			Training	Validation
AMC_R	Random forest	MDA MVAC, AMC	1.000	0.781
AMC_R	Logistic regression	MDA MVAC, AMC	0.833	0.703
AMC_NR	Logistic regression	MDA MVAC, AMC	0.980	0.953

c

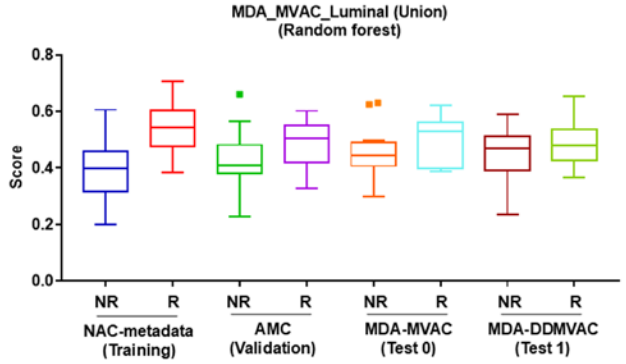

d

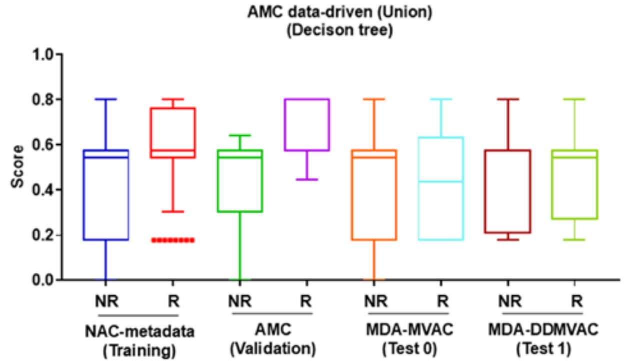

e

Algorithm	Decision tree	Logistic regression	Decision tree
Classifier	AMC data-driven (Union)		
Variables #	Reduction to 28	Reduction to 15	Reduction to 6
AUC (Training)	0.881	0.624	0.801
AUC (Validation)	0.814	0.647	0.741

f

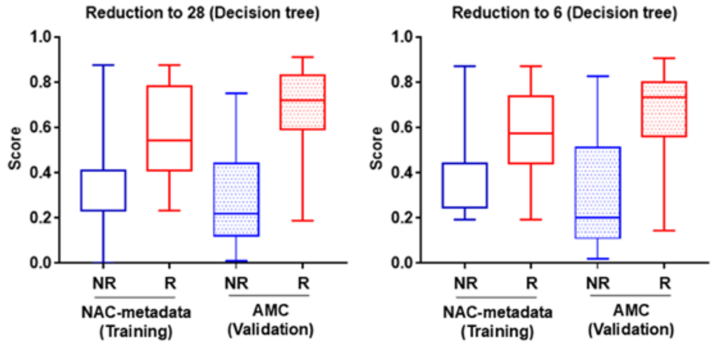

Supplementary Figure 2. Multi-cohort cross-validation of the transcriptome machine learning models

(a and b) Heatmap of the prediction scores **(a)** and summary of the AUC values **(b)** for the indicated modeling algorithms, which showed the significant performance in the cross-validation between the MDA-MVAC (training) and AMC (validation) cohorts. **(c and d)** Box plots of the prediction scores for the random forest model with the union MDA_MVAC_Luminal gene classifier using the AMC cohort as a test set **(c)** or for decision

tree modeling with the union AMC data-driven classifier using the AMC cohort as a validation set **(d)** in the multi-cohort cross-validation analysis including 17 gene classifiers and the transcriptome datasets of AMC and three independent external cohorts. **(e and f)** Summary of AUC values **(e)** and box plots of the prediction scores **(f)** for the indicated modeling algorithms for the classifier reduction process identified by cross-validation between NAC metadata (training) and AMC (validation) cohorts. Detailed information on the gene classifiers for each modeling algorithm and the key features of each classifier reduction model are presented in **Supplementary Tables 6 and 7**, respectively. *Related to Fig. 1.*

a

Related to stress response

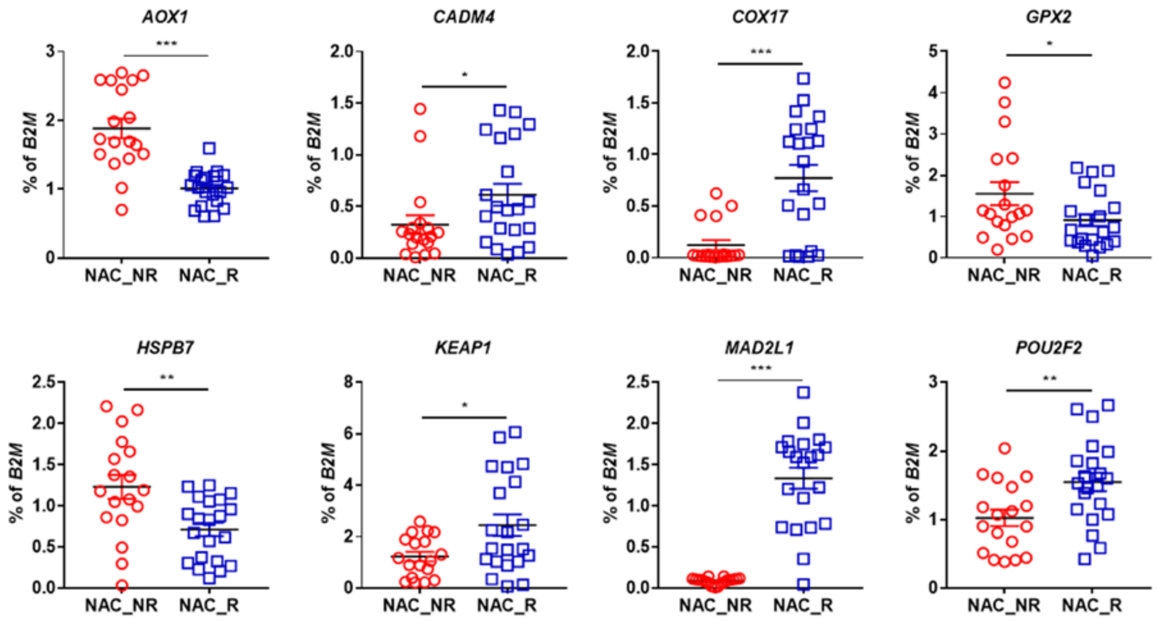

b

Related to cell adhesion and motility

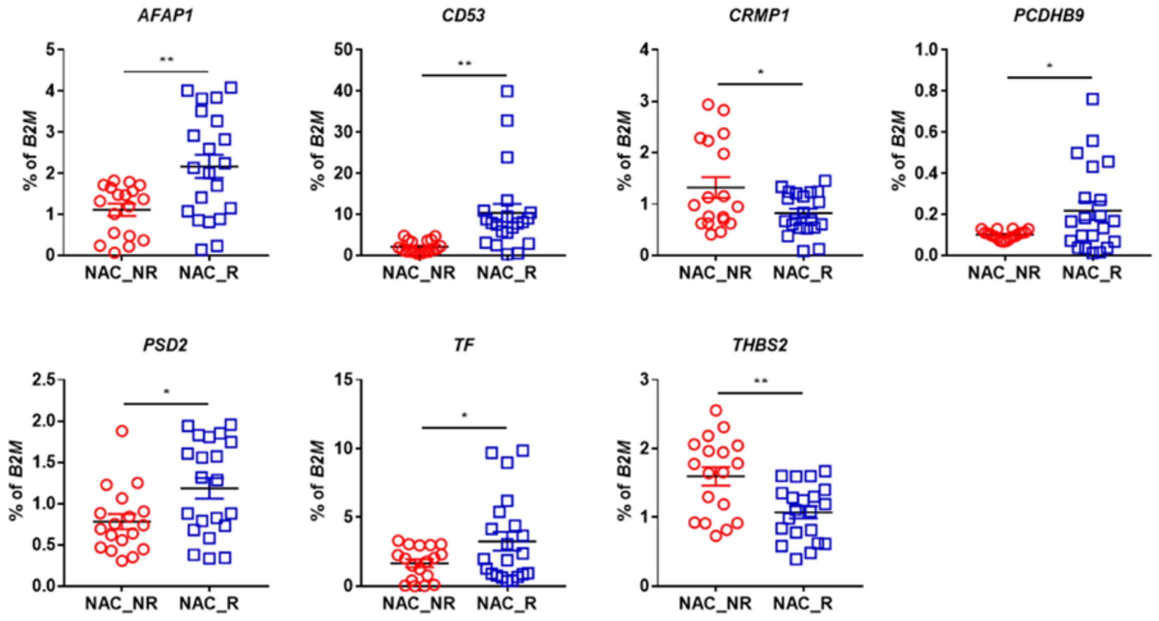

c

Related to transcription

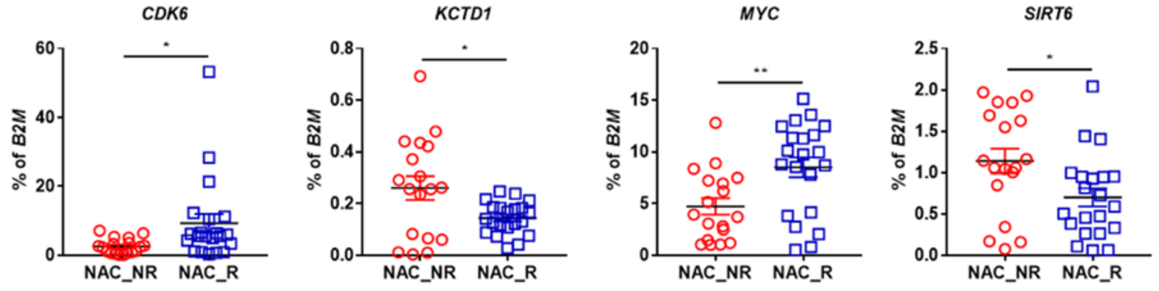

Supplementary Figure 3. Validation of the expression of the biomarkers characterizing the NAC response

(a–c) qPCR analysis was performed to validate the expression of a subset of biomarker genes derived from the multi-cohort transcriptome machine learning analysis for stratifying the NAC response in AMC cohort patients (NR $n = 18$, R $n = 21$), with the selected biomarkers associated with stress response **(a)**, cell adhesion and motility **(b)**, and transcription **(c)**. The expression of genes is presented as percent relative to human β2-microglobulin (*B2M*) expression. Quantitative results are shown as dot plots of the mean ± SEM; $*p < 0.05$, $**p < 0.01$, $***p < 0.001$, unpaired Student's t test. The exact p-values and number of replicates are specified in the **Source datasets**. *Related to Fig. 2.*

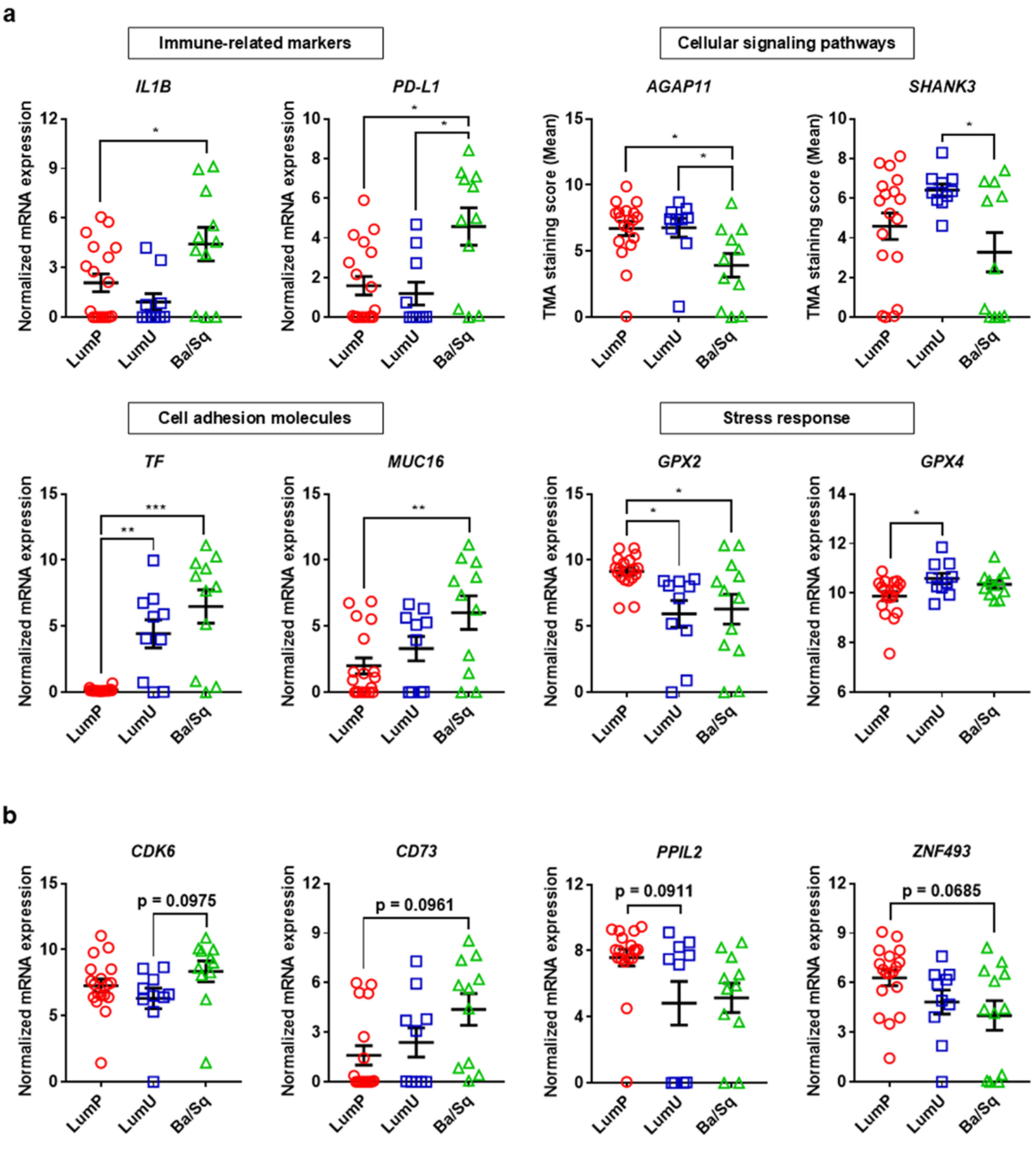

Supplementary Figure 4. Expression of the validated biomarkers for consensus subtype classification

(a and b) Expression levels of the indicated biomarker genes in the AMC validation cohort following stratifying three consensus gene expression subtypes (LumP *n* = 18, LumU *n* = 10, and Ba/Sq *n* = 11). Transcript levels were extracted from datasets of QuantSeq 3' mRNA-Seq dataset. Quantitative results are presented as dot plots of mean ± SEM. *p < 0.05, **p < 0.01, ***p < 0.001, one-way ANOVA with Bonferroni post-hoc test. The exact experimental values, fold change, p-values, and numbers of replicates are presented in the **Source datasets**. *Related to Fig. 2.*

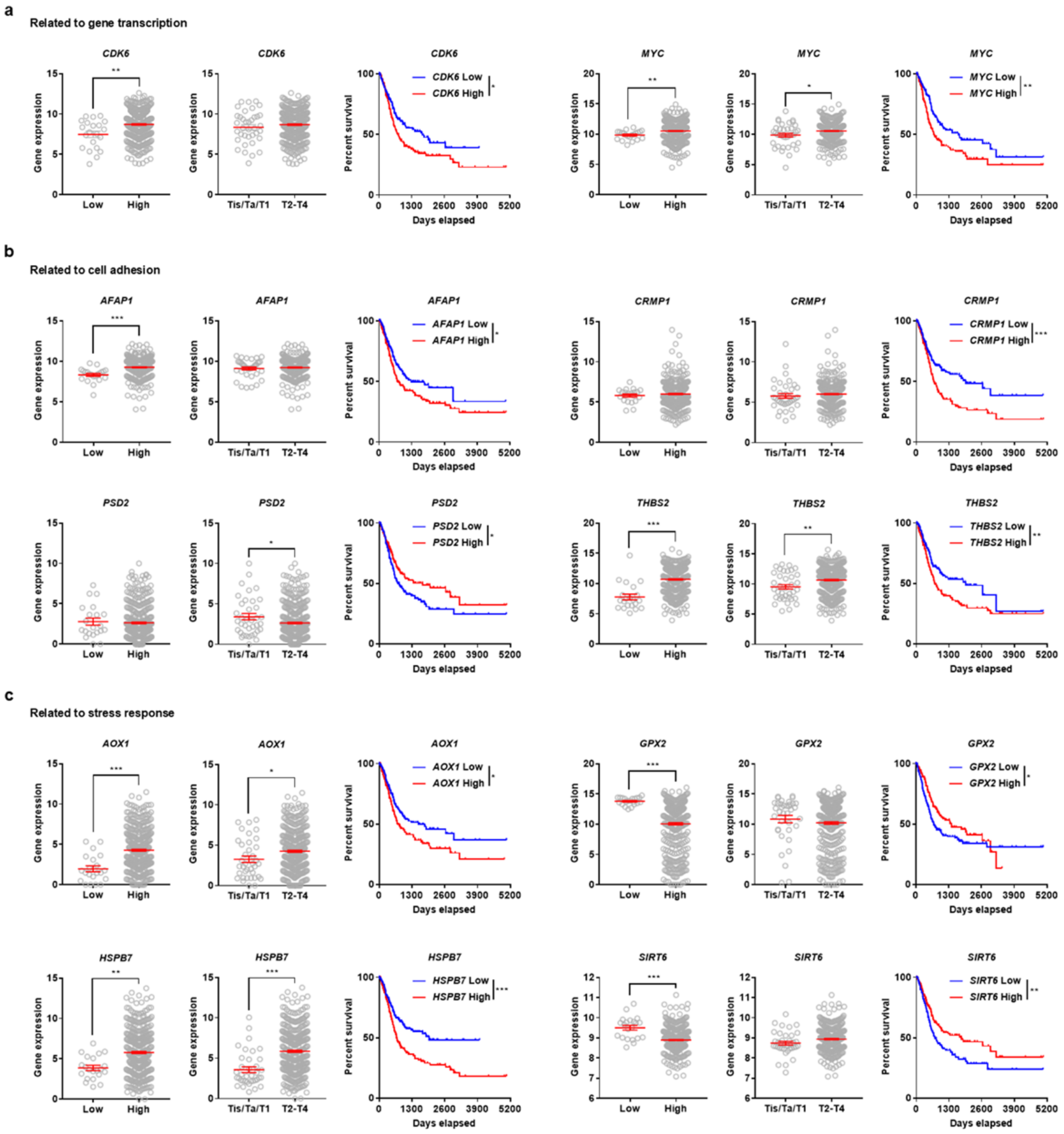

Supplementary Figure 5. Clinical significance of the validated biomarkers

(a–c) Analysis of validated biomarkers related to gene transcription **(a)**, cell adhesion **(b)**, and

stress response **(c)** in subgroups of BC patients according to expression levels and tumor grade (left panel), pT category (middle panel), and overall survival (right panel). Gene expression raw data were obtained from UCSC's Xena project (http://xena.ucsc.edu/). Quantitative results are shown as the mean ± SEM; $*p < 0.05$, $**p < 0.01$, $***p < 0.001$. *Related to Fig. 2.*

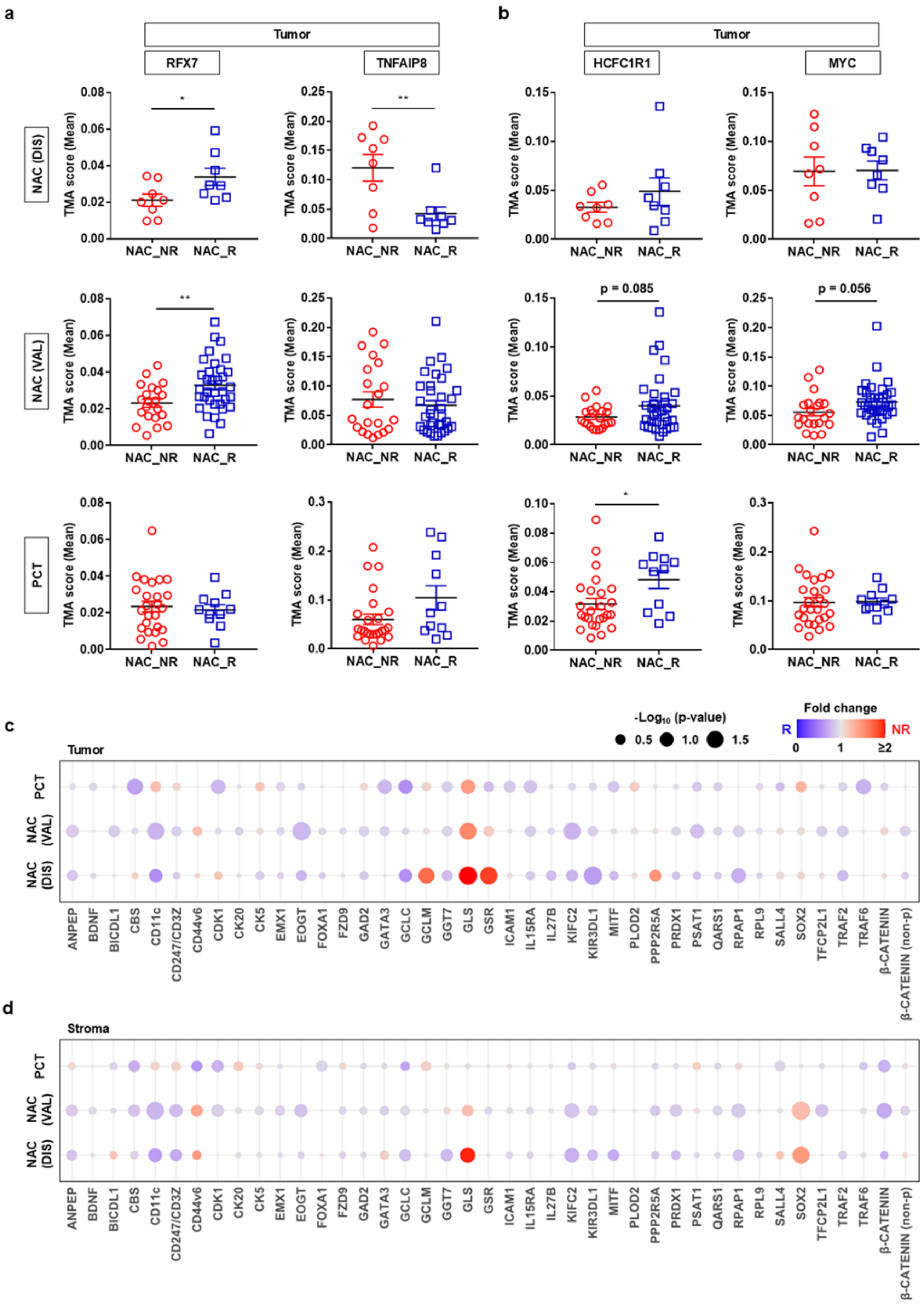
a
Tumor
RFX7
TNFAIP8
b
Tumor
HCFC1R1
MYC
NAC (DIS)
NAC (VAL)
PCT
TMA score (Mean)
NAC_NR
NAC_R
p = 0.085
p = 0.056
c
Tumor
-Log10 (p-value)
0.5 1.0 1.5
Fold change
R
NR
0 1 ≥2
ANPEP BDNF BICDL1 CBS CD11c CD247/CD3Z CD44v6 CDK1 CK20 CK5 EMX1 EOGT FOXA1 FZD9 GAD2 GATA3 GCLC GCLM GGT7 GLS GSR ICAM1 IL15RA IL27B KIFC2 KIR3DL1 MITF PLOD2 PPP2R5A PRDX1 PSAT1 QARS1 RPAP1 RPL9 SALL4 SOX2 TFCP2L1 TRAF2 TRAF6 β-CATENIN β-CATENIN (non-p)
d
Stroma

Supplementary Figure 6. Digital pathology analysis of biomarkers for predicting NAC response

(a and b) Expression levels of RFX7, TNFAIP8, HCFC1R1, and MYC proteins at the tumorous epithelial cells in the indicated AMC cohorts. **(a)** RFX7 and TNFAIP8 proteins were differentially expressed according to NAC response in the AMC discovery cohort (*n* = 8/group, NR and R). **(b)** The expression of HCFC1R1 and MYC was higher in R group tumors, although the statistical significance was marginal. Quantitative results are presented as dot plots of the mean ± SEM (∗$p < 0.05$, ∗∗$p < 0.01$, unpaired Student's t test). **(c and d)** Digital pathology for the analysis of spatial protein expression in the tumorous epithelial cells **(c)** and the stroma compartment **(d)** in the AMC NAC discovery (DIS) and validation (VAL) cohorts (NR *n* = 21, R *n* = 34) or PCT cohort (NR *n* = 25, R *n* = 11). Bubble plot of the digital pathology analyses of 41 proteins from our previous study (Kim et al., 2023) at the tumorous epithelial cells **(c)** and the stromal compartment **(d)** in the indicated AMC cohorts. The expression levels of 74 proteins in AMC NAC (*n* = 55) and PCT (*n* = 36) cohort patients at the tumor and stromal compartments and clinical annotations are presented in **Supplementary Tables 2 and 3**, respectively. *Related to Fig. 3.*

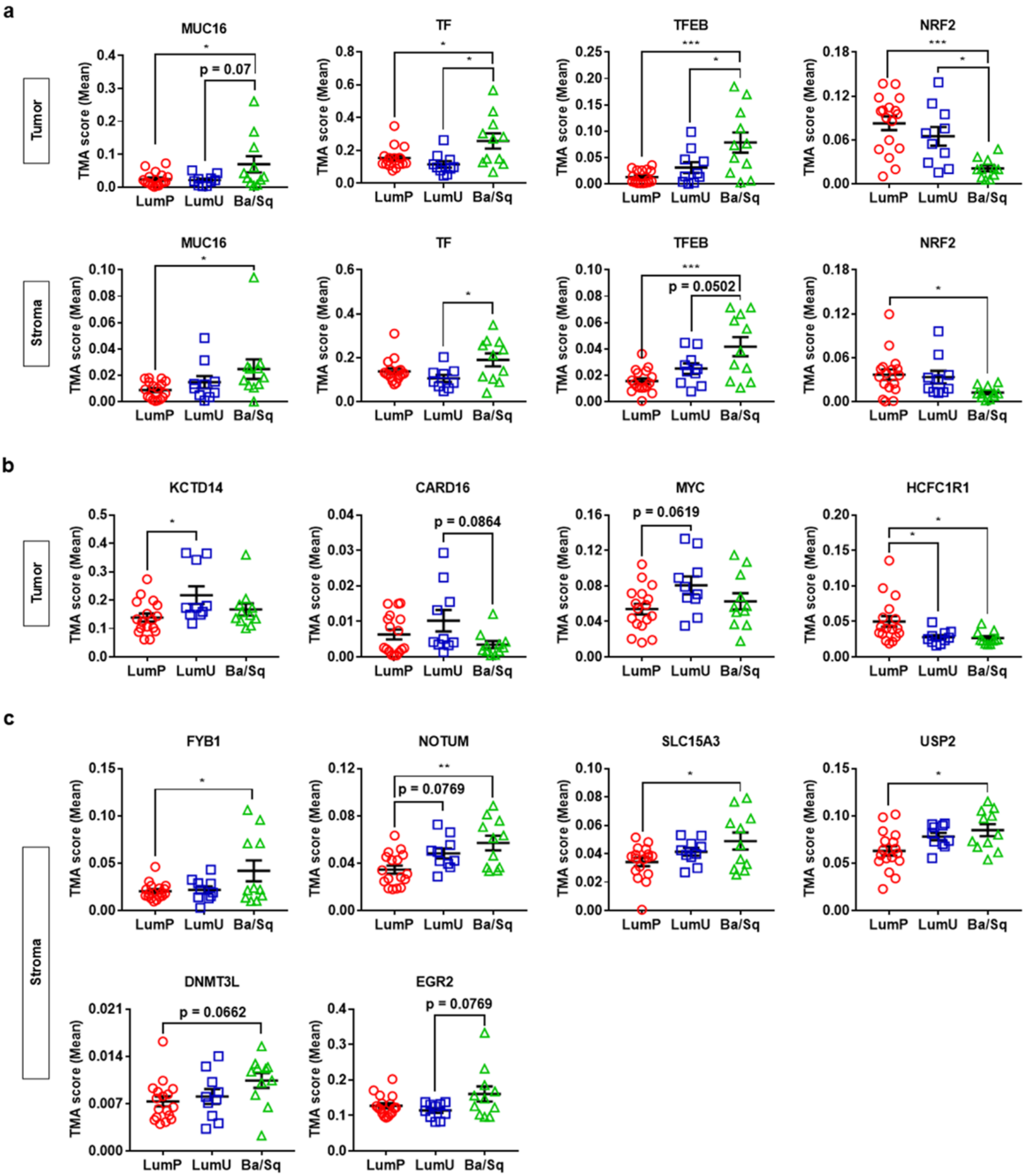
a
Tumor
Stroma
MUC16
TF
TFEB
NRF2
p = 0.07
p = 0.0502
TMA score (Mean)
LumP
LumU
Ba/Sq
b
Tumor
KCTD14
CARD16
MYC
HCFC1R1
p = 0.0864
p = 0.0619
c
Stroma
FYB1
NOTUM
SLC15A3
USP2
DNMT3L
EGR2
p = 0.0769
p = 0.0662
p = 0.0769

Supplementary Figure 7. Association with the MIBC consensus subtypes and spatial expression of the biomarkers for predicting NAC response

(a) Expression levels of the indicated biomarker proteins, which are significantly different in the Ba/Sq subtype, in both the tumor (upper panel) and stromal (lower panel) compartments. **(b and c)** Expression levels of the indicated biomarker proteins in the tumor **(b)** and stroma **(c)** compartments in the three identified consensus gene expression subtypes of the AMC validation cohort. Quantitative results are presented as dot plots of mean ± SEM. *p < 0.05, **p < 0.01, one-way ANOVA with Bonferroni post hoc test. *Related to Fig. 3.*

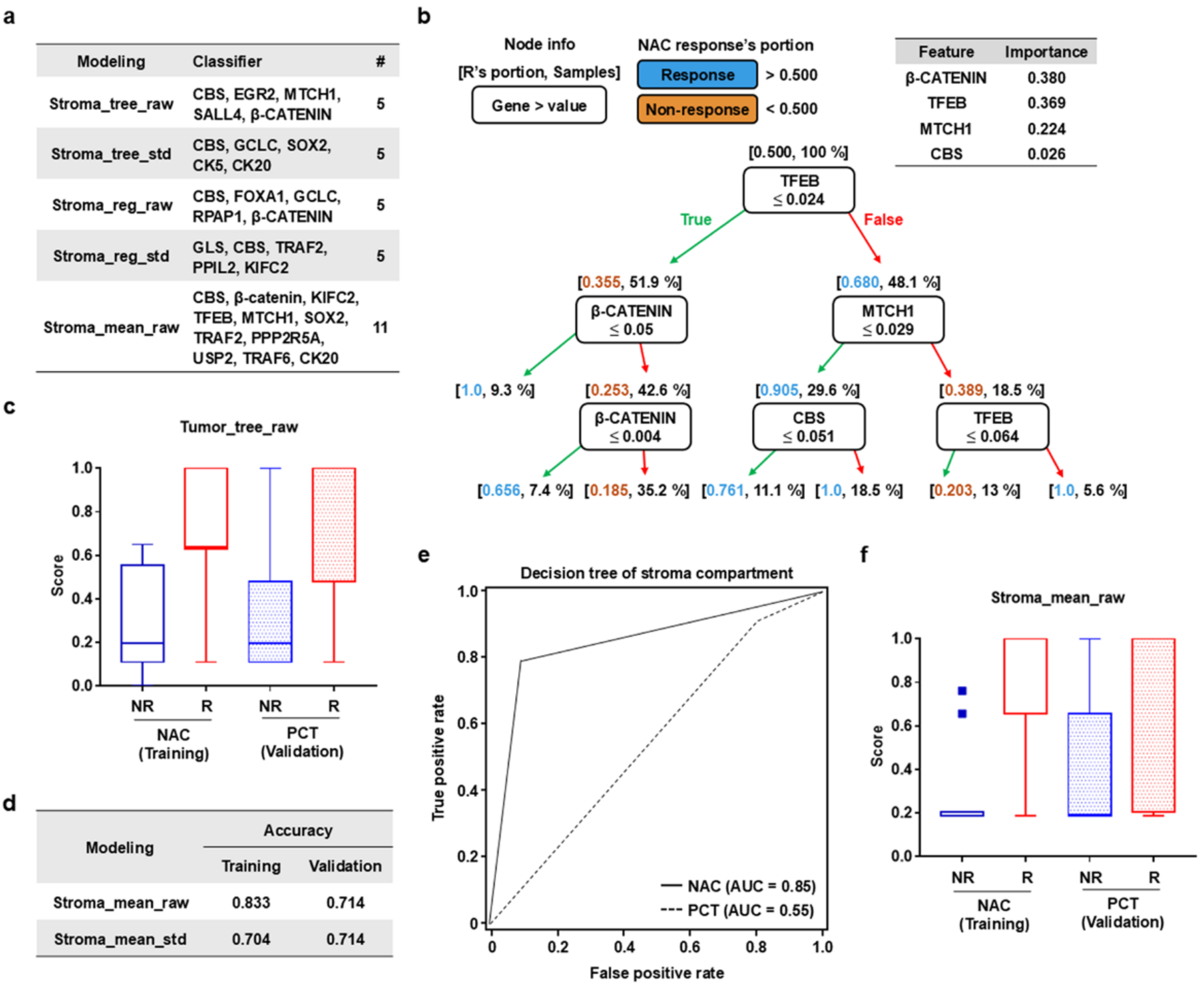

Supplementary Figure 8. Prediction modeling for digital pathology of the stromal compartment

(a) Summary of the modeling methods, data type, and list or number of proteins corresponding to the biomarkers assigned the highest weight in each modeling algorithm using the digital pathology datasets in the stromal compartment. **(b)** Decision tree model of the stromal compartment with the key proteins for a decision rule; the corresponding outcome branches are indicated in different colors (true = green, false = red), and terminal nodes show the

predicted NAC response (Response = blue, Non-response = orange) and the frequency of samples. The importance of key features is presented with the decision tree. **(c–f)** Validation of prediction models using computational pathology of tumor and stromal compartments. **(c)** Boxplots of the prediction scores for the decision tree modeling of raw data from the tumor compartment (Tumor_tree_raw model) in the cross-validation between AMC NAC (training) and PCT (validation) cohorts. **(d–f)** Accuracy **(d)**, ROC curve **(e)**, and boxplots of the prediction scores **(f)** demonstrating the performance of the model according to protein expression in the stromal compartment for NAC response prediction in the cross-validation between AMC NAC and PCT cohorts. *Related to Fig. 4.*

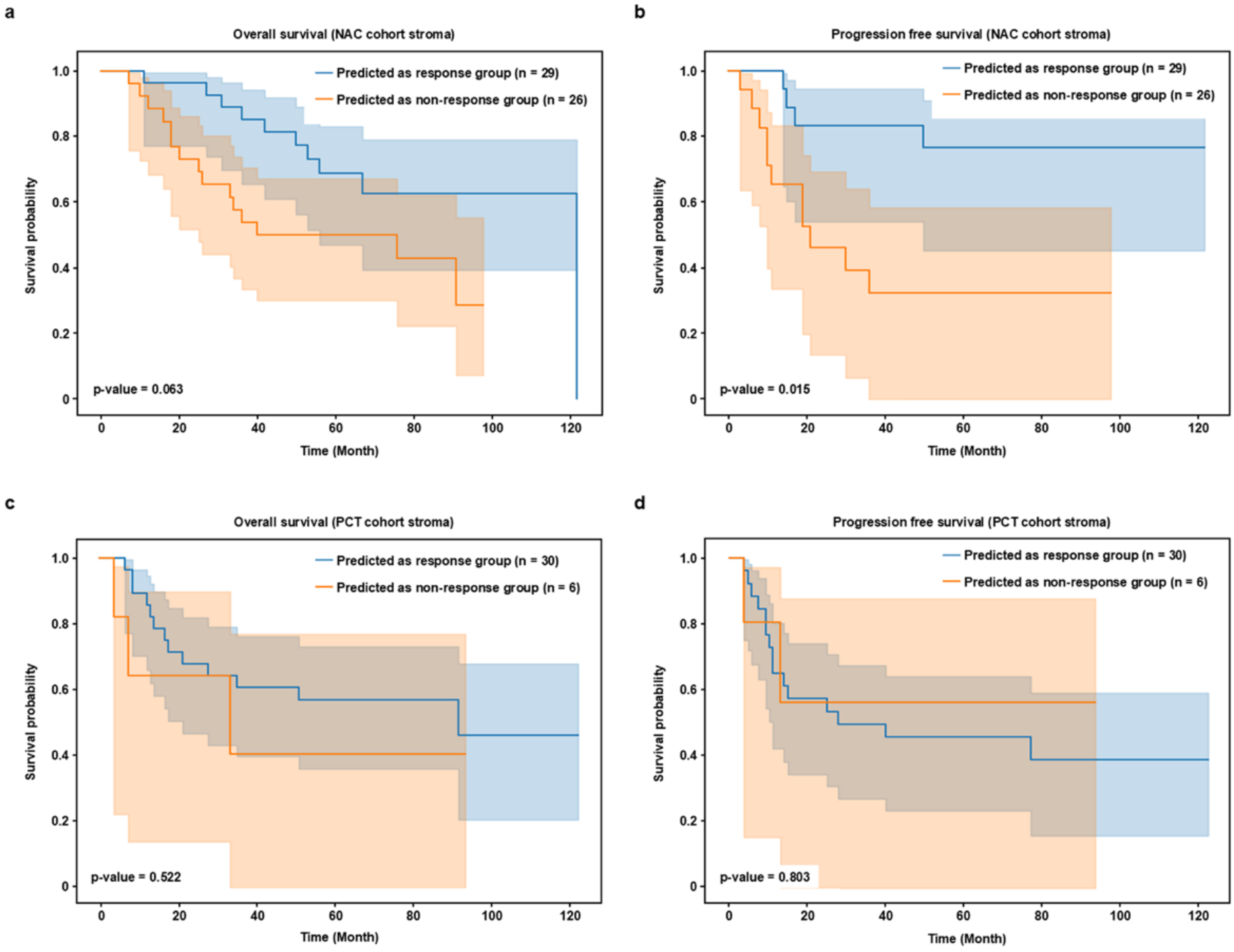

Supplementary Figure 9. Survival outcomes based on computational pathology prediction model

Kaplan–Meier survival curves depicting overall survival **(a and c)** and progression-free survival **(b and d)** stratified by predicted NAC response status in the AMC NAC **(a and b)** and PCT **(c and d)** cohorts. Predictions were derived from decision tree models applied to computational pathology datasets focusing on stromal compartment biomarkers. *Related to Fig. 4.*

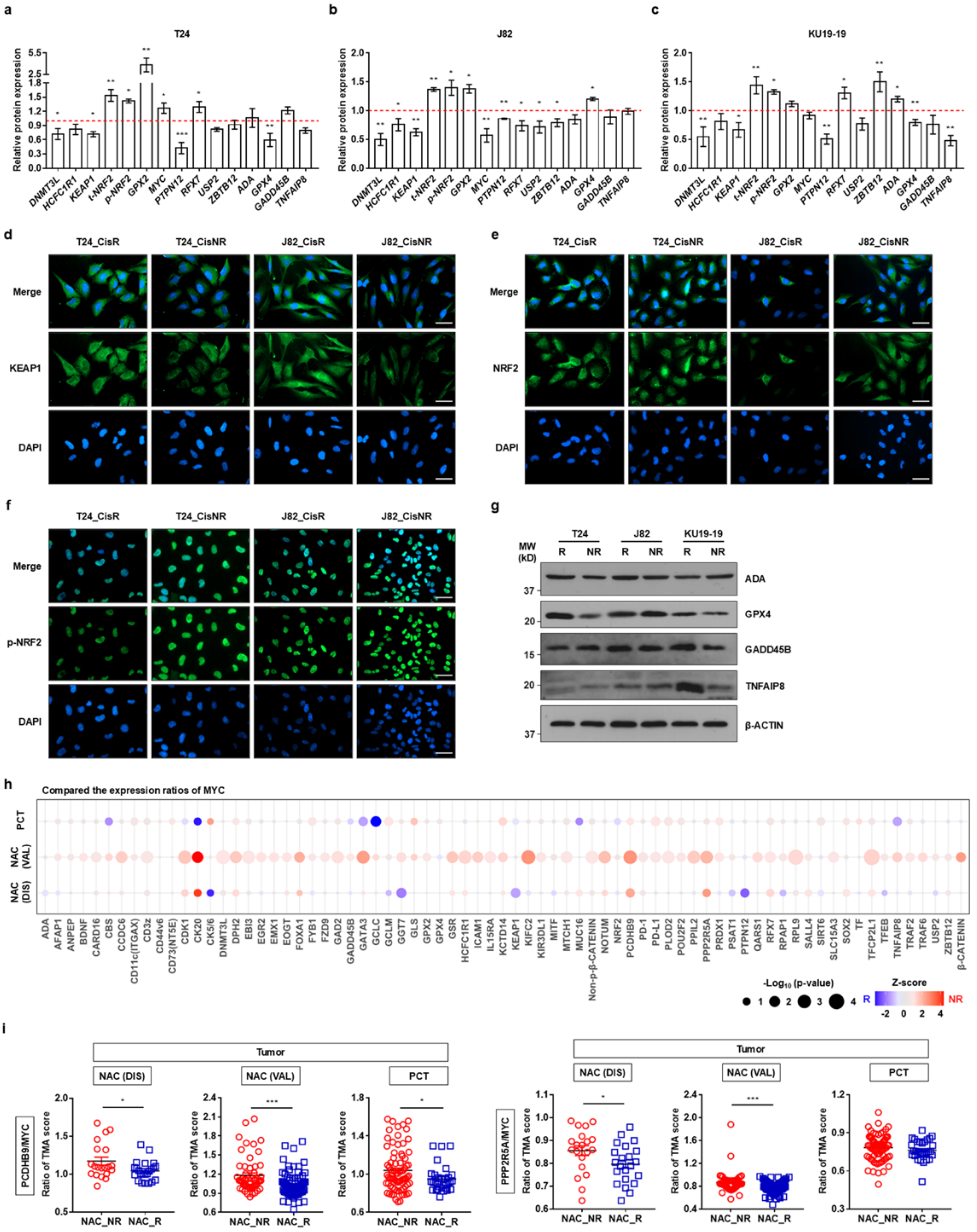
a
T24
Relative protein expression
b
J82
c
KU19-19
d
T24_CisR
T24_CisNR
J82_CisR
J82_CisNR
Merge
KEAP1
DAPI
e
NRF2
f
p-NRF2
g
MW (kD)
T24
J82
KU19-19
R
NR
ADA
GPX4
GADD45B
TNFAIP8
β-ACTIN
h
Compared the expression ratios of MYC
PCT
NAC (VAL)
NAC (DIS)
-Log10 (p-value)
Z-score
i
Tumor
NAC (DIS)
NAC (VAL)
PCT
PCDHB9/MYC
PPP2R5A/MYC
Ratio of TMA score
NAC_NR
NAC_R

Supplementary Figure 10. Activation of the KEAP1–NRF2 pathway in cisplatin-resistant MIBC cells.

(a–c) Densitometric quantification of the Western blot bands presented in **Figure 5a** for T24 **(a)**, J82 **(b)**, and KU19-19 **(c)** cell lines. Protein expression levels were normalized to the respective loading controls (β-ACTIN) and are expressed as fold changes relative to the cisplatin-responsive (CisR) cells (set to 1; see the red dotted line) (n = 3). **(d–f)** Representative immunofluorescence images showing KEAP1 **(d)** and total or phosphorylated NRF2 **(e and f)** protein expression (green) in cisplatin-sensitive (Cis_R) and cisplatin-resistant (Cis_NR) T24 and J82 MIBC cells. Nuclei were counterstained with DAPI (blue). Images were acquired at ×400 magnification; scale bars = 200 μm. **(e and f)** Notably, the nuclear translocation of total **(e)** or phosphorylated **(f)** NRF2 proteins (green) was markedly increased in Cis_NR cells relative to Cis_R counterparts. **(g)** Western blot analysis of clinically relevant biomarker proteins in cisplatin responsive (Cis_R) and non-responsive (Cis_NR) MIBC cell lines. β-ACTIN was used as a loading control. Molecular weight (MW) marker sizes (kD) are shown on the left. **(h and i)** Protein expression ratios relative to MYC determined by computational pathology of the tumor compartment. **(h)** Bubble plot displaying Z-scores and the statistical significance of protein expression ratios relative to MYC in NR and R groups in the AMC NAC and PCT cohorts. **(i)** Dot plots showing protein expression ratios for PCDHB9/MYC (left) and PPP2R5A/MYC (right) in the AMC cohorts. Ratios were calculated using values from three independent TMA cores per patient. Data are presented as mean ± SEM. Statistical significance was determined by unpaired Student's t-test (I) and one-way ANOVA **(a–c)** with Bonferroni post hoc test. *$p < 0.05$, **$p < 0.01$, ***$p < 0.001$. *Related to Fig. 5.*

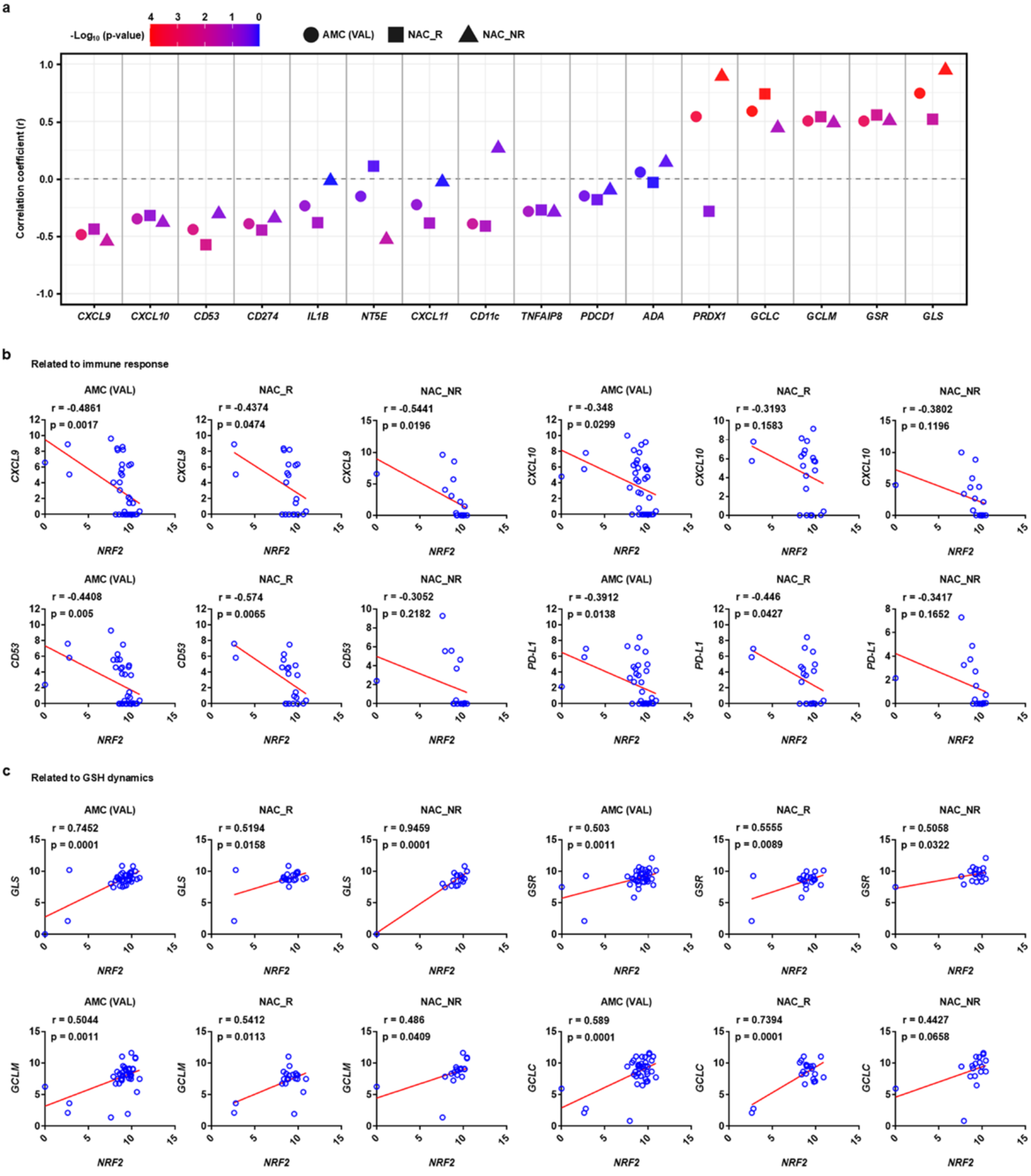
a
-Log10 (p-value)
AMC (VAL)
NAC_R
NAC_NR
Correlation coefficient (r)
CXCL9
CXCL10
CD53
CD274
IL1B
NT5E
CXCL11
CD11c
TNFAIP8
PDCD1
ADA
PRDX1
GCLC
GCLM
GSR
GLS
b
Related to immune response
AMC (VAL) r = -0.4861 p = 0.0017
NAC_R r = -0.4374 p = 0.0474
NAC_NR r = -0.5441 p = 0.0196
AMC (VAL) r = -0.348 p = 0.0299
NAC_R r = -0.3193 p = 0.1583
NAC_NR r = -0.3802 p = 0.1196
AMC (VAL) r = -0.4408 p = 0.005
NAC_R r = -0.574 p = 0.0065
NAC_NR r = -0.3052 p = 0.2182
AMC (VAL) r = -0.3912 p = 0.0138
NAC_R r = -0.446 p = 0.0427
NAC_NR r = -0.3417 p = 0.1652
PD-L1
NRF2
c
Related to GSH dynamics
AMC (VAL) r = 0.7452 p = 0.0001
NAC_R r = 0.5194 p = 0.0158
NAC_NR r = 0.9459 p = 0.0001
AMC (VAL) r = 0.503 p = 0.0011
NAC_R r = 0.5555 p = 0.0089
NAC_NR r = 0.5058 p = 0.0322
AMC (VAL) r = 0.5044 p = 0.0011
NAC_R r = 0.5412 p = 0.0113
NAC_NR r = 0.486 p = 0.0409
AMC (VAL) r = 0.589 p = 0.0001
NAC_R r = 0.7394 p = 0.0001
NAC_NR r = 0.4427 p = 0.0658

Supplementary Figure 11. Correlation between *NRF2* expression and immune-related markers.

(a) Integrated bar plot showing the correlation coefficients (r) and their corresponding significance ($-\log_{10}$ p-value) between *NRF2*/*NFE2L2* expression and various genes related to immune response or GSH dynamics in the LCMD-derived AMC validation cohort (AMC-VAL). **(b)** Scatter plots illustrating the inverse correlation between *NRF2* and key Th1-type chemokines (*CXCL9* and *CXCL10*) and immune markers (*CD53*, *CD11c*, and *CD274*/*PD-L1*) in responder (NAC_R) and non-responder (NAC_NR) groups. Pearson correlation coefficients (r) and p-values are indicated for each subgroup. **(c)** Scatter plots showing the positive correlation between *NRF2*/*NFE2L2* and genes involved in GSH dynamics (*GCLC*, *GCLM*, *GSR*, and *GLS*) in both responder and non-responder groups. Statistical significance was determined using Pearson's correlation analysis.

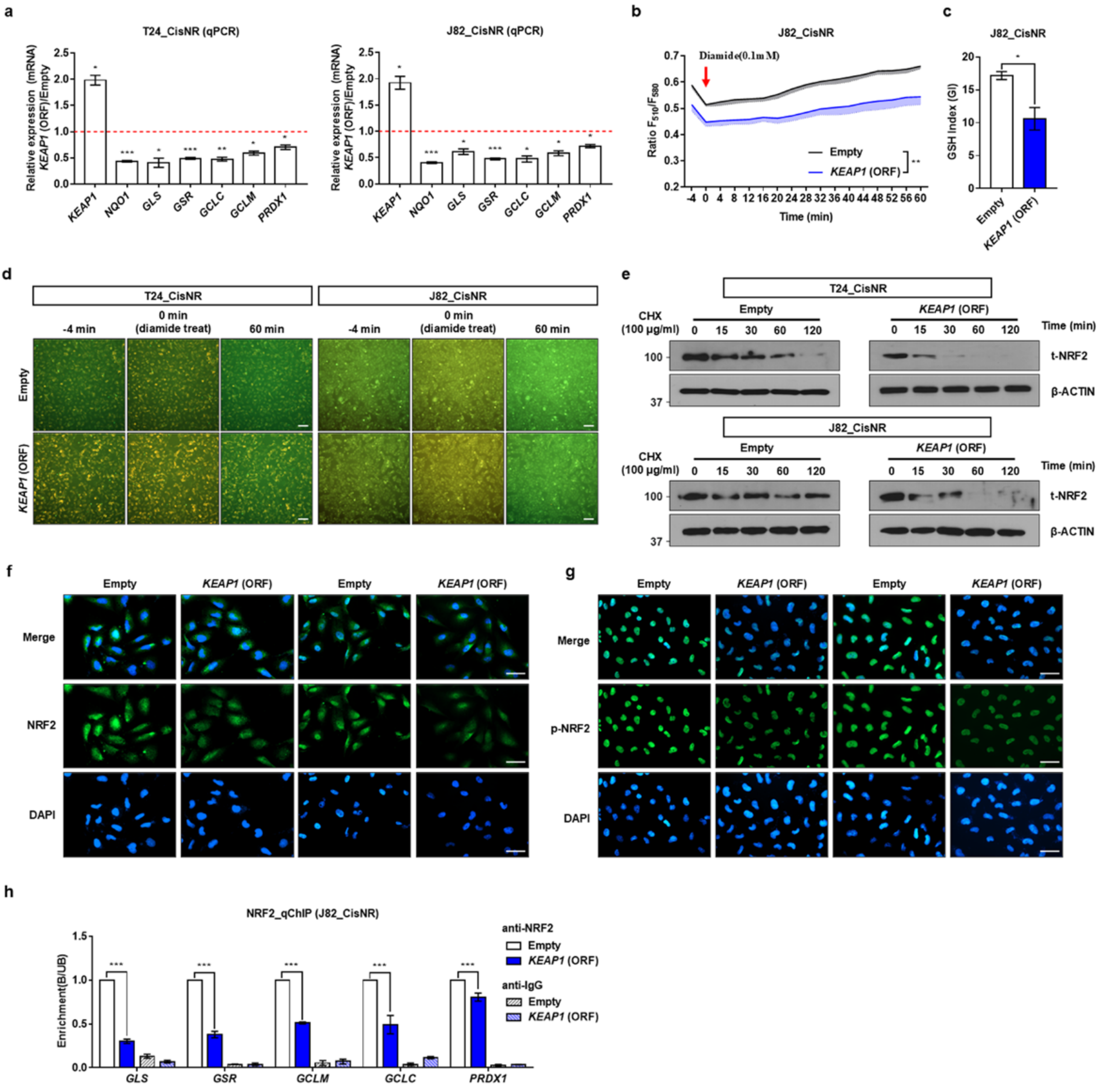

Supplementary Figure 12. KEAP1 suppresses NRF2 activity and GSH dynamics in cisplatin-resistant MIBC cells.

(a) Quantitative real-time PCR analysis of genes involved in GSH metabolism in *KEAP1*-overexpressing Cis_NR T24 and J82 MIBC cell lines (n = 5). Data represent fold changes

relative to empty vector control cells, which were normalized to 1 (indicated by the red dotted line). **(b–d)** FreSHtracer-based assessment of intracellular GSH dynamics in Cis_NR J82 cells overexpressing *KEAP1*. **(b)** Representative fluorescence ratio (FR) plots derived from F_{510} and F_{580} signals. **(c)** Quantification of the GSH index (GI), reflecting total GSH levels and recovery capacity ($n = 5$). **(d)** Representative fluorescence images visualizing F_{510} (GSH-bound) and F_{580} (unbound) emissions. **(e)** KEAP1-mediated regulation of NRF2 protein stability assessed by cycloheximide (CHX) chase assay in Cis_NR T24 (top) and J82 (bottom) cells. **(f and g)** Representative immunofluorescence images showing total **(f)** and phosphorylated **(g)** NRF2 protein expression (green) in *KEAP1*-overexpressing Cis_NR T24 cells. Nuclei were stained with DAPI (blue). Images were acquired at ×400 magnification; scale bars = 200 µm. **(h)** ChIP-qPCR assay for recruitment of NRF2 at the promoter regions of the indicated GSH dynamics target genes in *KEAP1*-overexpressing Cis_NR J82 cells ($n = 5$). Fold enrichment is shown relative to empty vector controls. Data are presented as mean ± SEM. Statistical significance was determined by unpaired Student's t-test **(c)** and one-way ANOVA **(a)** or two-way ANOVA **(b and h)** with Bonferroni post hoc test. $^*p < 0.05$, $^{**}p < 0.01$, $^{***}p < 0.001$. *Related to Fig. 5.*

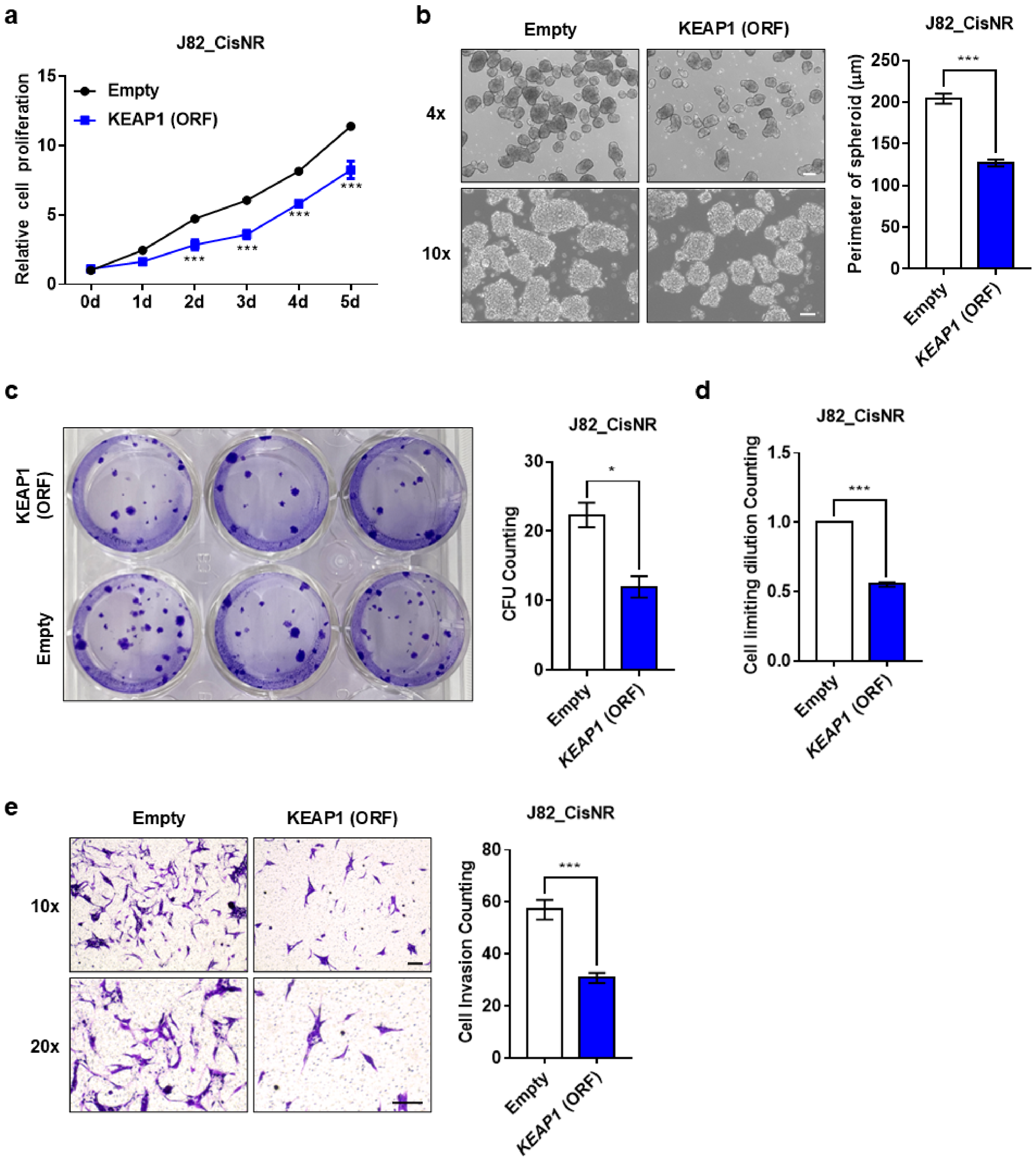

Supplementary Figure 13. KEAP1–NRF2 signaling modulates stemness features in

cisplatin-resistant J82 MIBC cells.

(a–e) Functional characterization of *KEAP1*-overexpressing Cis_NR J82 cells. **(a)** Proliferation assay ($n = 4$). **(b–e)** Stemness-associated phenotypes were evaluated by tumor sphere formation (**b**, $n = 45$), colony forming unit (CFU) efficiency (**c**, $n = 3$), clonogenicity via limiting dilution (**d**, $n = 3$), and Matrigel invasion assay (**e**, $n = 4$). Representative images of tumor spheres are shown at ×40 (top) and ×100 (bottom) magnification (scale bars = 200 μm); invasion images are shown at ×100 (top) and ×200 (bottom) magnification (scale bars = 100 μm). Quantitative data are presented as mean ± SEM. Statistical significance was assessed using unpaired two-tailed Student's t-test **(b–d)** or two-way ANOVA **(a)** with Bonferroni post hoc test. *$p < 0.05$, **$p < 0.01$, ***$p < 0.001$. *Related to Fig. 6.*

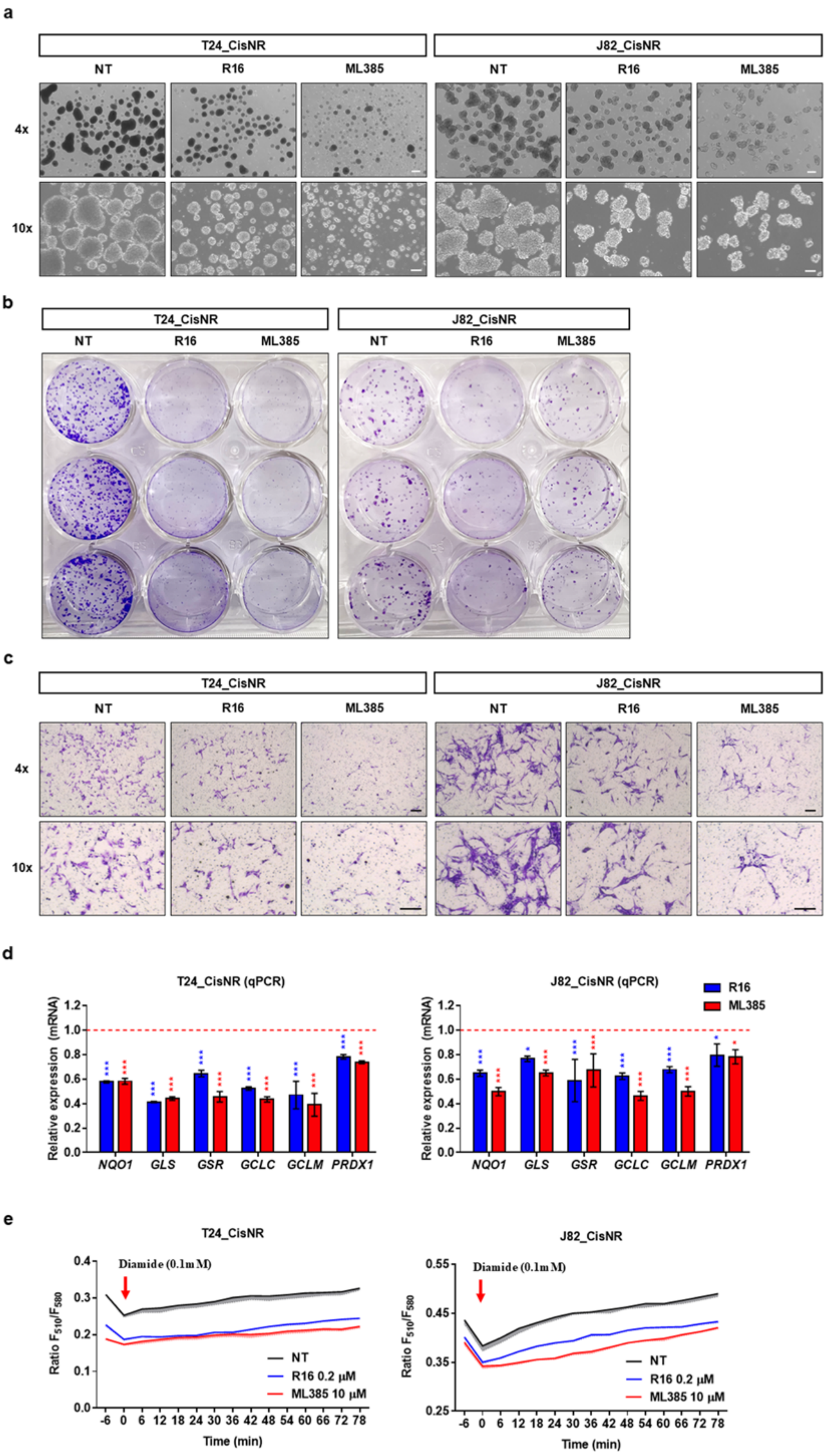
a
T24_CisNR
J82_CisNR
NT
R16
ML385
4x
10x
b
T24_CisNR
J82_CisNR
NT
R16
ML385
c
T24_CisNR
J82_CisNR
NT
R16
ML385
4x
10x
d
T24_CisNR (qPCR)
J82_CisNR (qPCR)
R16
ML385
Relative expression (mRNA)
0.0
0.2
0.4
0.6
0.8
1.0
1.2
NQO1
GLS
GSR
GCLC
GCLM
PRDX1
e
T24_CisNR
J82_CisNR
Diamide (0.1mM)
Ratio F510/F580
NT
R16 0.2 μM
ML385 10 μM
Time (min)
-6 0 6 12 18 24 30 36 42 48 54 60 66 72 78

Supplementary Figure 14. Inhibition of the KEAP1–NRF2 pathway impairs stemness features and redox capacity in cisplatin-resistant MIBC cells.

(a–c) Functional stemness feature assays in Cis_NR T24 and J82 cells following treatment with KEAP1–NRF2 inhibitors ML385 (10 μM) or R16 (0.2 μM) for 24 h. Representative images show tumor sphere formation **(a)**, colony forming capacity **(b)**, and Matrigel invasion activity **(c)** at ×40/×100 (sphere) or ×100/×200 (invasion) magnification; scale bars = 200 μm **(a)**, 100 μm **(c)**. **(d)** qPCR analysis of NRF2-dependent genes involved in GSH biosynthesis (*GCLM* and *GCLC*) and redox cycling (*GSR* and *PRDX1*) after ML385 or R16 treatment in Cis_NR T24 (left) and J82 (right) cells ($n = 4$). Data represent fold changes relative to non-treated (NT) control cells, which were normalized to 1 (indicated by the red dotted line). *NQO1*, NAD(P)H quinone dehydrogenase-1. **(e)** Assessment of intracellular GSH dynamics using the FreSHtracer system in Cis_NR T24 (left) and J82 (right) cells treated with KEAP1–NRF2 inhibitors. Quantification of GSH index (GI), reflecting total GSH levels and recovery capacity ($n = 3$). Data are presented as mean ± SEM. Statistical analyses were performed using two-way ANOVA with Bonferroni post hoc test. *$p < 0.05$, **$p < 0.01$, ***$p < 0.001$. *Related to Fig. 6.*

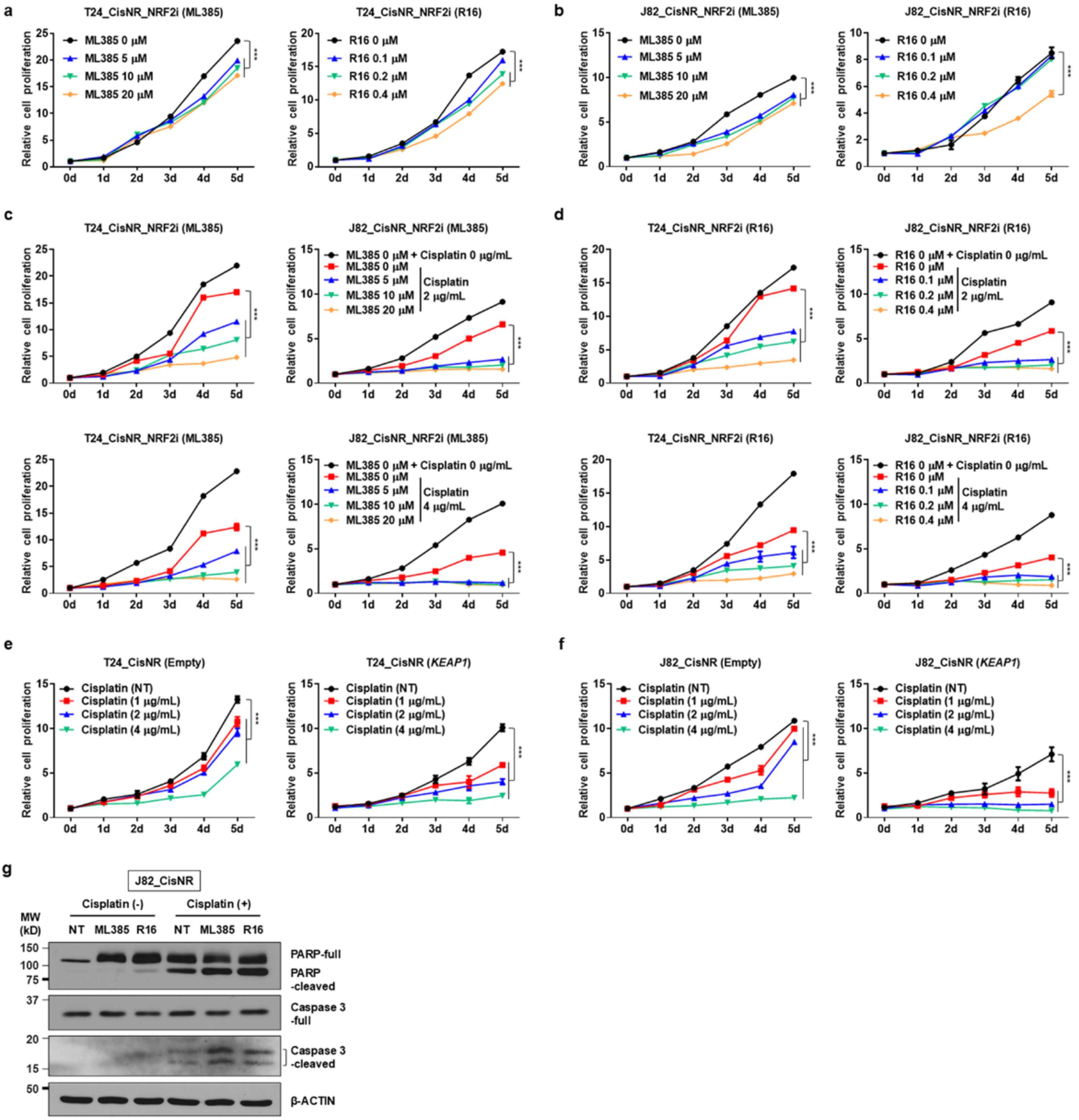

Supplementary Figure 15. In vitro evaluation of NRF2–KEAP1 pathway modulators for overcoming cisplatin resistance in MIBC cells.

(a and b) Dose-dependent cell viability in Cis_NR T24 and J82 MIBC cells following

treatment with NRF2–KEAP1 inhibitors ML385 **(a)** and R16 **(b)** as monotherapy ($n = 6$). **(c and d)** Cell viability assay of T24 and J82 Cis_NR MIBC cell lines treated with the indicated doses of the NRF2/KEAP1 inhibitors ML385 **(c)** and R16 **(d)** combined with the indicated concentration of cisplatin (n = 6). **(e and f)** Dose-dependent cell viability in Cis_NR T24 **(e)** and J82 **(f)** MIBC cells following overexpression of KEAP1 ($n = 6$). **(g)** Immunoblotting of cleaved caspase-3 and poly-(ADP-ribose) polymerase (PARP) in J82 Cis_NR MIBC cells treated with the indicated NRF2/KEAP1 pathway inhibitors alone or in combination with 1 μg/mL cisplatin. Quantitative data are expressed as the mean ± SEM; $*p < 0.05$, $***p < 0.001$, two-way ANOVA with Bonferroni post hoc test. The exact p-values and number of replicates are specified in the **Source datasets**. *Related to Fig. 7.*

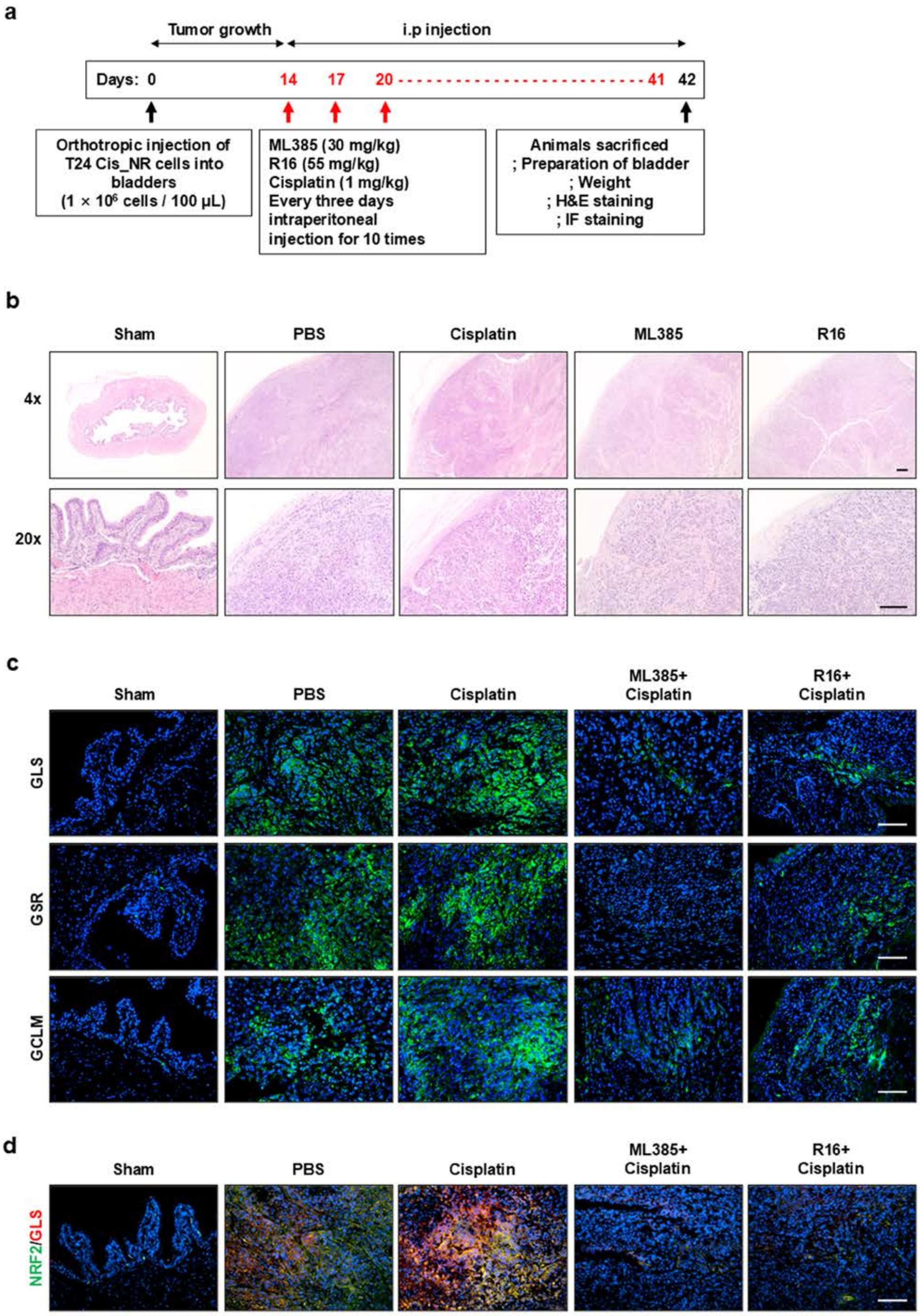
a
Tumor growth
i.p injection
Days: 0
14
17
20
41
42
Orthotropic injection of T24 Cis_NR cells into bladders (1 × 10⁶ cells / 100 μL)
ML385 (30 mg/kg)
R16 (55 mg/kg)
Cisplatin (1 mg/kg)
Every three days intraperitoneal injection for 10 times
Animals sacrificed
; Preparation of bladder
; Weight
; H&E staining
; IF staining
b
Sham
PBS
Cisplatin
ML385
R16
4x
20x
c
Sham
PBS
Cisplatin
ML385+ Cisplatin
R16+ Cisplatin
GLS
GSR
GCLM
d
Sham
PBS
Cisplatin
ML385+ Cisplatin
R16+ Cisplatin
NRF2/GLS

Supplementary Figure 16. ***In vivo*** **efficacy of combination therapy with cisplatin and KEAP1–NRF2 inhibitors**

(a) Schematic representation of the orthotopic xenograft model established by injecting 1.0×10^6 Cis_NR T24 human MIBC cells into the outer layer of the bladder wall of nonobese diabetic (NOD) Cg-*Prkdc*^{scid} *Il2rg*^{tm1Wjl}/SzJ (NSG) immunodeficient mice. Mice were treated via intraperitoneal injection with cisplatin alone, KEAP1–NRF2 modulators (ML385 or R16) alone, or the respective combinations, according to the indicated schedule. **(b)** Representative hematoxylin and eosin (H&E)–stained sections of bladder tissues from each treatment group, imaged at ×40 (upper panel; scale bars = 200 µm) and ×200 (lower panel; scale bars = 100 µm) magnification. **(c and d)** Immunofluorescence staining of tumor sections for proteins associated with the GSH dynamics (GLS, GSR, and GCLM) **(c)** and co-localization of NRF2 (green) and GLS (red) proteins **(d)** in residual tumor tissues following monotherapies or combination therapies. Representative merged images were acquired at ×200 magnification (scale bars = 100 µm). Nuclei were counterstained with DAPI (blue).

SUPPLEMENTARY TABLE LEGENDS; Provided by a separate Excel file.

Supplementary Table 1. Patient demographics of AMC NAC and PCT cohorts

Supplementary Table 2. Digital pathology datasets of the tumor compartment with clinical annotations

The table presents the expression levels of proteins in tumor epithelial cells, quantified using computational image analysis of IHC-stained TMAs from AMC NAC (*n* = 63) and PCT (n = 63) cohort patients. The expression levels represent the mean values calculated from three TMA cores per patient. Pathological responses in the AMC cohorts are indicated as "0" for non-response and "1" for response. * Additional information includes case numbers of patients used for RNA-seq analysis in the AMC cohort. Data were deposited in the NCBI Gene Expression Omnibus (GSE212810). ** Clinical annotations accompanying the expression data include patient demographics, tumor characteristics, treatment details, pathological findings, and survival outcomes. Sex (0, female; 1, male), TURBT Organ (1, UB; 5, combination/multiple organs in the urinary tract; 6, prostatic urethra), Tumor size (1, <3 cm; 2, ≥3 cm), Multiplicity (1, single; 2, multiple), Resection completeness (1, complete TURBT; 2, residual tumor after TURBT), clinical T stage (cT), 2004 WHO/ISUP Grade (2, low; 3, high grade including urothelial carcinoma *in situ*), Carcinoma *in situ* (CIS, 0, absence; 1, presence), Lymphovascular invasion (LVI, 0, absence; 1, presence), NAC regimen (1, GP; 2, GCb; 3, DDMVAC), NAC cycles, Procedure after NAC (3, radical cystectomy/nephrectomy/ureterectomy; 4, partial cystectomy), ypT stage after NAC (0, noninvasive or CIS; 1, subepithelial connective tissue invasion; 2, musclularis propria invasion; 3, perivesical fat invasion; 4, other organ involvement such as direct invasion to prostate, perirenal adipose tissue, and mesentery; 777, no residual tumor), lymph node metastasis in curative surgery specimens after NAC (0, absence;

1, presence; 999, not determined), Follow-up period (month), Progression (0, absence; 1, presence), Progression-free survival (month), Death (0, alive; 1, dead).

Supplementary Table 3. Digital pathology datasets of the stromal compartment with clinical annotations

The Supplementary Table ummarizes the protein expression levels in the stromal compartment, as determined by computational image analysis of IHC-stained TMAs from AMC NAC (*n* = 63) and PCT (*n* = 63) cohort patients. The protein expression data are presented alongside relevant clinical annotations for each patient.

Supplementary Table 4. Gene classifiers for transcriptome modeling in the AMC discovery cohort

The table lists the genes included in the gene classifiers used for machine learning-based transcriptome analysis in the AMC discovery cohort. The classifiers consist of: i) five AMC classifiers, including two data-driven classifiers and three classifiers representing molecular pathways associated with NAC response, as previously reported (Kim et al., 2023), ii) seven MDA_MVAC classifiers (Choi et al., 2014), and iii) five TCGA 2017 classifiers (Kim et al., 2019) including signature biomarkers for MIBC molecular subtyping and for predicting responses to NAC and immune checkpoint inhibition.

Supplementary Table 5. Gene classifiers after the gene classifier reduction process

The table lists the genes included in the gene classifiers after the gene classifier reduction process, which resulted in the selection of fewer than six optimal genes for each classifier.

Supplementary Table 6. Gene classifiers for modeling of multi-cohort cross-validation of

the transcriptome datasets

The table lists the genes included in the classifiers used for multi-cohort cross-validation, including 17 gene classifiers and transcriptome datasets from the AMC cohort and three independent external cohorts. The external cohorts included publicly available gene expression profiling datasets derived from pre-NAC TURBT specimens along with records of pathological responses to NAC. For each classifier, the top 10 key variables were identified by random forest modeling for each dataset. Additionally, union classifiers were developed by combining the top 10 genes from each classifier, using each cohort as a training set.

Supplementary Table 7. Key features identified after gene classifier reduction

The table lists the key feature genes identified following the gene classifier reduction process applied to the union AMC data-driven classifiers derived from multi-cohort cross-validation of transcriptome datasets. The key features are ranked according to the coefficient values in the logistic regression model or the importance scores in the decision tree model.

Supplementary Table 8. Correlation between the expression of six key predictive markers and clinicopathological features in the AMC validation cohort

Association between the expression levels of six key predictive genes identified by decision tree modeling (*PCDHB9*, *HCFC1R1*, *NT5E*, *TNFAIP8*, *AFAP1*, and *POU2F2*) and clinicopathological characteristics in the AMC validation cohort. Patients were stratified into high- and low-expression groups for each gene using optimized cutoffs. Clinicopathological variables analyzed include age, sex, tumor size, multiplicity, histologic grade, lymphovascular invasion, presence of carcinoma in situ, clinical T category (cT), lymph node metastasis, and disease progression. Overall survival was additionally evaluated according to gene expression status.

Supplementary Table 9. Transcript levels of putative biomarker genes across consensus gene expression subtypes

Expression levels of 50 biomarker genes identified through multi-cohort transcriptome machine learning analysis, extracted from QuantSeq 3' mRNA-Seq datasets for each patient in the AMC validation cohort, stratified according to the consensus gene expression subtypes.

Supplementary Table 10. Univariate analysis of the expression of the 33 putative proteins at the tumorous epithelial cells and in the stromal compartment

SUPPLEMENTARY MATERIALS AND METHODS

Real-time live-cell imaging of GSH recovery capacity (GRC)

Real-time monitoring of intracellular GSH dynamics in individual live cells were assessed as described previously [1-3]. This GRC assay enables nondestructive, image-based, high-throughput quantification of both the qualitative and quantitative aspects of GSH recovery in live human MIBC cells under various experimental conditions. The assay is based on FreSHtracer (**F**luorescent **re**al-time **thiol tracer**; Cell2in, Inc., Seoul, Korea), a reversible, cell-permeable chemical probe that exhibits a distinct spectral shift upon reaction with GSH [1]. Specifically, GSH binding induces a shift in the probe's absorption maximum from 520 nm to 430 nm, resulting in decreased fluorescence at 580 nm (F580, λ_ex = 520 nm) and increased fluorescence at 510 nm (F510, λ_ex = 430 nm). Fluorescence ratio (FR) values were calculated by measuring emissions at 510 and 580 nm following respective excitations.

Live-cell imaging was conducted in real time using the FreSHcell Q system (Cell2in), an automated high-content platform equipped with an sCMOS camera, at ×200 or ×400 magnification, according to the manufacturer's instructions. Fluorescence images were analyzed using NIS-Elements AR software (Nikon, Tokyo, Japan). Image preprocessing involved rolling ball background subtraction and shading correction to normalize illumination. Individual cells were segmented using an integrated AI module. For each cell, the GSH index (GI) was determined from baseline FR values, and the GRC was derived from the slope of FR changes following diamide treatment. All quantifications are included in **Source datasets**.

Gene expression analysis

For gene expression analysis at the transcript level, total RNA (50 ng) from human MIBC cell lines was reverse transcribed using Taqman Reverse Transcription Reagents (Applied

Biosystems, Foster City, CA, USA), and threshold cycles (Ct) were determined by quantitative PCR. To validate gene expression in AMC cohort patients, RNA (50 ng) from laser capture microdissection (LCMD) samples was reverse transcribed and amplified using the SMARTer Stranded Total RNA-Seq Kit (Takara, Kusatsu, Shiga, Japan), and cDNA libraries were used for qPCR. Relative gene expression was calculated using the $2^{-\Delta\Delta Ct}$ method, with human β2-microglobulin (*B2M*) as the control, as previously described [3,4]. Primers used in the qPCR assay are listed in the key resources table.

Western blot analysis

Whole-cell lysates (30 μg per lane) were prepared using RIPA lysis buffer (Santa Cruz Biotechnology, Santa Cruz, CA, USA) supplemented with protease and phosphatase inhibitor cocktails (Roche, Indianapolis, IN, USA). Proteins were separated on 12% SDS–PAGE gels and transferred to PVDF membranes (GE Healthcare, Chicago, IL, USA). Membranes were probed with primary antibodies specific to the indicated target proteins, as detailed in the key resources table. Signal detection was performed using standard chemiluminescence protocols. Uncropped blot western blot results are provided in **Source datasets**.

Chromatin immunoprecipitation (ChIP) assay

ChIP assays were performed using the Magna ChIP G kit (Millipore, Billerica, MA, USA) following the manufacturer's protocol and as previously described [5,6]. Briefly, chromatin from cross-linked cell lysates (1×10^7 cells) was sheared using a Bioruptor Plus sonicator (Diagenode, Denville, NJ, USA) with four 20-second pulses interspersed with 30-second intervals on ice. Immunoprecipitation was carried out using 3 μg of ChIP-grade anti-NRF2 antibody (ab62352, Abcam, Cambridge, UK) or control rabbit IgG (2729S, Cell Signaling Technology, Danvers, MA, USA). Enrichment of NRF2 binding was quantified as the ratio of

bound to unbound amplicon fractions and is presented as mean ± SEM from four independent experiments. Primer sequences used for the ChIP assay are listed in the key resources table.

Cell proliferation and apoptosis assay

Cell proliferation was assessed using the MTT assay (Sigma-Aldrich, Burlington, MA, USA) according to the manufacturer's instructions. Apoptosis was evaluated via Annexin V–fluorescein isothiocyanate (FITC) and propidium iodide (PI) staining. Cells were harvested by trypsinization, washed with PBS, and resuspended in Annexin V binding buffer (10 mM HEPES, pH 7.4, 140 mM NaCl, 2.5 mM $CaCl^2$). Cells were then incubated with Annexin V FITC and PI, and stained populations were quantified using flow cytometry (Beckman Coulter, Brea, CA, USA).

Tumor sphere formation and limiting dilution assays

For tumor sphere formation, human MIBC cells were resuspended as single-cell suspensions in a 1:1 mixture of serum-free Keratinocyte Growth Medium (Gibco, Waltham, MA, USA) and Growth Factor Reduced Matrigel (BD Biosciences, Mountain View, CA, USA), and seeded into Ultra-Low Attachment plates (Costar, Corning, NY, USA). Tumor sphere size was measured seven days after seeding. For quantification, perimeters of spheres were measured in eight randomly selected fields per group using ImageJ software (NIH, Bethesda, MD, USA).

For the limiting dilution assay, the MIBC cells were plated at a density of one cell per well in 96-well plates containing 50 μL of culture medium. Medium was replenished every two days, and colony formation was assessed ten days post-seeding. The number of colonies per group was used for quantification.

***In vitro* cell invasion assay**

Cell invasion was assessed using Transwell inserts (8.0 µm pore size, polycarbonate membrane; Corning Inc.) precoated with Matrigel (BD Biosciences) diluted 1:5 in serum-free medium. The BC cells (2×10^4 cells/well) were seeded into the upper chambers in 100 µL of serum-free DMEM, while the lower chambers were filled with culture medium containing 3% FBS as a chemoattractant. After 24 hours of incubation at 37 °C in a 5% CO_2 atmosphere, non-invading cells were removed from the upper surface, and cells that had migrated to the underside of the membrane were fixed, stained, and counted. Quantification was performed by counting cells in three randomly selected microscopic fields per insert at ×200 magnification.

Orthotopic xenograft MIBC animal model

Eight-week-old male NOD/ShiLtJ-$Prkdc^{em1AMC}Il2rg^{em1AMC}$ (NSGA) mice (GEM Biosciences Inc., Cheongju, Korea) were acclimated for one week under standard conditions at the Asan Medical Center animal facility. For orthotopic xenograft modeling, 1.0×10^6 cisplatin-nonresponsive (Cis_NR) T24 MIBC cells suspended in 100 µL PBS were directly injected into the serosal layer of the anterior bladder wall and dome using a 500 µm Hamilton syringe fitted with a 26-gauge needle, following established procedures, as previously reported [3,6,7]. Three weeks after cell implantation, mice received intraperitoneal administration of cisplatin (1 mg/kg), NRF2 inhibitors (ML385 at 30 mg/kg or R16 at 55 mg/kg), or combination treatments. Injections were performed in ten cycles at three-day intervals. Mice were monitored every other day for 45 days following tumor cell implantation, assessing general health and injection sites. At study endpoint, bladders were harvested for tumor size measurement, histopathological evaluation, and immunofluorescence analysis. Mice were randomly assigned to experimental groups ($n = 5$ per group). Cell implantation, treatment administration, and outcome evaluations were conducted in a randomized order. Investigators responsible for tumor size measurements and histological analyses were blinded to the treatment conditions.

Immunocytochemistry analysis and histological examinations

For immunocytochemistry, human MIBC cells were fixed in 4% paraformaldehyde (Sigma-Aldrich) and stained with anti-t-NRF2 (ab62352, Abcam), anti-p-NRF2 (ab76026, Abcam), anti-KEAP1 (10503-2-AP, PROTEIN TECH) antibodies followed by incubation with Alexa Fluor 488–conjugated anti-rabbit secondary antibody (A11008, Thermo Fisher Scientific, Waltham, MA, USA). Fluorescent images were acquired using a Zeiss LSM710 confocal microscope (Carl Zeiss, Munich, Germany).

For histological analysis of xenograft tissues, bladders were fixed in 4% paraformaldehyde for 24 h, cryoprotected in 30% sucrose, and embedded for sectioning. Serial 20 μm-thick sections were prepared using a cryostat (Leica, Lussloch, Germany) and stained with hematoxylin and eosin (H&E). For immunofluorescence (IF) staining, sections were incubated with primary antibodies (listed in the key resources table), followed by Alexa Fluor 488–conjugated anti-mouse or anti-rabbit antibodies (A11001, A11008), or Alexa Fluor 546–conjugated anti-mouse antibody (A11060; Thermo Fisher Scientific). Nuclei were counterstained with 4′,6-diamino-2-phenylindole (DAPI; Sigma-Aldrich), and stained tissues were visualized using an EVOS FL Color Imaging System (Life Technologies, Carlsbad, CA, USA).

KEY RESOURCES TABLE

REAGENT or RESOURCE	SOURCE	IDENTIFIER
Antibodies		
ADA	Santacruz	Cat# sc-376889 RRID:AB 3674574
AFAP1	NOVUS	Cat# NBP2-46498 RRID:AB_3310472
Caspase-3	Cell signaling	Cat# 9662S RRID:AB 331439
CARD16	NOVUS	Cat# NBP2-30778 RRID:AB 3277985
CD44v6	Abcam	Cat# ab78960; RRID:AB_1603730
CD73	Abcam	Cat# ab133582 RRID:AB 3674653
Cleaved caspase-3	Cell signaling	Cat# 9661 RRID:AB 2341188
DNMT3L	Abcam	Cat# ab194094 RRID:AB 2783649
DPH2	Origene	Cat# TA504834 RRID:AB 2622634
EGR2	NOVUS	Cat# NB110-59723 RRID:AB 892428
FYB1	NOVUS	Cat# NBP2-76882 RRID:AB 3674654
GADD45B	Origene	Cat# TA346618 RRID:AB 3674575
GCLC	Abcam	Cat# ab190685; RRID:AB_2889925
GCLM	Abcam	Cat# ab81445; RRID:AB_1860504
GLS	Abcam	Cat# ab156876, RRID:AB_2721038
GPX2	Abcam	Cat# ab137431 RRID:AB 3674655
GPX4	Abcam	Cat# ab125066 RRID:AB 10973901
GSR	Abcam	Cat# ab128933; RRID:AB_11142591
HCFC1R1	NOVUS	Cat# NBP2-31788 RRID:AB 3278824
KCTD14	NOVUS	Cat# NBP2-00470 RRID:AB 3245030
KEAP1	PROTEIN TECH	Cat# 10503-2-AP RRID:AB 2132625
KRT14	DAKO	Cat# 314M-14; RRID:AB_1159418
MTCH1	Invitrogen	Cat# PA5-42964 RRID:AB 2576880
MUC16	Santacruz	Cat# sc-365002 RRID:AB 10708400
MYC	Cell signaling	Cat# 5605S RRID:AB 1903938
NOTUM	NOVUS	Cat# NBP1-77060 RRID:AB 11025655
PARP	Cell signaling	Cat# 9542S

		RRID:AB 2160739
PCDHB9	Aviva	Cat# ARP9450_P050 RRID:AB 3674656
PD-1	Roche	Cat# 760-4895 RRID:AB 3674657
PD-L1	Roche	Cat# 790-4905 RRID:AB 2819099
POU2F2	NOVUS	Cat# NBP2-75732 RRID:AB 3401824
PPIL2	NOVUS	Cat# NBP1-85360 RRID:AB 11043074
PRDX1	Abcam	Cat# ab15571; RRID:AB_2170316
PTPN12	Sigma-Aldrich	Cat# HPA007097 RRID:AB 1079689
RFX7	NOVUS	Cat# NBP1-83705 RRID:AB 11037202
SIRT6	Cell signaling	Cat# 12486 RRID:AB 2636969
SLC15A3	NOVUS	Cat# NBP1-92422 RRID:AB 11033984
TF	Santacruz	Cat# sc-374441 RRID:AB 11008609
TFEB	Cell signaling	Cat# 37785 RRID:AB 2799119
TNFAIP8	PROTEIN TECH	Cat# 15790-1-AP RRID:AB 10792805
p-NRF2	Abcam	Cat# ab76026 RRID:AB 1524049
t-NRF2	Abcam	Cat# ab62352 RRID:AB 944418
USP2	NOVUS	Cat# NBP1-86883 RRID:AB 11016818
ZBTB12	NOVUS	Cat# H00221527-B01P RRID:AB 1581989
Alexa488 anti-rabbit	Thermo Fisher Scientific	Cat# A-11008; RRID:AB_143165
Alexa488 anti-mouse	Thermo Fisher Scientific	Cat# A-11001; RRID:AB_2534069
Alexa546 anti-mouse	Thermo Fisher Scientific	Cat# A-11060; RRID:AB_2534107
Normal Rabbit IgG	Cell Signaling Technology	Cat# 2729; RRID:AB_1031062
DAPI	Sigma-Aldrich	Cat# D9542; RRID: NA
Biological Samples		
Bladder cancer patient samples	This study	IRB; AMC (2020-0064)
Chemicals, Peptides, and Recombinant Proteins		
Cisplatin (cis-Diammineplatinum(II) dichloride)	Sigma-Aldrich	P4394
ML385	MedChemExpress	HY-100523
R16	MedChemExpress	HY-149508
RPMI1640 medium	HyClone	SH30027.01

MEM medium	Gibco	11095-080
FBS	Hyclone	SH30084.03
Penicillin/streptomycin solution	Corning	30-002-CI
Protease inhibitor cocktails	Roche	05892791001
Phosphatase inhibitor cocktails	Roche	04906837001
Sodium butylate	Millipore	19-137
RNeasy purification kit	QIAGEN	74104
Critical Commercial Assays		
SMARTer Stranded Total RNA-Seq Kit v2	Takara	634411
Taqman Reverse Transcription Reagents	Applied Biosystems	N8080234
ultraView Universal DAB detection kit	Roche	760-500
Cell Viability Assay Kit EZ-CYTOX	Daeil Lab	EZ-3000
FITC Annexin V Apoptosis Detection Kit I	BD Biosciences	556547
QuantSeq 3' mRNA-Seq Library Prep Kit	Lexogen	N/A
Deposited Data		
Transcriptome datasets for AMC cohort	Kim et al., 2023	GSE212810
Transcriptome datasets for MDA MVAC cohort	Choi et al., 2014	GSE48277
Transcriptome datasets for MDA DDMVAC cohort	McConkey et al., 20 16	GSE69795
Transcriptome datasets for NAC metadata cohort	Seiler et al., 2017	GSE87304
Experimental Models: Cell Lines		
Cisplatin resistance T24	Lee et al., 2003	N/A
Cisplatin resistance J82	University of Kent (T he Resistant Cancer Cell Line collection)	N/A
Cisplatin resistance KU19-19	University of Kent (T he Resistant Cancer Cell Line collection)	N/A
293FT	Thermo Fisher Scientific	Cat# R70007
Experimental models: Organisms/strains		
NOD/ShiLtJ-Prkdcem1AMCIl2rgem1AMC (NSGA)	JA BIO	Cat# NSG_A
Oligonucleotides		
Human AFAP1_F (GCCATCGAGCCCAAGTCA)	This study	N/A
Human AFAP1_R (GGAGATGGGCGAGTTTTCC)	This study	N/A
Human AOX1_F (GCATGTGACTCCTACTTGGCT)	This study	N/A
Human AOX1_R (ACAAGAGGAAGGTGATAGCAGA)	This study	N/A
Human B2MG_F (GAGGGCTGGCAACTTAGAGG)	This study	N/A
Human B2MG_R (ACAAGCTTTGAGTGCAAGAGA)	This study	N/A
Human CADM4_F (ACTTTGATGGGGGCAGTGAG)	This study	N/A
Human CADM4_R (TGGAGGAGAGATGGGGAAGG)	This study	N/A
Human CD53_F (TGGACCATTGTCACAACCCT)	This study	N/A
Human CD53_R (TGAGATCTTGCCCTTTGGAGA)	This study	N/A
Human CDK6_F (GGTGTTTCTGTTGCAGTGGC)	This study	N/A

Human CDK6_R (GCACCCAGTAAGACATCCAGT)	This study	N/A
Human COX17_F (AGGAGAACGGCAAGCTTCAA)	This study	N/A
Human COX17_R (TCACACAGCAGACCACCATT)	This study	N/A
Human CRMP1_F (ACATCGCCAAGGACTGACTG)	This study	N/A
Human CRMP1_R (ACAGACCAGAAGACAGCACG)	This study	N/A
Human EGR2_F (AGCCCTTCGCCTGTGACTAC)	This study	N/A
Human EGR2_R (GCACTGCTTTTCCGCTCTTTC)	This study	N/A
Human GCLC_F (GGAGGAAACCAAGCGCCAT)	This study	N/A
Human GCLC_R (CTTGACGGCGTGGTAGATGT)	This study	N/A
Human GCLM_F (TGTCTTGGAATGCACTGTATCTC)	This study	N/A
Human GCLM_R (CCCAGTAAGGCTGTAAATGCTC)	This study	N/A
Human GLS_F (GCATTCCTGTGGCATGTATGACTT)	This study	N/A
Human GLS_R (CCCCCAGCAACTCCAGATTT)	This study	N/A
Human GPX2_F (GACTTCACCCAGCTCAACGA)	This study	N/A
Human GPX2_R (ATGCTCGTTCTGCCCATTCA)	This study	N/A
Human HSPB7_F (TCCCATCTGGGATAGGGACC)	This study	N/A
Human HSPB7_R (CACTGACCAGAGAGGCATGG)	This study	N/A
Human KCTD14_F (GATCCCCTTTGCGAGATGCT)	This study	N/A
Human KCTD14_R (AGGTCAGTCTTTTGTACCTCCA)	This study	N/A
Human KEAP1_F (TGGAAAGAGCAGGCTTCCAG)	This study	N/A
Human KEAP1_R (CCCCTCCCAGGTATCCAAGA)	This study	N/A
Human MAD2L1_F (CCTGACTCCCCAACAAGCAT)	This study	N/A
Human MAD2L1_R (CCTCTGTATTTGGTTCCTCCCA)	This study	N/A
Human MYC_F (CGTCTCCACACATCAGCACAA)	This study	N/A
Human MYC_R (CACTGTCCAACTTGACCCTCTTG)	This study	N/A
Human NRF2_F (TGAGGATTCCTTCAGCAGCAT)	This study	N/A
Human NRF2_R (GACTGTGGCATCTGAATTTAATGAGT)	This study	N/A
Human NQO1_F (GGCTAGGTATCATTCAACTCTCCAA)	This study	N/A
Human NQO1_R (CTTCTCTGAGCAATTCCCTTCTG)	This study	N/A
Human PCDHB9_F (TGCAGGTCTCCGACGTCAAT)	This study	N/A
Human PCDHB9_R (TAGGTGACCTGGGCGTTGGT)	This study	N/A
Human POU2F2_F (CGTCCGCTTCGCCTTAGAGA)	This study	N/A
Human POU2F2_R (CAGACGCGGATCACTTCCTT)	This study	N/A
Human PRDX1_F (CATTCCTTTGGTATCAGACCCG)	This study	N/A
Human PRDX1_R (CCCTGAACGAGATGCCTTCAT)	This study	N/A
Human PSD2_F (TTCCCCATGCTGCCTCAAAA)	This study	N/A
Human PSD2_R (CCCATGCTCAGGAAGCACAT)	This study	N/A
Human SIRT6_F (CCCACGGAGTCTGGACCAT)	This study	N/A
Human SIRT6_R (CTCTGCCAGTTTGTCCCTG)	This study	N/A
Human TF_F (GGCGCTTCAGGCACTACAA)	This study	N/A
Human TF_R (TTGATTGACGGGTTTGGGTTC)	This study	N/A

Human THBS2_F (CAATGGTGTGGGAGTGACGA)	This study	N/A
Human THBS2_R (AAGTGCAGGGTTTCAGTGGT)	This study	N/A
Human ZNF493_F (TTTGTTGCTGCATCAGAGGT)	This study	N/A
Human ZNF493_R (ACCACAAAGAACCTCTCCACT)	This study	N/A
Human GLS_qChIP_F (AGAGCCGAGAGAAATTTGACT)	This study	N/A
Human GLS_qChIP_R (ATTGCCGCGACCGGTTCTCTT)	This study	N/A
Human GSR_qChIP_F (TCTTTCAAAGCCCCTACCTCTCT)	This study	N/A
Human GSR_qChIP_R (CATGATTGCCAAGTCACAGTGA)	This study	N/A
Human GCLM_qChIP_F (TGCATAAGCCTACTGGATCAGAGT)	This study	N/A
Human GCLM_qChIP_R (TCATGCAGGTCAAAAAGGAAGTAA)	This study	N/A
Human GCLC_qChIP_F (TGGTGCACTGGCTTCTTCCT)	This study	N/A
Human GCLC_qChIP_R (CGAGCTAGCGGACGCAAAG)	This study	N/A
Human PRDX1_qChIP_F (GCCCAACTCAGTCTCCCAAA)	This study	N/A
Human PRDX1_qChIP_R (TGCGCCCAGTGGTCTTG)	This study	N/A
Recombinant DNA		
ORF of human *KEAP1* in pEZ CMV	GeneCopoeia	EX-M0487-Lv105
Software and Algorithms		
GraphPad Prism 7.0	GraphPad Software	N/A
SPSS version 21.0	IBM	N/A
DAVID Bioinformatics	National Institutes of Health	N/A
QuPath	Bankhead et al., 2017	N/A
Source codes for machine learning models based on transcriptome datasets	This study	https://doi.org/10.5281/zenodo.14603074
Source codes for machine learning models based on computational pathology datasets	This study	https://doi.org/10.5281/zenodo.14603169
Other		
BenchMark XT Automated IHC/ISH slide staining system	Roche	N/A
Vectra 3.0 Automated Quantitative Pathology Imaging System	Perkin-Elmer	CLS142568
Pannoramic 250 Flash slide scanner	3D HISTECH	N/A
Clinical annotations	This study	Supplementary Table 6 – 9
BD FACSCanto II flow cytometer	BD Biosciences	N/A
Cryostat	Leica	RM2125 RTS

Uncropped WB

Fig. 5a

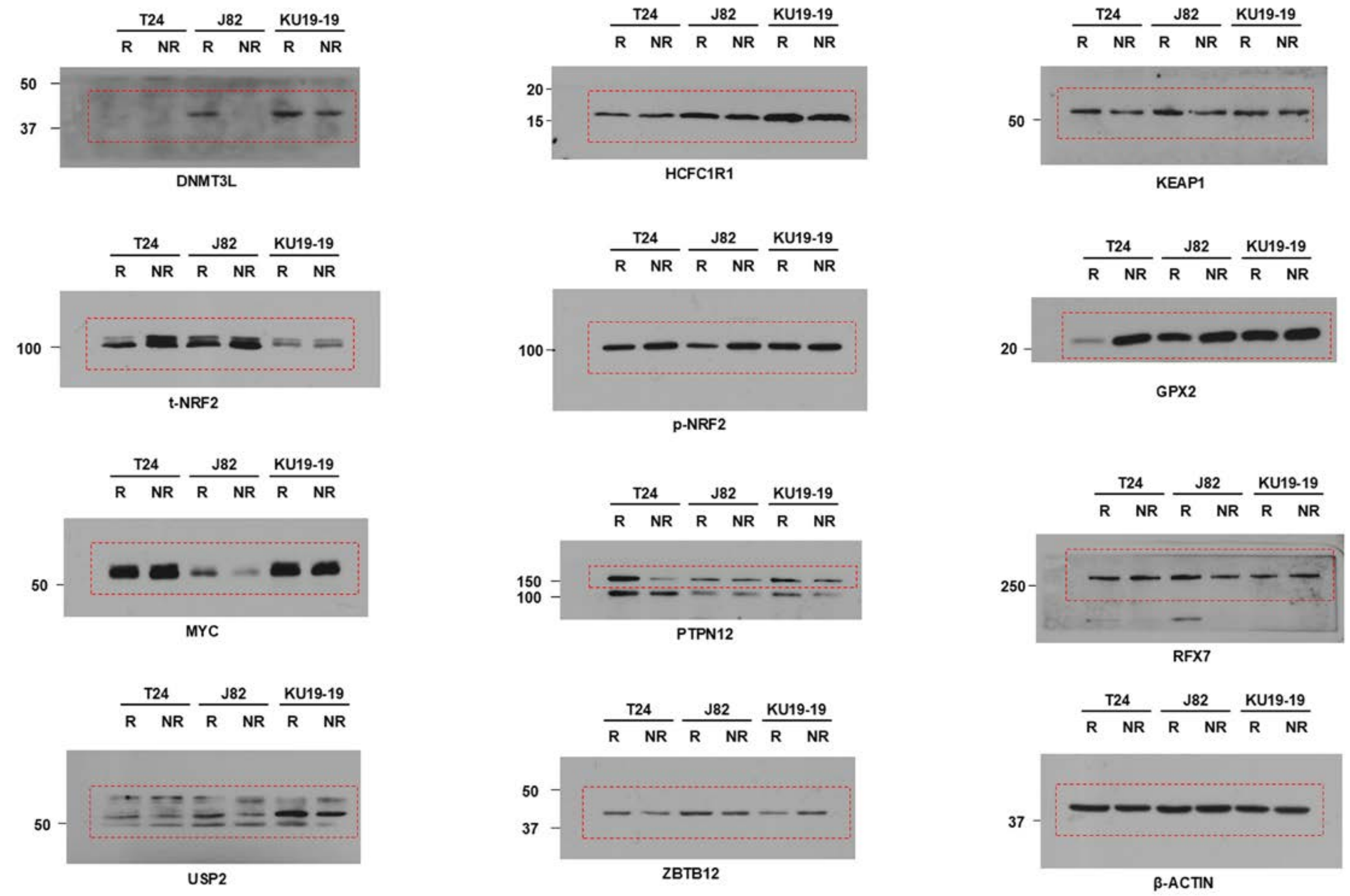

Fig. 5e

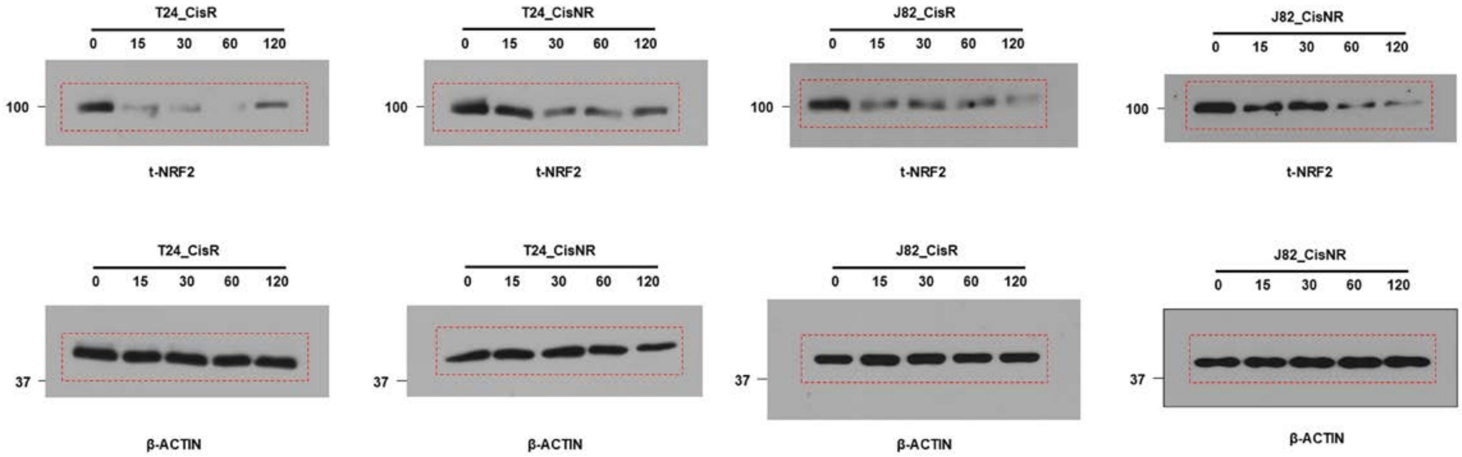

Fig. 5f

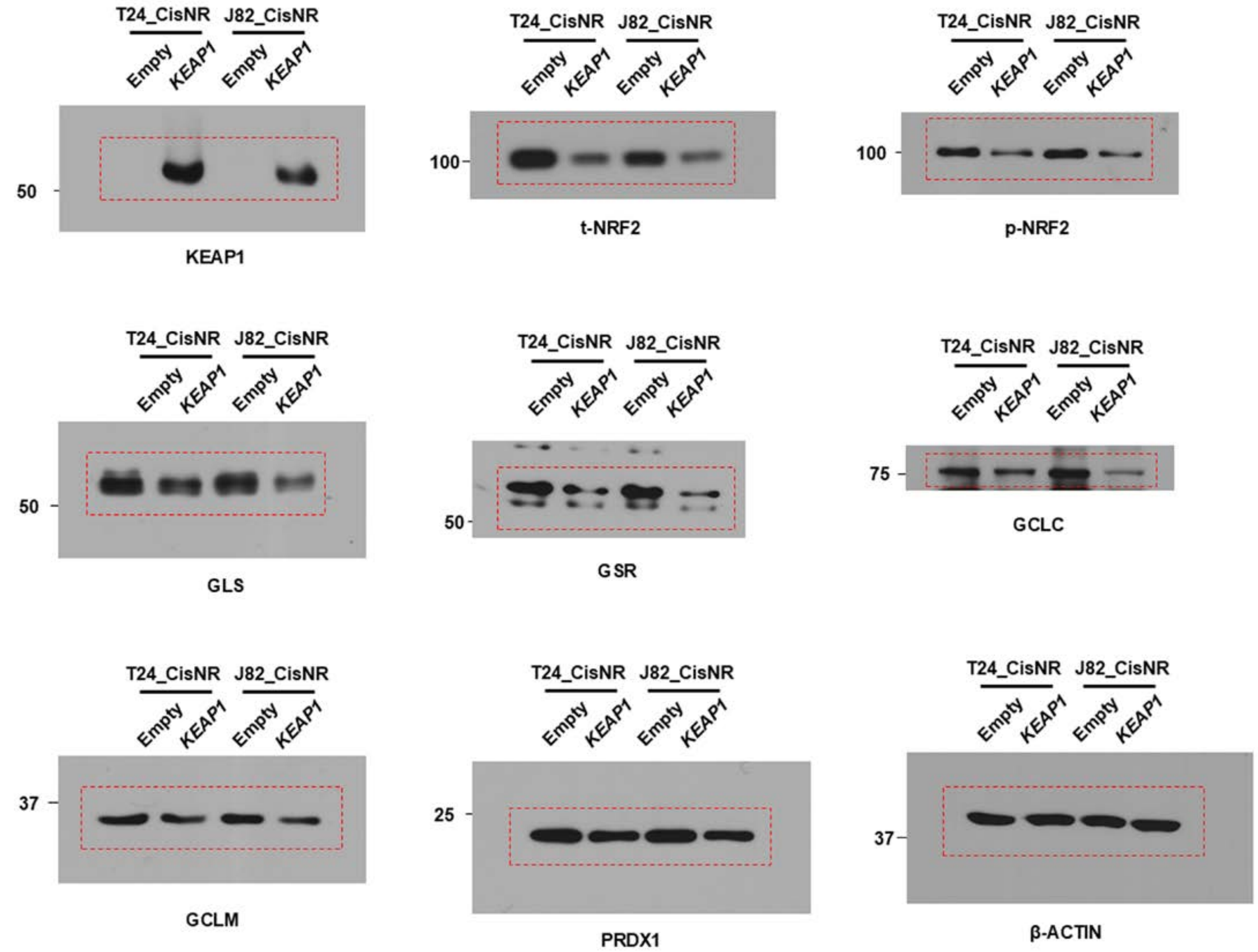

T24_CisNR
J82_CisNR
Empty
KEAP1
Empty
KEAP1
50
KEAP1
100
t-NRF2
100
p-NRF2
50
GLS
50
GSR
75
GCLC
37
GCLM
25
PRDX1
37
β-ACTIN

Fig. 6j

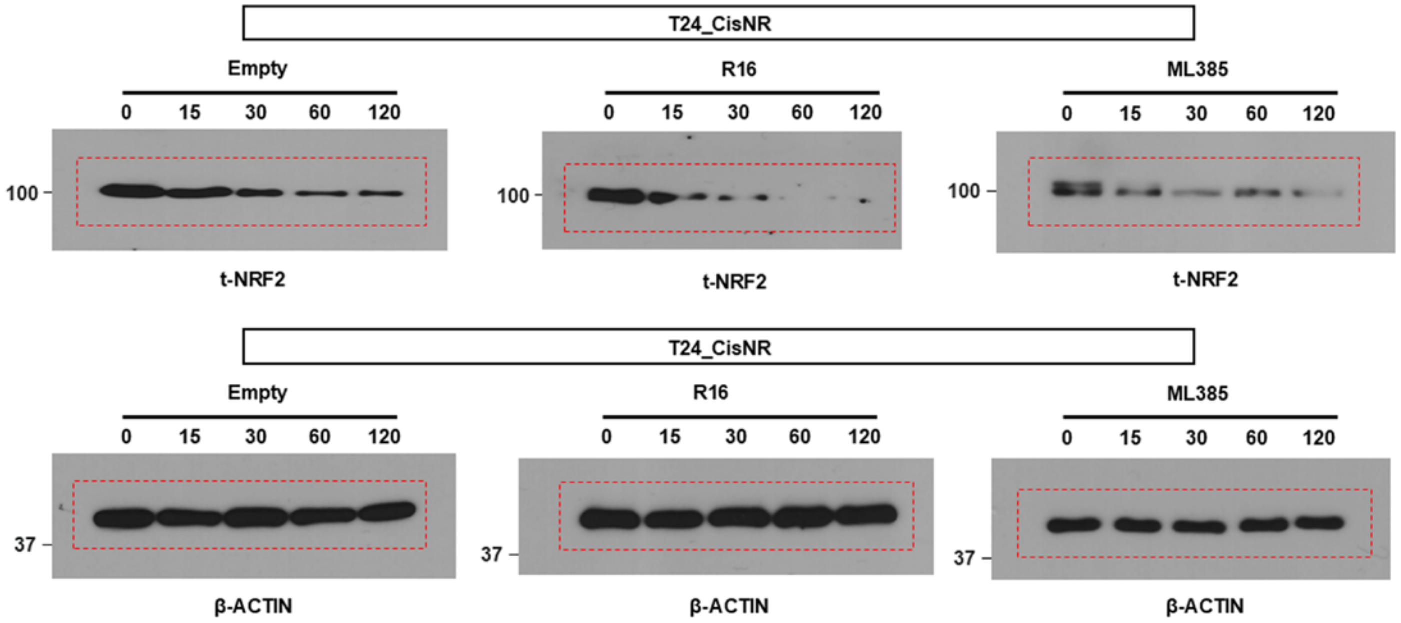
T24_CisNR
Empty
0 15 30 60 120
100
t-NRF2
R16
0 15 30 60 120
100
t-NRF2
ML385
0 15 30 60 120
100
t-NRF2
T24_CisNR
Empty
0 15 30 60 120
37
β-ACTIN
R16
0 15 30 60 120
37
β-ACTIN
ML385
0 15 30 60 120
37
β-ACTIN

Fig. 6k

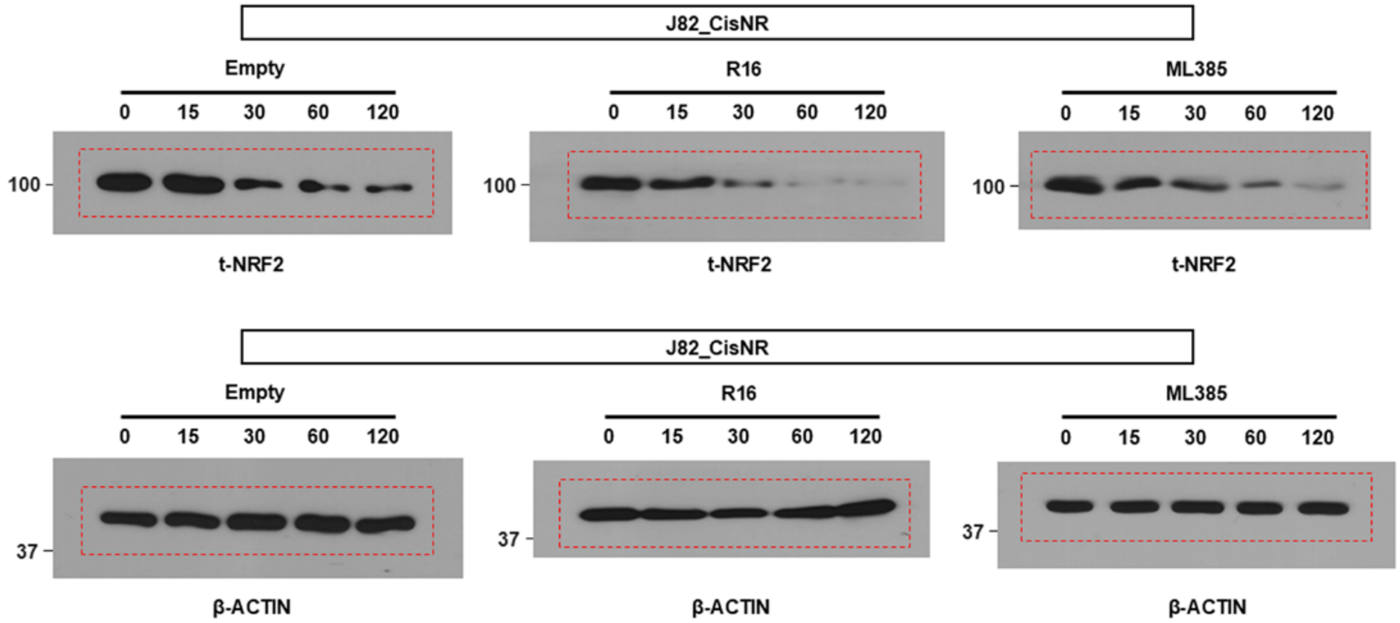
J82_CisNR
Empty
0 15 30 60 120
100
t-NRF2
R16
0 15 30 60 120
100
t-NRF2
ML385
0 15 30 60 120
100
t-NRF2
J82_CisNR
Empty
0 15 30 60 120
37
β-ACTIN
R16
0 15 30 60 120
37
β-ACTIN
ML385
0 15 30 60 120
37
β-ACTIN

Fig. 6l

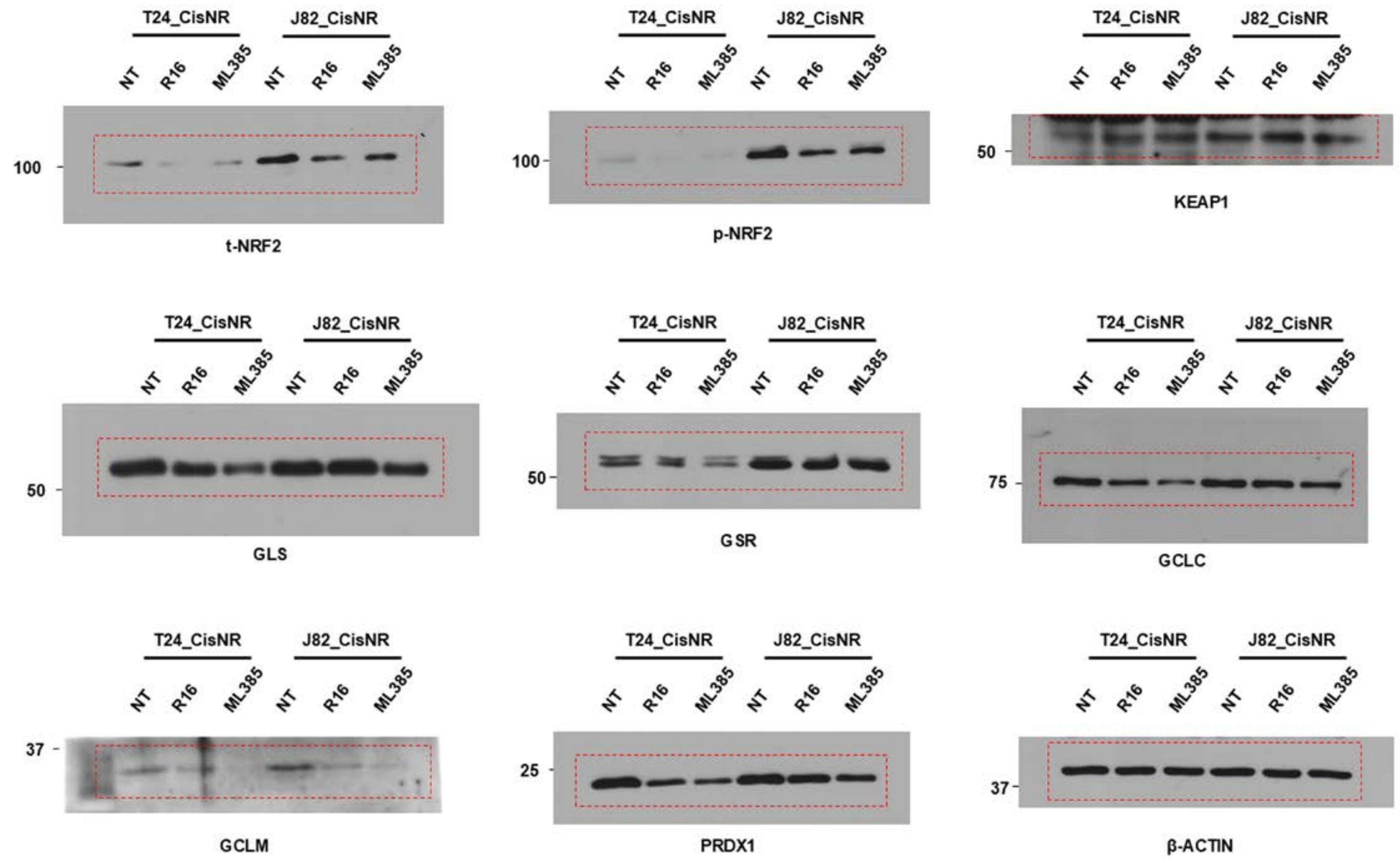
T24_CisNR
J82_CisNR
NT
R16
ML385
100
t-NRF2
p-NRF2
50
KEAP1
GLS
GSR
75
GCLC
37
GCLM
25
PRDX1
β-ACTIN

Fig. 7d

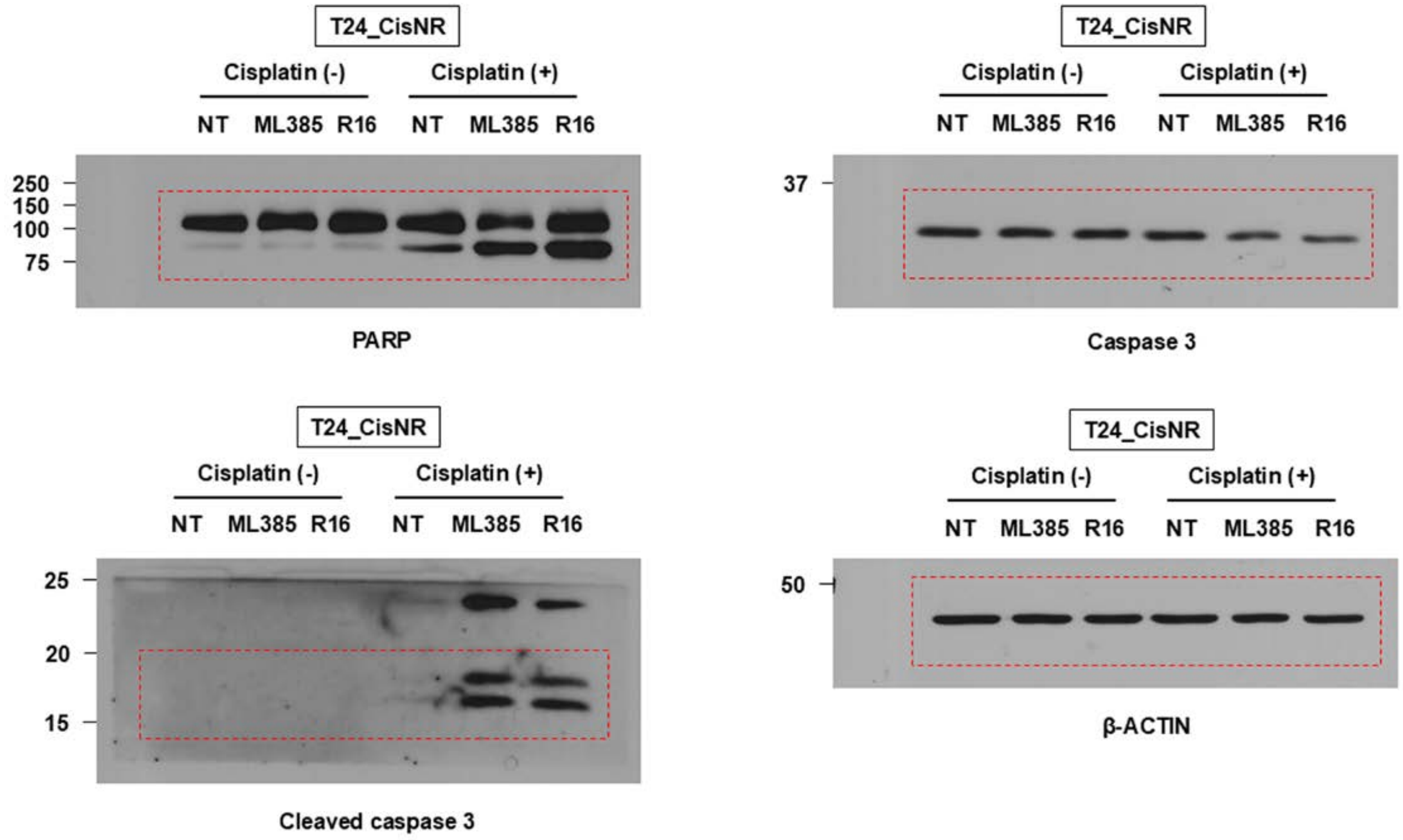
T24_CisNR
Cisplatin (-)
Cisplatin (+)
NT ML385 R16 NT ML385 R16
250
150
100
75
PARP
T24_CisNR
Cisplatin (-)
Cisplatin (+)
NT ML385 R16 NT ML385 R16
37
Caspase 3
T24_CisNR
Cisplatin (-)
Cisplatin (+)
NT ML385 R16 NT ML385 R16
25
20
15
Cleaved caspase 3
T24_CisNR
Cisplatin (-)
Cisplatin (+)
NT ML385 R16 NT ML385 R16
50
β-ACTIN

Supplementary Fig. 10g

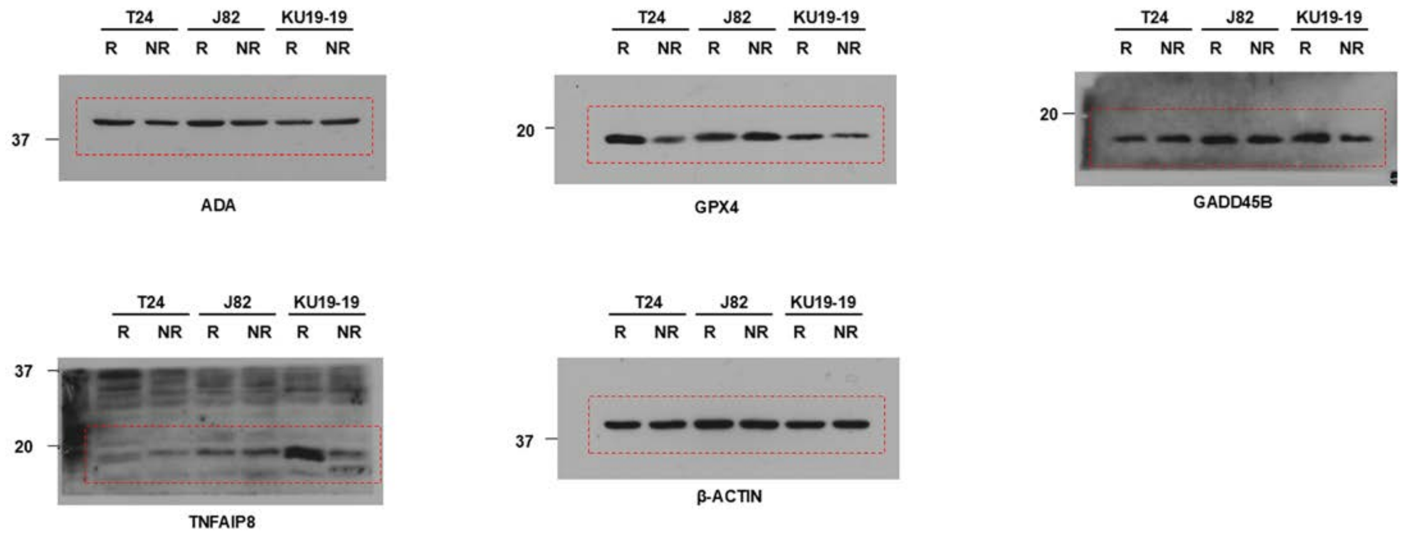

Supplementary Fig. 12e

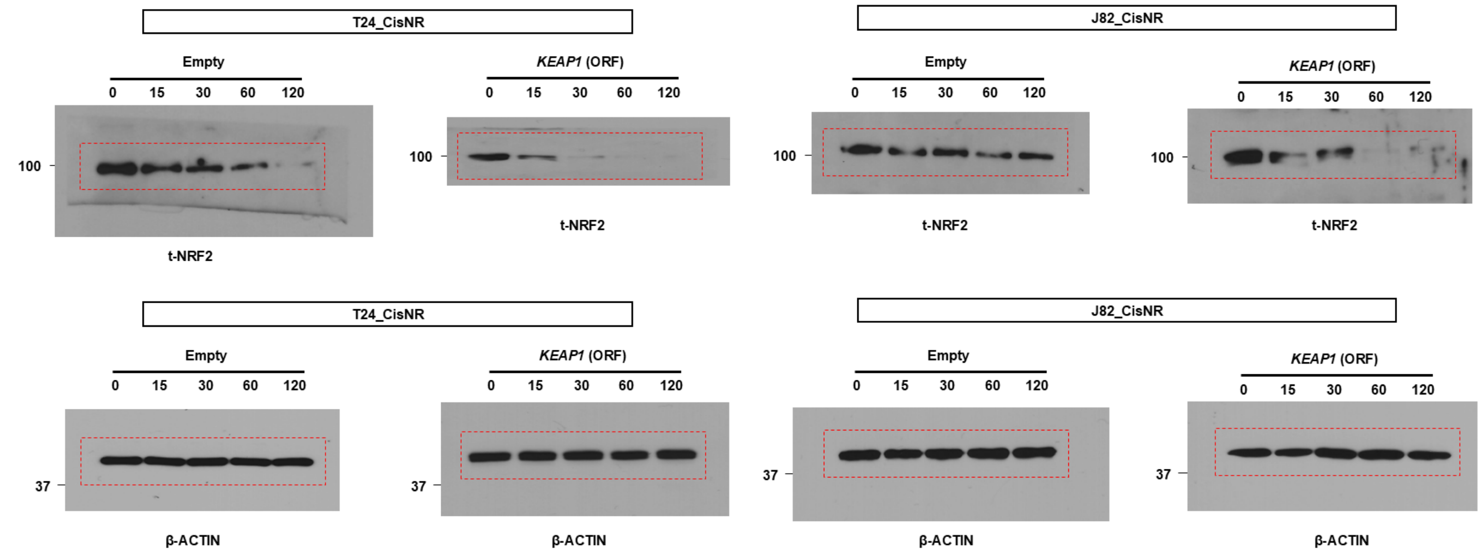

Supplementary Fig. 15g

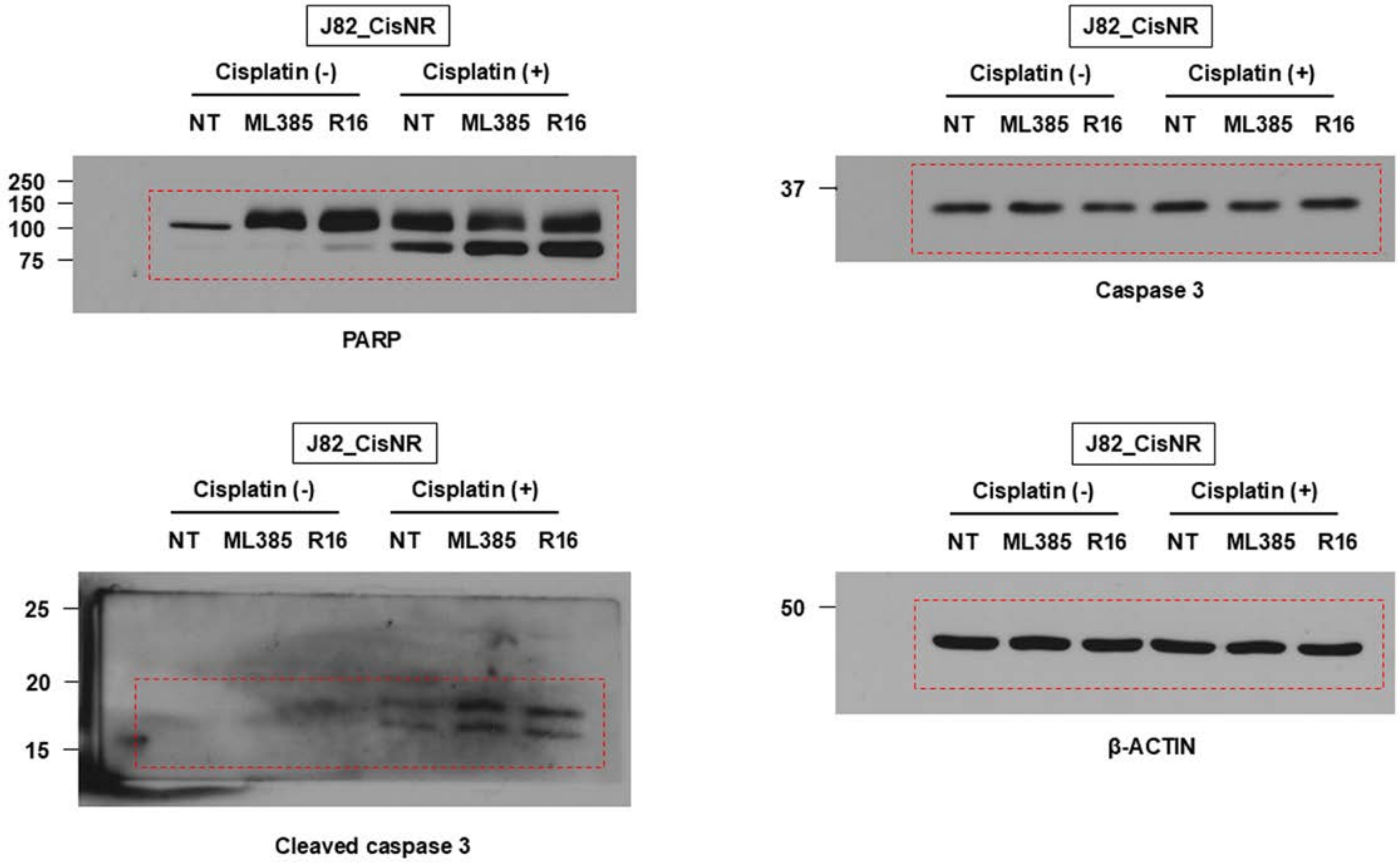

SUPPLEMENTARY REFERENCES

1. Jeong, E. M. *et al.* Real-Time Monitoring of Glutathione in Living Cells Reveals that High Glutathione Levels Are Required to Maintain Stem Cell Function. *Stem Cell Reports* **10**, 600-614 (2018).
2. Lim, J. *et al.* Glutathione dynamics determine the therapeutic efficacy of mesenchymal stem cells for graft-versus-host disease via CREB1-NRF2 pathway. *Science Advances* **6**, eaba1334 (2020).
3. Kim, Y. *et al.* Glutathione dynamics is a potential predictive and therapeutic trait for neoadjuvant chemotherapy response in bladder cancer. *Cell Rep Med* **4**, 101224 (2023).
4. Ju, H. *et al.* Activating transcription factor-2 supports the antioxidant capacity and ability of human mesenchymal stem cells to prevent asthmatic airway inflammation. *Exp Mol Med* **55**, 413-425 (2023).
5. Heo, J. *et al.* Sirt1 Regulates DNA Methylation and Differentiation Potential of Embryonic Stem Cells by Antagonizing Dnmt3l. *Cell Rep* **18**, 1930-1945 (2017).
6. Heo, J. *et al.* Phosphorylation of TFCP2L1 by CDK1 is required for stem cell pluripotency and bladder carcinogenesis. *EMBO Mol Med* **12**, e10880 (2020).
7. Heo, J. *et al.* The CDK1/TFCP2L1/ID2 cascade offers a novel combination therapy strategy in a preclinical model of bladder cancer. *Exp Mol Med* **54**, 801-811 (2022).
